# Synthesis of *N*-acyl carbazoles, phenoxazines and acridines from cyclic diaryliodonium salts

**DOI:** 10.3762/bjoc.20.2

**Published:** 2024-01-04

**Authors:** Nils Clamor, Mattis Damrath, Thomas J Kuczmera, Daniel Duvinage, Boris J Nachtsheim

**Affiliations:** 1 Institute for Organic and Analytical Chemistry, University of Bremen, Leobener Straße 7, D-28359 Bremen, Germany; 2 Institute for Inorganic and Crystallographic Chemistry, University of Bremen, Leobener Straße 7, 28359 Bremen, Germany

**Keywords:** carbazoles, heteroaromatics, iodanes, metal-catalyzed, one-pot reaction

## Abstract

*N*-Acyl carbazoles can be efficiently produced through a single-step process using amides and cyclic diaryliodonium triflates. This convenient reaction is facilitated by copper iodide in *p*-xylene, using the commonly found activating ligand diglyme. We have tested this method with a wide range of amides and iodonium triflates, proving its versatility with numerous substrates. Beyond carbazoles, we also produced a variety of other *N*-heterocycles, such as acridines, phenoxazines, or phenazines, showcasing the robustness of our technique. In a broader sense, this new method creates two C–N bonds simultaneously based on a mono-halogenated starting material, thus allowing heterocycle formation with diminished halogen waste*.*

## Introduction

**Scheme 1:**
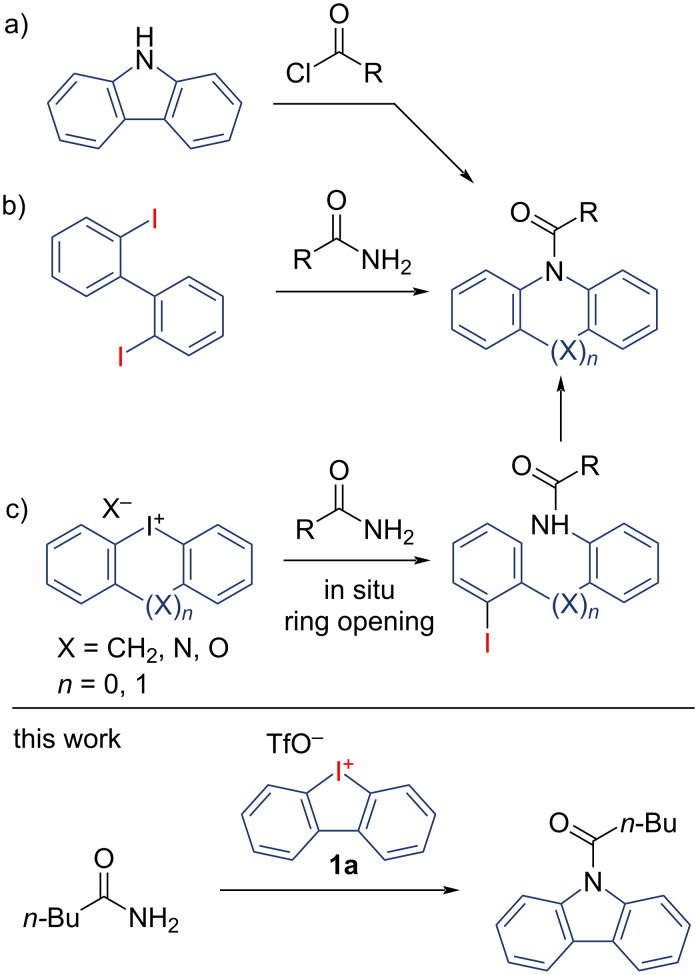
Examples for direct syntheses of *N*-acyl heteroaromatic compounds.

*N*-Acyl carbazoles are effective fluorophors, previously shown to exhibit strong organic phosphorescence when mixed with specific additives [1–5]. Carbazole units are also found in drugs and natural products. They are also used in electrochemistry and as reagents in transamidation reactions [6–12]. The traditional method to produce this versatile *N*-acyl carbazole motif involves combining 9*H*-carbazoles with acyl chlorides or similar activated acyl derivatives in the presence of a base (Scheme 1a) [13–14]. As an alternative, acyl carbazoles can be synthesized through step-wise metal-catalysed C–X-amidations followed by a ring-closure starting from 2,2'-diiodo-1,1'-biphenyls [15–17]. Related one-pot procedures are also described (Scheme 1b) [18–19].

Such 2,2´-dihalobiphenyls are established starting materials for synthesizing a variety of heterocycles. Usually, their utilization leads to the production of two equivalents of halogen salt waste. Their substitution with iodolium salts will be more sustainable since it reduces these unproductive halogenide salts by half. Due to our recent activity in the field of synthesis and applications of 5- and 6-membered cyclic iodonium salts, we searched for an efficient method to synthesize *N*-acyl carbazoles from readily available iodolium salt and amides via a ring-opening/intramolecular coupling cascade (Scheme 1c) [20–30]. Our group recently explored principle synthetic pathways of hetero- and carbocyclic 5- and 6-membered diaryliodonium salts [29,31], as well as Pd-catalysed methods for synthesizing *N*-aryl carbazoles [32]. Similar procedures were published for the Cu-catalysed synthesis of aryl carbazoles from amines as well as other heterocycles such as *N*-acyl acridanes with nitriles using cyclic iodonium salts by Wen and Chen [33–34].

## Results and Discussion

Initially, we investigated the synthesis of *N*-acyl carbazole by treatment of diaryliodonium salt **1a** with valeramide using Cu(I) catalysts [18]. The results are shown in Table 1. In the first experiments in *p*-xylene at 120 °C with DMEDA as *N,N*-ligand, only modest amounts of **2a** were observed (Table 1, entry 1). The predominant side products were 2,2'-diiodobiphenyl and 9*H*-carbazole. The formation of free carbazole indicated the formation and subsequent hydrolysis of **2a**. The presence of 2,2'-diiodobiphenyl suggests the reaction of **1a** with iodide released by each turnover of the desired reaction [35]. To mitigate this, using silver salts as iodide scavengers in the reaction was attempted but yielded none of the desired product (Table 1, entry 2). DMF as a solvent lowered the yield to 16% (Table 1, entry 3). Switching the catalyst system to Cu(OTf)_2_/glyme gave a significantly higher yield of 33% (Table 1, entry 4). Increasing the amount of iodolium salt to 1.5 equivalents yielded **2a** in 42% (Table 1, entry 5). Further increasing the amount of **1a** to 2 equivalents raised the yield only slightly (Table 1, entry 6), while finally exchanging the catalyst to CuI/diglyme at 15 mol % raised yields to synthetically useful 74% (Table 1, entry 7). The excess amount of **1a** was still necessary as a significant amount of iodobiphenyl is formed under the reaction conditions as a result of an undesired heterolytic iodine–carbon bond cleavage. Other carbonate bases and changing the Cu(I) source resulted in a complete collapse of reactivity. In a further experiment, we investigated the influence of iodide on the reaction to confirm whether or not diiodobiphenyl plays a role as an intermediate. The addition of potassium iodide leads to only diiodobiphenyl as the product. To confirm the mechanism of opening of the iodane, we used 2,2'-diiodobiphenyl as the starting material, leading to no formation of **2a**. Thus, we confirmed that our system does not activate 2,2'-diiodobiphenyl. Therefore, we applied the conditions described in Table 1, entry 7 for further investigation.

**Table 1:** Optimization of reaction conditions.^a^

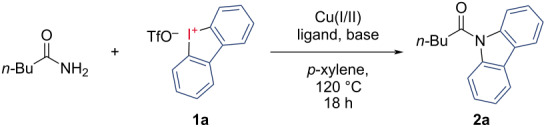					

	Catalyst (mol %)	Ligand (mol %)	Base (equiv)	Equiv of **1a**	Yield (%)

**1**	CuI	DMEDA (20)	K_3_PO_4_ (1.5)	1.0	22^b^
**2**	CuI (10) + 1 equiv AgNO_3_	DMEDA (20)	K_3_PO_4_ (1.5)	1.0	n.d.
**3**	CuI (10)	DMEDA (20)	K_2_CO_3_ (1.5)	1.0	16^b,c^
**4**	Cu(OTf)_2_ (10)	glyme (20)	K_2_CO_3_ (2.5)	1.0	33^b^
**5**	Cu(OTf)_2_ (10)	diglyme (20)	K_2_CO_3_ (2.5)	1.5	42^b^
**6**	Cu(OTf)_2_ (15)	diglyme (30)	K_2_CO_3_ (2.5)	2.0	44^b^
**7**	**CuI (15)**	**diglyme (30)**	**K** **_2_** **CO** **_3 _** **(3.0)**	**2.0**	**76** **^b^** **74** **^d^**
**8**	CuI (15)	diglyme (30)	Cs_2_CO_3_ (3.0)	2.0	–
**9**	CuCl	diglyme (30)	K_2_CO_3_ (3.0)	2.0	–
**10**	CuBr	diglyme (30)	K_2_CO_3_ (3.0)	2.0	–
**11**	CuI (15)	diglyme (30)	K_2_CO_3_ (3.0)	2.0	–^e^
**12**	CuI (15)	diglyme (30)	K_2_CO_3_ (3.0)	–	–^f^

^a^Common reaction conditions: 18 h at 120 °C, in degassed *p-*xylene under Ar atmosphere. ^b^Yields determined via GC–MS at a 100 µmol scale. ^c^Reaction carried out in DMF. ^d^Isolated yields on a 200 µmol scale. ^e^2.0 equiv of KI added. ^f^2,2-Diiodobiphenyl as starting material.

**Scheme 2:**
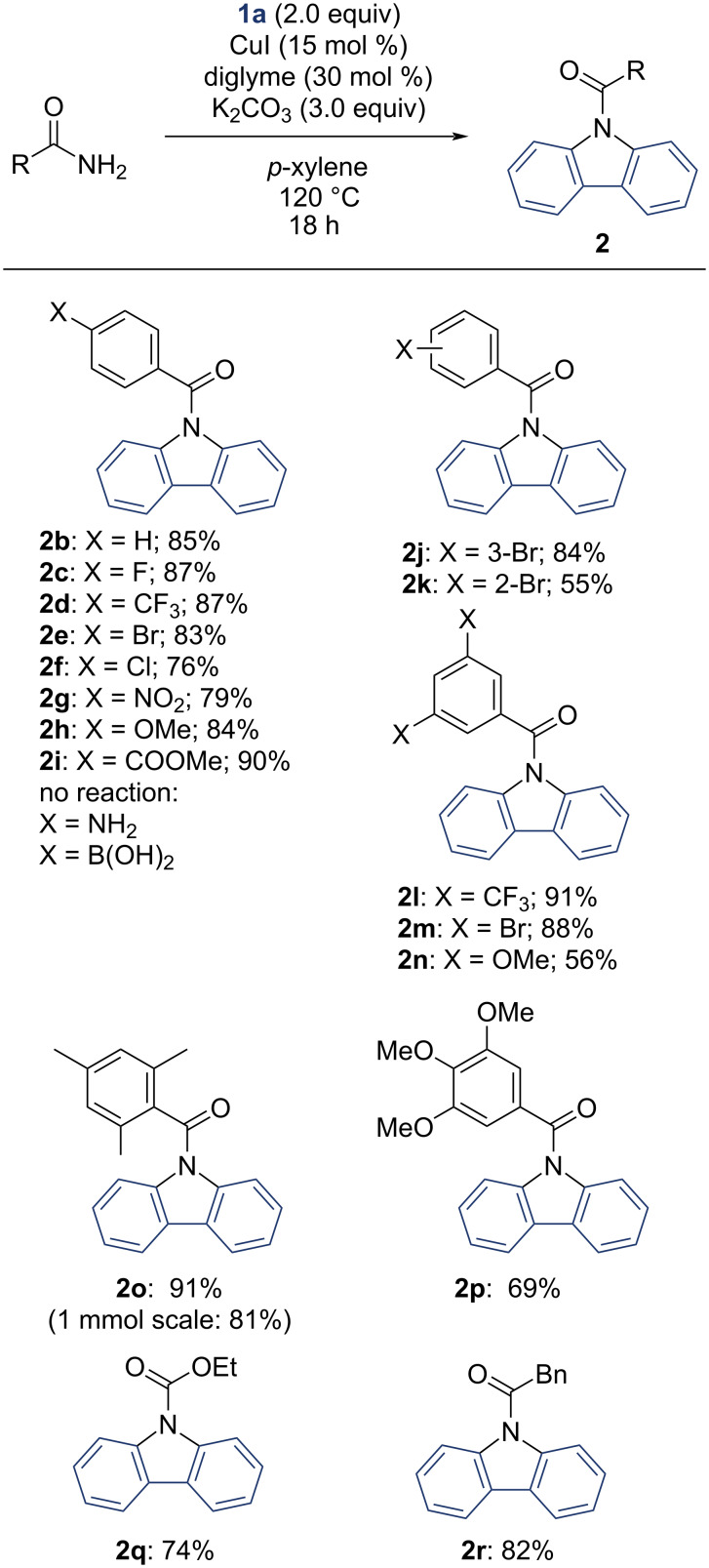
Scope of amides. ^a^Isolated yields, 200 µmol scale, all reactions carried out in *p*-xylene, with 1.00 equiv amide, 15 mol % CuI, 30 mol % diglyme, 3.00 equiv K_2_CO_3_ and 2.00 equiv dibenzo[*b,d*]iodol-5-ium trifluoromethanesulfonate.

With the optimized conditions in hand, the substrate scope was explored. The variations of amides are outlined in Scheme 2. Switching from valeramide to benzamide as a substrate gave a more advantageous yield of 85% of **2b**.

We tested *para*-substituted benzamides in the reaction to further assess the diversity of possible products. We obtained *p*-halogenide- and *p*-pseudohalogenide-substituted compounds **2c–g** in good yields of 76–87%. It is noteworthy that the *para*-chloro-substituted compound **2f** is a known fluorophore [5]. The reaction tolerated methoxy- and methyl ester-substitution to give **2h** and **2i** in 84% and 90% yields. *para*-Amino- and boronic acid-substituted benzamides did not react. While *meta*-bromo-substituted benzamide gave **2j** in 84% yield, *ortho*-bromination resulted in a diminished yield of **2k** (55%). We obtained 3,5-disubstituted *N*-acyl carbazoles **2l** and **2m** in 91% and 88% yields. The same disubstitution with electron-donating methoxy groups gave product **2n** in a diminished output of 56% yield. Other electron-rich 2,4,6-trimethyl- and 3,4,5-trimethoxy-substituted benzamides gave **2o** in 91% and **2p** in 69% yield. In an experiment at a larger scale (1 mmol), **2o** was still generated in 81%, which underscores the robustness of this method. Substrates with multiple electron-withdrawing substituents such as trifluoromethyl and bromide gave good yields. The ethyl carbamate **2q** proved to be a valid substrate for the reaction with a 74% yield. Phenylacetamide as the substrate resulted in the formation of **2r** in 82% yield.

**Scheme 3:**
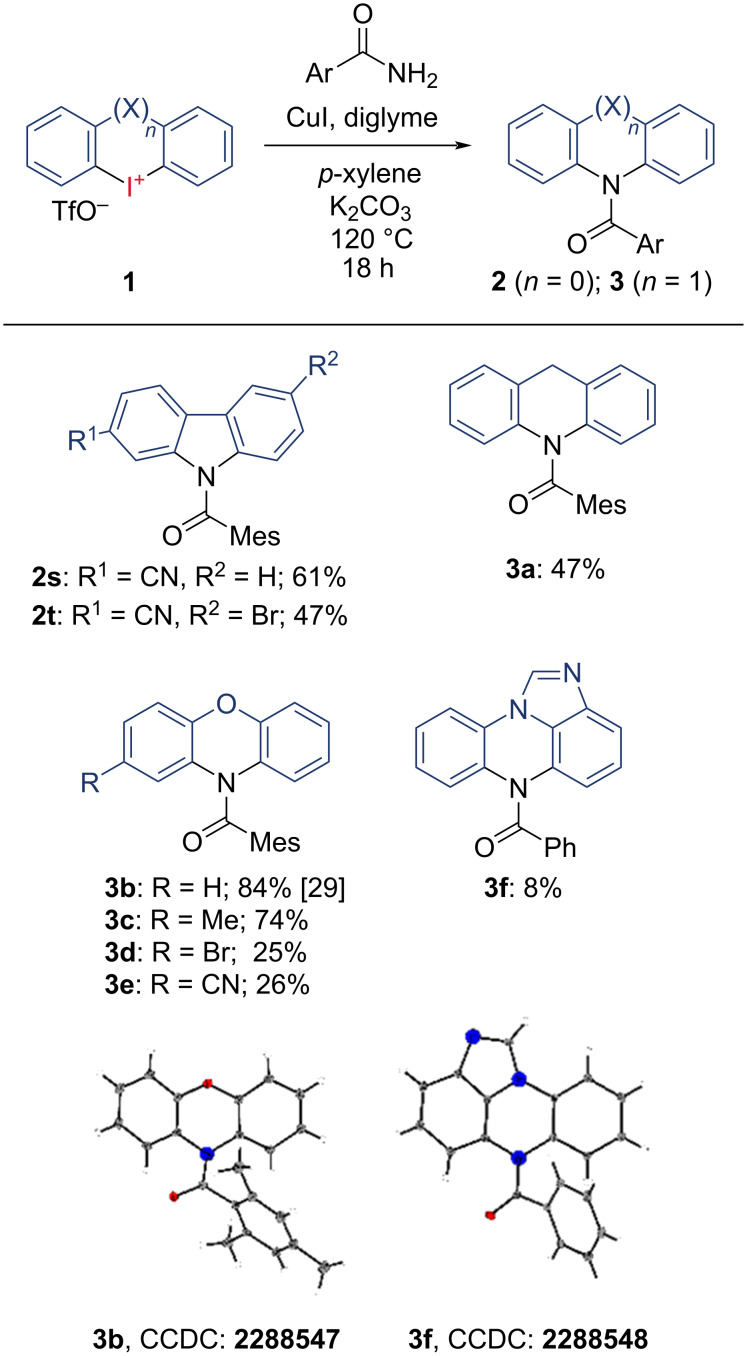
Scope of iodanes. ^a^Isolated yields, 200 µmol scale, all reactions carried out in *p*-xylene, with 1.00 equiv amide 15 mol % CuI, 30 mol % diglyme, 3.00 equiv K_2_CO_3_ and 2.00 equiv iodane. ^b^phenoxazines **3b–e** required a higher reaction temperature of 135 °C.

Next, we investigated structural variations in the cyclic iodonium salt. Due to the high reactivity of 2,4,6-trimethylbenzamide, we utilized this substrate as the nucleophile during these investigations. Scheme 3 displays the results. We started this investigation with the synthesis of nitrile-substituted carbazoles **2s** and **2t**, which are potent fluorophores. While **2s** was isolated in 61% yield, the additional 6-bromo-substituent diminished the yield of **2t** to 47%. When we subjected 6-membered 10*H*-dibenzo[*b*,*e*]iodinin-5-iums to our conditions, we synthesized the *N*-acyl dihydroacridane **3a** with a 47% yield. A method for the synthesis of similar annulated *N*-heterocycles from iodanes with nitriles is described by Chen et al. [34].

Next, we investigated O-bridged dibenzo[*b,e*][1,4]iodaoxin-5-ium salts as substrates, as was shown in a recent work [29]. Following the trend, we already observed for the 5-membered iodoliums, electron-donating groups are beneficial, while electron-withdrawing groups adversely affect their reactivity. Hence, we isolated unsubstituted and methyl-substituted *N*-acyl phenoxazines **3b** and **3c** in 84% [29] and 74% yields and their bromo and cyano variants **3d** and **3e** in lower 25% and 26% yields. Lastly, we synthesized **3f** using the standard procedure for iodoliums with more sophisticated benzimidazole-containing diaryliodonium salt, but we only observed a reproducible yield of 8%. The analysis of its solid-state structure showed the desired connectivity [36].

## Conclusion

In conclusion, we developed an effective method for synthesizing *N*-acyl carbazoles, phenoxazines, and acridines in a single-step reaction from 5- and 6-membered cyclic biaryliodonium salts. Based on the excellent synthetic availability of the underlying cyclic iodonium salts, this reaction provides fast and reliable access to these substrates, which are profound structural motifs with application in medicinal and materials chemistry.

## Supporting Information

File 1 Experimental part.
