# Supplementary material for: Synthesis of N-acyl carbazoles, phenoxazines and acridines from cyclic diaryliodonium salts
Source: Beilstein J Org Chem. 2024 Jan 4;20:12–6. doi: 10.3762/bjoc.20.2 (PMC10777325; doi:10.3762/bjoc.20.2)
Supplement: File 1 — Experimental part. [file Beilstein_J_Org_Chem-20-12-s001.pdf]

BEILSTEIN JOURNAL OF ORGANIC CHEMISTRY

Supporting Information

for

Synthesis of *N*-acyl carbazoles, phenoxazines and acridines from cyclic diaryliodonium salts

Nils Clamor, Mattis Damrath, Thomas J. Kuczmera, Daniel Duvinage and Boris J. Nachtsheim

Beilstein J. Org. Chem. **2024,** *20,* 12–16. doi:10.3762/bjoc.20.2

Experimental part

License and Terms: This is a supporting information file under the terms of the Creative Commons Attribution License (https://creativecommons.org/licenses/by/4.0). Please note that the reuse, redistribution and reproduction in particular requires that the author(s) and source are credited and that individual graphics may be subject to special legal provisions.
The license is subject to the *Beilstein Journal of Organic Chemistry* terms and conditions: (https://www.beilstein-journals.org/bjoc/terms)

Table of Contents

General information S1

Synthesis of *N*-Acylcarbazoles S2

GP1: General procedure for the synthesis of N-Acylcarbazoles **2** S2

GP2 Synthesis of dibenzo[b,d]iodol-5-ium trifluoromethanesulfonates **1** S2

Dibenzo[*b,d*]iodol-5-ium trifluoromethanesulfonate (**1a**) S3

3-Cyanodibenzo[*b,d*]iodol-5-ium trifluoromethanesulfonate (**1s**) S3

2-Bromo-7-cyanodibenzo[b,d]iodol-5-ium trifluoromethanesulfonate (**1t**) S3

1-(9*H*-Carbazol-9-yl)pentan-1-one (**2a**) S4

(9*H*-Carbazol-9-yl)(phenyl)methanone (**2b**) S4

(9*H*-Carbazol-9-yl)(4-fluorophenyl)methanone (**2c**) S4

(9*H*-Carbazol-9-yl)(4-(trifluoromethyl)phenyl)methanone (**2d**) S5

(4-Bromophenyl)(9*H*-carbazol-9-yl)methanone (**2e**) S5

(9*H*-Carbazol-9-yl)(4-chlorophenyl)methanone (**2f**) S5

(9*H*-Carbazol-9-yl)(4-nitrophenyl)methanone (**2g**) S6

(9*H*-Carbazol-9-yl)(4-methoxyphenyl)methanone (**2h**) S6

Methyl 4-(9*H*-carbazole-9-carbonyl)benzoate (**2i**) S6

(3-Bromophenyl)(9*H*-carbazol-9-yl)methanone (**2j**) S7

(2-Bromophenyl)(9*H*-carbazol-9-yl)methanone (**2k**) S7

(3,5-Bis(trifluoromethyl)phenyl)(9*H*-carbazol-9-yl)methanone (**2l**) S7

(9*H*-Carbazol-9-yl)(3,5-dibromophenyl)methanone (**2m**) S8

(9*H*-Carbazol-9-yl)(3,5-dimethoxyphenyl)methanone (**2n**) S8

(9*H*-Carbazol-9-yl)(mesityl)methanone (**2o**) S8

(9*H*-Carbazol-9-yl)(3,4,5-trimethoxyphenyl)methanone (**2p**) S9

Ethyl 9*H*-carbazole-9-carboxylate (**2q**) S9

1-(9*H*-Carbazol-9-yl)-2-phenylethan-1-one (**2r**) S9

9-(2,4,6-Trimethylbenzoyl)-9*H*-carbazole-2-carbonitrile (**2s**) S10

6-Bromo-9-(2,4,6-trimethylbenzoyl)-9*H*-carbazole-2-carbonitrile (**2t**) S10

Acridin-10(9*H*)-yl(mesityl)methanone (**3a**) S10

Mesityl(10*H*-phenoxazin-10-yl)methanone (**3b**) S11

Mesityl(2-methyl-10*H*-phenoxazin-10-yl)methanone (**3c**) S11

(2-Bromo-10*H*-phenoxazin-10-yl)(mesityl)methanone (**3d**) S12

10-(2,4,6-Trimethylbenzoyl)-10*H*-phenoxazine-2-carbonitrile (**3e**) S12

(6*H*-Imidazo[4,5,1-de]henazine-6-yl)(phenyl)methanone (**3f**) S12

NMR-Spectra S14

2-Bromo-7-cyanodibenzo[*b,d*]iodol-5-ium trifluoromethanesulfonate (**1t**) S14

(4-Bromophenyl)(9*H*-carbazol-9-yl)methanone (**2e**) S15
(9*H*-Carbazol-9-yl)(4-nitrophenyl)methanone(**2g**)... S16
Methyl 4-(9*H*-carbazole-9-carbonyl)benzoate (**2i**) S17
(3-Bromophenyl)(9*H*-carbazol-9-yl)methanone (**2j**) S18
(2-Bromophenyl)(9*H*-carbazol-9-yl)methanone (**2k**) S19
3,5-Bis(trifluoromethyl)phenyl)(9*H*-carbazol-9-yl)methanone (**2l**) S20
(9*H*-Carbazol-9-yl)(3,5-dibromophenyl)methanone (**2m**)..................................... S22
(9*H*-Carbazol-9-yl)(3,5-dimethoxyphenyl)methanone (**2n**) S23
(9*H*-Carbazol-9-yl)(mesityl)methanone (**2o**) S24
(9*H*-Carbazol-9-yl)(3,4,5-trimethoxyphenyl)methanone (**2p**) S25
1-(9*H*-Carbazol-9-yl)-2-phenylethan-1-one (**2r**) S26
9-(2,4,6-Trimethylbenzoyl)-9*H*-carbazole-2-carbonitrile (**2s**) S27
6-Bromo-9-(2,4,6-trimethylbenzoyl)-9*H*-carbazole-2-carbonitrile (**2t**) S28
Acridin-10(9*H*)-yl(mesityl)methanone (**3a**).. S29
Mesityl(10*H*-phenoxazin-10-yl)methanone (**3b**).. S30
Mesityl(2-methyl-10*H*-phenoxazin-10-yl)methanone (**3c**)..................................... S31
(2-Bromo-10*H*-phenoxazin-10-yl)(mesityl)methanone (**3d**) S32
10-(2,4,6-Trimethylbenzoyl)-10*H*-phenoxazine-2-carbonitrile (**3e**) S33
(6*H*-Imidazo[4,5,1-de]phenazin-6-yl)(phenyl)methanone (**3f**)............................... S34
Crystallographic data S35

General information

Unless otherwise noted, water- or oxygen-sensitive reactions were carried out under Argon atmosphere using standard Schlenck techniques. All chemicals were purchased from commercial suppliers (abcr, Acros Organics, Alfa Aesar, Apollo Scientific, Carbolution Chemicals, Fluorochem, Sigma Aldrich, TCI) and either used as received or purified according to Purification of Common Laboratory Chemicals. Dry solvents were obtained from an inert PS-MD-6 solvent purification system, xylene was dried by filtering through anhydrous Alumina and stored over 4 Å molecular sieves. Degassing of Xylene was achieved by sparging with Argon. Yields refer to isolated yields of compounds estimated to be >95% pure as determined by ^{1}H-NMR spectroscopy.

Thin layer chromatography was performed on fluorescence indicator marked precoated silica gel 60 plates (Macherey-Nagel, ALUGRAM Xtra SIL G/UV254) and visualized by UV light (254 nm/366 nm). Flash column chromatography was performed on silica gel (0.040 – 0.063 mm) with the solvents given in the procedures.

Reactions that required heating were either heated using an oil bath, if the reaction was performed in a flask or in a heating block, if the reaction was performed in a screw cap vial.

^{1}H- and ^{13}C-NMR spectra were recorded on Bruker Avance 360NB and Bruker AvanceNeo600-spectrometers. Chemical shifts for ^{1}H-NMR spectra were reported as δ (parts per million) relative to the residual signal of $CHCl_3$ at 7.26 ppm (s) or d_6-DMSO at 2.50 ppm (quin). Chemical shifts for ^{13}C-NMR spectra were reported as δ (parts per million) relative to the signal of $CDCl_3$ at 77.0 ppm (t) or d_6-DMSO at 39.5 ppm (sept.). The following abbreviations were used to describe splitting patterns: br = broad, s = singlet, d = doublet, t = triplet, q = quartet, quin = quintet, sept = septet, m = multiplet. Coupling constants J are given in Hertz.

High resolution (HR) EI mass spectra were recorded on the double focussing mass spectrometer ThermoQuest MAT 95 XL from Finnigan MAT.APCI mass spectra were recorded on a Advion Expression CMSL via ASAP probe or direct inlet. HR-ESI and APCI mass spectra were recorded on a Bruker impact II. All signals were reported with the quotient from mass to charge m/z. IR spectra were recorded on a Nicolet Thermo iS10 scientific spectrometer with a diamond ATR unit. The absorption bands were reported in cm^{-1}. Melting points were determined on a Büchi M-5600 Melting Point apparatus with a heating rate of 5 °C/min. The melting points were reported in °C. X-ray diffractometry was carried out in a Bruker Venture D8. Single Crystals were obtained *via* recrystallization from Cyclohexane/Ethyl acetate mixtures.

Synthesis of *N*-Acylcarbazoles

GP1: General procedure for the synthesis of N-Acylcarbazoles 2

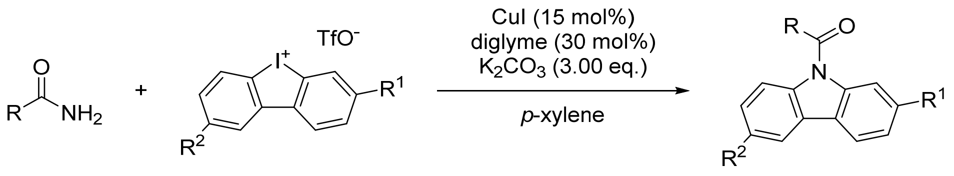

In a heat gun-dried and argon-flushed Schlenck-tube, CuI (5.7 mg, 30.0 µmol, 0.15 equiv.) was suspended in dried and degassed *p*-xylene (2.0 mL, 100 mM) and diglyme (8.59 µL, 60.0 µmol, 0.30 equiv.) was added. The mixture was stirred for 10 min at 80 °C in an oil bath. Amide (200 µmol, 1.00 equiv.) iodonium salt **1** (400 µmol, 2.00 equiv.) and K_2CO_3 (82.9 mg, 600 µmol, 3.00 equiv.) were added and the oil bath was heated to 120 °C. Upon reaching 120 °C, the reaction vessel was sealed and the mixture was stirred vigorously for 18 h. Completion of the reaction was verified *via* TLC and after the starting material was consumed, the mixture was allowed to cool to room temperature and saturated aqueous solution of $NaHCO_3$ (2 mL) was added.

Phases were separated and The aqueous phase was extracted with EtOAc (4 x 2 mL). The combined organic phases were dried over Na_2SO_4 and solvents were removed under reduced pressure. Column chromatography (SiO_2 / Cyclohexane: EtOAc) was carried out to yield products.

GP2 Synthesis of dibenzo[*b,d*]iodol-5-ium trifluoromethanesulfonates 1

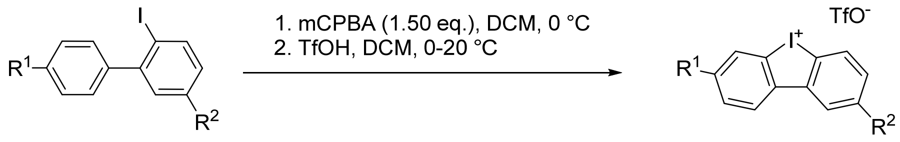

In a modified literature procedure[1] 2-iodobiphenyls were dissolved in DCM (0.4 M) and cooled in an ice bath. *m*CPBA (1.50 equiv. 60 wt %) was added to the stirred solution and the reaction was stirred under cooling. Upon consumption of the starting material, TfOH (2.00 equiv.) was added to the reaction dropwise, and the reaction was allowed to reach room temperature. The mixture was concentrated *in vacuo*, and Diethyl ether was added to precipitate the product. The products may be purified *via* column chromatography (SiO_2/DCM:MeOH) followed by dissolving in MeOH and precipitation with Et_2O.

Dibenzo[*b,d*]iodol-5-ium trifluoromethanesulfonate (1a)

Synthesized according to **GP2** from 2-iodo-1,1'-biphenyl (600 mg, 2.14 mmol, 1.00 equiv.). Column chromatography (SiO_2 / DCM:MeOH (95:5 (v:v))) followed by precipitation from MeOH by addition of Et_2O gave the product as an off-white solid (817 mg, 1.91 mmol, 89%)

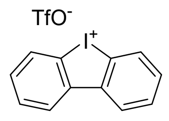

1a

^{1}H NMR (360 MHz, DMSO-*d*$_6$): δ = 8.55 – 8.41 (m, 2H), 8.27 – 8.13 (m, 2H), 7.93 – 7.79 (m, 2H), 7.71 (td, *J* = 8.0, 2.2 Hz, 2H) ppm. **^{13}C NMR (91 MHz, DMSO-*d*$_6$):** δ = 141.7, 131.1, 130.7, 130.6, 127.0, 121.6 ppm Signal for TfO$^-$ was not observed; **^{19}F NMR (565 MHz, DMSO-d6):** δ = -77.8 ppm; **FTIR (ATR, neat, cm^{-1}):** $\tilde{\nu}$ = 3093 (w), 3005 (w), 1586 (w), 1457 (m), 1443 (m), 1269 (s), 1233 (s), 1221 (s), 1177 (m), 1157 (m), 1025 (m); **MS(APCI):** *m/z* = 278.8 [M-TfO$^-$]$^+$ Mp: 238-242°C; The analytical data is in accordance with the literature[1]

3-Cyanodibenzo[*b,d*]iodol-5-ium trifluoromethanesulfonate (1s)

Synthesized according to **GP2** from 2'-iodo-[1,1'-biphenyl]-4-carbonitrile (350 mg, 1.15 mmol, 1.00 equiv.). Column chromatography (SiO_2/ DCM:MeOH) followed by precipitation from MeOH by addition of Et_2O gave the product as an off-white solid (374 mg, 826 mmol, 72%)

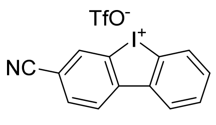

1s

^{1}H NMR (600 MHz, DMSO-*d*$_6$): δ = 8.7 (d, *J* = 8.3 Hz, 1H), 8.6 (dd, *J* = 8.0, 1.6 Hz, 1H), 8.5 (d, *J* = 1.6 Hz, 1H), 8.4 (dd, *J* = 8.2, 1.6 Hz, 1H), 8.3 – 8.2 (m, 1H), 8.0 – 7.9 (m, 1H), 7.8 (ddd, *J* = 8.6, 7.2, 1.4 Hz, 1H) ppm; **^{13}C NMR (151 MHz, DMSO-*d*$_6$):** δ = 146.5, 140.8, 134.8, 134.7, 133.0, 131.4, 131.1, 128.7, 127.8, 123.8, 122.3, 118.0, 112.9 ppm Signal for TfO$^-$ was not observed; **^{19}F NMR (565 MHz, DMSO-d6):** δ = -77.8 ppm; **FTIR (ATR, neat, cm^{-1}):** $\tilde{\nu}$ = 3099 (w), 2323 (w), 1770 (w), 1585 (w), 1456 (m) ,1439 (w), 1394 (w), 1270 (m), 1247 (s), 1223 (s), 1161 (s); **MS(APCI):** *m/z* = 304.0 [M-TfO$^-$]$^+$ Mp: decomp>288°C; The analytical data is in accordance with the literature[2]

2-Bromo-7-cyanodibenzo[*b,d*]iodol-5-ium trifluoromethanesulfonate (1t)

Synthesized according to **GP2** from 5'-bromo-2'-iodo-[1,1'-biphenyl]-4-carbonitrile (827 mg, 2.15 mmol, 1.00 equiv.). Column chromatography (SiO_2/ DCM:MeOH) followed by precipitation from MeOH by addition of Et_2O gave the product as a colourless solid (962 mg, 1.81 mmol, 84%)

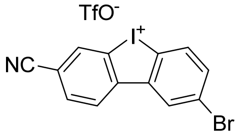

1t

^{1}H NMR (600 MHz, CDCl3): δ = 8.89 (d, *J* = 2.1 Hz, 1H), 8.75 (d, *J* = 8.3 Hz, 1H), 8.52 (d, *J* = 1.5 Hz, 1H), 8.34 (dd, *J* = 8.2, 1.5 Hz, 1H), 8.13 (d, *J* = 8.7 Hz, 1H), 7.97 (dd, *J* = 8.7, 2.1 Hz, 1H) ppm; **^{13}C NMR (151 MHz, DMSO-*d*$_6$):** δ = 144.7, 142.5, 134.9, 134.4, 134.1, 132.3, 130.7, 127.9, 125.1, 122.3, 122.0, 117.4, 113.0 ppm Signal for TfO$^-$ was not observed; **^{19}F NMR (565 MHz, DMSO-*d*$_6$):** δ = -77.7 ppm; **FTIR (ATR, neat, cm^{-1}):**

$\tilde{\nu}$ = 3106 (w), 2238 (w), 1759 (w), 1551 (w), 1474 (w), 1448 (w), 1383 (w), 1275 (s), 1241 (s), 1221 (s); **HR-MS (ESI):** m/z $[M-TfO^-]^+$ calcd. for: $C_{16}H_6BrIN^+$: 381.8723, found 381.8716; Mp: decomp> 330 °C

1-(9*H*-Carbazol-9-yl)pentan-1-one (2a)

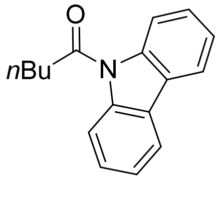

2a

Prepared according to **GP1** from Valeramide (20.2 mg, 200 µmol, 1.00 equiv.) and **1a** (171 mg, 400 µmol, 2.00 equiv.). Column chromatography (SiO_2/Cyclohexane:EtOAc 97:3 (V:V)) gave the product as a colourless solid (37.3 mg, 148 µmol, 74%)

^{1}H NMR (600 MHz, $CDCl_3$): δ = 8.23 (d, *J* = 8.4 Hz, 2H), 8.01 (dt, *J* = 7.6, 1.0 Hz, 2H), 7.49 (ddd, *J* = 8.5, 7.3, 1.4 Hz, 2H), 7.39 (td, *J* = 7.5, 0.9 Hz, 2H), 3.25 – 3.09 (m, 2H), 1.93 (dd, *J* = 8.3, 6.6 Hz, 2H), 1.65 – 1.50 (m, 2H), 1.03 (t, *J* = 7.4 Hz, 3H) ppm;**^{13}C NMR (151 MHz, $CDCl_3$):** δ = 173.5, 138.7, 127.4, 126.5, 123.7, 119.9, 116.6, 39.0, 26.9, 22.5, 14.1 ppm; **FTIR (ATR, neat, cm^{-1}):** $\tilde{\nu}$ = 3062 (w), 3028 (w); **MS (APCI):** m/z = 252.2 $[M+H]^+$; **Mp.:** T = 85.4-85.6 °C; The analytical data is in accordance with the literature[3]

(9*H*-Carbazol-9-yl)(phenyl)methanone (2b)

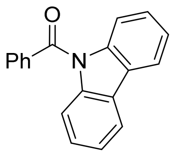

2b

Prepared according to **GP1** from Benzamide (24.2 mg, 200 µmol, 1.00 equiv.) and **1a** (171 mg, 400 µmol, 2.00 equiv.). Column chromatography (SiO_2/Cyclohexane:EtOAc 97:3 (V:V)) gave the product as colourless solid (46.1 mg, 170 µmol, 85%).

^{1}H NMR (600 MHz, $CDCl_3$): δ = 8.04 – 7.99 (m, 2H), 7.73 (dd, *J* = 8.2, 1.3 Hz, 2H), 7.69 – 7.63 (m, 1H), 7.57 – 7.49 (m, 4H), 7.44 – 7.29 (m, 4H) ppm;^{13}C **NMR (151 MHz, $CDCl_3$):** δ = 169.8, 139.3, 135.9, 132.5, 129.2, 129.0, 126.9, 126.2, 123.5, 120.0, 115.9 ppm; **FTIR (ATR, neat, cm^{-1}):** $\tilde{\nu}$ = 3062 (w), 3028 (w), 1911 (w), 1675 (s), 1440 (s), 1327 (m), 1301 (m), 1231 (m), 1070 (m), **MS (APCI):** m/z = 272.2 $[M+H]^+$ **Mp.:** T = 99.3-100.4 °C; The analytical data is in accordance with the literature[4]

(9*H*-Carbazol-9-yl)(4-fluorophenyl)methanone (2c)

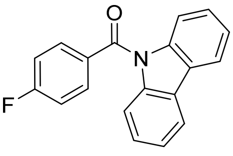

2c

Prepared according to **GP1** from 4-fluorobenzamide (27.8 mg, 200 µmol, 1.00 equiv.) and **1a** (171 mg, 400 µmol, 2.00 equiv.). Column chromatography (SiO_2/Cyclohexane:EtOAc 97:3 (V:V)) gave the product as a colourless solid (50.1 mg, 173 µmol, 87%)

^{1}H NMR (600 MHz, $CDCl_3$): δ = 8.04 – 7.99 (m, 2H), 7.76 (dd, J = 8.8, 5.3 Hz, 2H), 7.55 – 7.48 (m, 2H), 7.43 – 7.29 (m, 4H), 7.24 – 7.18 (m, 4H) ppm; **^{13}C NMR (151 MHz, $CDCl_3$):** δ = 168.6, 166.2, 164.6, 139.2, 132.0 (d, J = 8.9 Hz), 126.9, 126.2, 123.6, 120.1, 116.3 (d, J = 22.1 Hz), 115.8 ppm;**^{19}F NMR (565 MHz, $CDCl_3$):** δ = -105.5 ppm; **FTIR (ATR, neat, cm^{-1}):** $\tilde{\nu}$ = 3116 (w), 3061 (w), 2962 (w), 2922 (w), 2851 (w), 1915 (w), 1682 (s), 1601 (s), 1507 (m), 1444 (m), 1324 (s), **MS (APCI):** m/z = 290.2 $[M+H]^+$; **Mp:** T = 142-143 °C; Analytical data are in accordance to the literature[5, 6]

(9*H*-Carbazol-9-yl)(4-(trifluoromethyl)phenyl)methanone (2d)

Prepared according to **GP1** from 4-(trifluoromethyl)benzamide (37.8 mg, 200 µmol, 1.00 equiv.) and **1a** (171 mg, 400 µmol, 2.00 equiv.). Column chromatography (SiO_2/Cyclohexane:EtOAc 97:3 (V:V)) gave the product as a colourless solid (59.1 mg, 174 µmol, 87%)

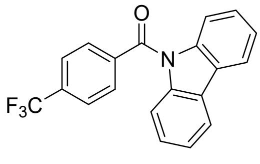

2d

^{1}H NMR (600 MHz, CDCl$_3$): δ = 8.02 (ddd, *J* = 7.7, 1.5, 0.7 Hz, 2H), 7.86 – 7.77 (m, 4H), 7.52 – 7.46 (m, 2H), 7.41 – 7.32 (m, 4H) ppm; **^{13}C NMR (151 MHz, CDCl$_3$):** δ = 168.1, 139.1, 138.8, 133.9 (q, *J* = 33.0 Hz), 129.4, 127.0, 126.3, 126.0 (d, *J* = 3.7 Hz), 123.9, 120.0, 115.8 ppm; **^{19}F NMR (565 MHz, CDCl$_3$):** δ = -62.9 ppm; **FTIR (ATR, neat, cm^{-1}):** $\tilde{\nu}$ = 3050 (w), 2962 (w), 2924 (w), 1745 (m), 1676 (s), 1616 (w), 1585 (w), 1489 (w), 1477 (w), 1444 (m); **MS (APCI):** m/z = 340.0 $[M]^{\bullet+}$; **Mp:** T = 144-145 °C; Analytical data are in accordance to the literature[7]

(4-Bromophenyl)(9*H*-carbazol-9-yl)methanone (2e)

Prepared according to **GP1** from 4-bromobenzamide (40.0 mg, 200 µmol, 1.00 equiv.) and **1a** (171 mg, 400 µmol, 2.00 equiv.). Column chromatography (SiO_2/Cyclohexane:EtOAc 97:3 (V:V)) gave the product as a colourless solid (58.3 mg, 166 µmol, 83%) as well as 9*H*-Carbazole (2.4 mg, 14.4 mmol, 7%).

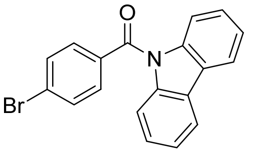

2e

^{1}H NMR (600 MHz, CDCl$_3$): δ = 8.06 – 7.98 (m, 2H), 7.68 (d, *J* = 8.5 Hz, 2H), 7.61 (d, *J* = 8.5 Hz, 2H), 7.56 – 7.50 (m, 2H), 7.42 – 7.30 (m, 4H) ppm ; **^{13}C NMR (151 MHz, CDCl$_3$):** δ = 168.7, 139.1, 134.6, 132.4, 130.9, 127.5, 127.0, 126.2, 123.8, 120.1, 115.8 ppm; **FTIR (ATR, neat, cm^{-1}):** $\tilde{\nu}$ =3103 (w), 3033 (w), 1674 (s), 1587 (s), 1476 (m), 1440 (m), 1450 (m), 1352 (m), 1342 (w), 1299 (s); **HRMS (APCI):** *m/z* $[M]^{\bullet+}$ calcd. for: $C_{19}H_{12}BrNO^+$ m/z = 349.0097, found 349.0094; **Mp:** T = 154-155 °C; Analytical data are in accordance with the literature [8]

(9*H*-Carbazol-9-yl)(4-chlorophenyl)methanone (2f)

Prepared according to **GP1** from 4-chlorobenzamide (31.1 mg, 200 µmol, 1.00 equiv.) and **1a** (171 mg, 400 µmol, 2.00 equiv.). Column chromatography (SiO_2/Cyclohexane:EtOAc 97:3 (V:V)) gave the product as a colourless solid (46.7 mg, 153 µmol,76%) as well as 9*H*-Carbazole (5.70 mg, 34.1 µmol, 14%)

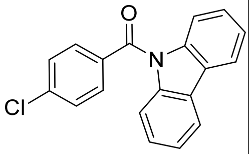

2f

^{1}H NMR (600 MHz, CDCl$_3$): δ = 8.04 – 8.00 (m, 2H), 7.68 (d, *J* = 8.5 Hz, 2H), 7.58 – 7.47 (m, 4H), 7.36 (m, 4H) ppm; **^{13}C NMR (151 MHz, CDCl$_3$):** δ = 168.6, 139.1, 139.0, 134.1, 130.8, 129.4, 127.0, 126.2, 123.8, 120.1, 115.8 ppm; **FTIR (ATR, neat, cm$_{-1}$):** $\tilde{\nu}$ = 3084 (w), 3058 (w), 3042 (w), 1677 (s), 1590 (m), 1476 (m), 1450 (m), 1441 (s), 1326 (s), 1300 (s); **MS (APCI):** m/z = 306.0 $[M+H]^+$; **Mp:** T = 156-157 °C; The analytical data are in accordance to the literature[9]

(9*H*-Carbazol-9-yl)(4-nitrophenyl)methanone(2g)

Prepared according to **GP1** from 4-nitrobenzamide (33.2 mg, 200 µmol, 1.00 equiv.) and **1a** (171 mg, 400 µmol, 2.00 equiv.). Column chromatography (SiO_2/Cyclohexane:EtOAc 97:3 (V:V)) gave the product as a colourless solid (50.1 mg, 158 µmol, 79%)

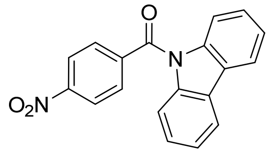

2g

^{1}H NMR (600 MHz, $CDCl_3$): δ = 8.43 – 8.34 (m, 2H), 8.03 (dt, *J* = 7.8, 1.0 Hz, 2H), 7.93 – 7.85 (m, 2H), 7.47 (d, *J* = 8.2 Hz, 2H), 7.40 (td, *J* = 7.4, 1.1 Hz, 2H), 7.34 (ddd, *J* = 8.5, 7.2, 1.3 Hz, 2H) ppm**; ^{13}C NMR (151 MHz, $CDCl_3$):** δ = 167.4, 150.0, 141.5, 138.8, 130.1, 127.3, 126.5, 124.4, 124.3, 120.3, 115.9, 110.7 ppm; **FTIR (ATR, neat, cm^{-1}):** $\tilde{\nu}$ = 3417 (w), 3105 (w), 3049 (w), 3049 (w), 2961 (w), 2853 (w), 1739 (m), 1674 (s), 1602 (m), 1523 (s), 1477 (m), 1443 (s); **HRMS (APCI):** *m/z* $[M]^{•+}$: calcd. For $C_{15}H_{13}N_2O_3^+$:316.0842, found 316.0839; **Mp:** T= 153-154 °C;

(9*H*-Carbazol-9-yl)(4-methoxyphenyl)methanone (2h)

Prepared according to **GP1** from 4-methoxybenzamide (30.2 mg, 200 µmol, 1.00 equiv.) and **1a** (171 mg, 400 µmol, 2.00 equiv.). Column chromatography (SiO_2/Cyclohexane:EtOAc 97:3 (V:V)) gave the product as a colourless solid (50.4 mg, 167 µmol, 84%)

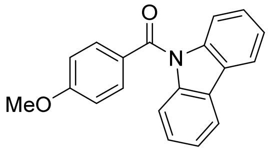

2h

^{1}H NMR (600 MHz, $CDCl_3$): δ = 8.05 – 7.99 (m, 1H), 7.76 – 7.71 (m, 1H), 7.58 – 7.53 (m, 1H), 7.38 – 7.32 (m, 2H), 7.00 (d, *J* = 8.8 Hz, 1H), 3.92 (s, 1H) ppm;**^{13}C NMR (151 MHz, $CDCl_3$):** δ = 169.3, 163.4, 139.5, 131.9, 127.7, 126.7, 125.9, 123.2, 120.0, 115.7, 114.3, 55.7 ppm; **FTIR (ATR, neat, cm^{-1}):** $\tilde{\nu}$ = 3064 (w), 3010 (w), 2971 (w), 2840 (w), 1669 (s), 1602 (s), 1506 (m), 1489 (w), 1450 (w), 1441 (m); **MS (APCI):** m/z = 302.1 $[M+H]^+$; **Mp:** T = 134-135 °C; Analytical data are in accordance to the literature.[7]

Methyl 4-(9*H*-carbazole-9-carbonyl)benzoate (2i)

Prepared according to **GP1** from methyl 4-carbamoylbenzoate (35.8 mg, 200 µmol, 1.00 equiv.) and **1a** (171 mg, 400 µmol, 2.00 equiv.). Column chromatography (SiO_2/Cyclohexane:EtOAc 97:3 (V:V)) gave the product as a colourless solid (59.2 mg, 180 µmol, 90%)

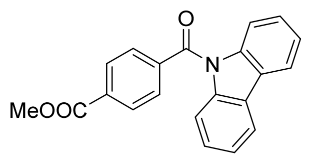

2i

^{1}H NMR (600 MHz, $CDCl_3$): δ = 8.23 (d, *J* = 8.5 Hz, 2H), 8.04 (ddd, *J* = 7.7, 1.4, 0.7 Hz, 2H), 7.81 (d, *J* = 8.4 Hz, 2H), 7.51 (d, *J* = 8.2 Hz, 2H), 7.39 (td, *J* = 7.5, 1.1 Hz, 2H), 7.34 (ddd, *J* = 8.5, 7.3, 1.4 Hz, 2H), 4.02 (s, 3H) ppm;**^{13}C NMR (151 MHz, $CDCl_3$):** δ = 168.8, 166.3, 139.9, 139.0, 133.5, 130.3, 129.0, 127.1, 126.4, 123.9, 120.1, 116.0, 52.7 ppm; **FTIR (ATR, neat, cm^{-1}):** $\tilde{\nu}$ = 3042 (w), 3003 (w), 2951 (w), 1719 (s), 1675 (s), 1610 (w), 1478 (w), 1452 (m), 1443 (m), 1331 (s); **MS (APCI):** m/z = 330.0 $[M+H]^+$; **Mp:** T = 119-121 °C; Analytical data are in accordance to the literature.[10]

(3-Bromophenyl)(9*H*-carbazol-9-yl)methanone (2j)

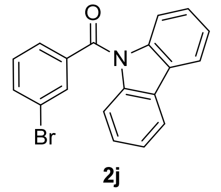

2j

Prepared according to **GP1** from 3-bromobenzamide (40.0 mg, 200 µmol, 1.00 equiv.) and **1a** (171 mg, 400 µmol, 2.00 equiv.). Column chromatography (SiO_2/Cyclohexane:EtOAc 97:3 (V:V)) gave the product as a colourless wax (58.6 mg, 167 µmol, 84%)

^{1}H NMR (600 MHz, $CDCl_3$): δ = 8.04 – 7.99 (m, 2H), 7.89 (t, *J* = 1.7 Hz, 1H), 7.78 (ddd, *J* = 8.1, 2.0, 1.1 Hz, 1H), 7.62 (ddd, *J* = 7.7, 1.6, 1.0 Hz, 1H), 7.52 (dt, *J* = 7.8, 0.8 Hz, 2H), 7.44 – 7.32 (m, 5H) ppm; **^{13}C NMR (151 MHz, $CDCl_3$):** δ =168.0, 139.1, 137.8, 135.5, 132.0, 130.6, 127.7, 127.1, 126.3, 123.9, 123.2, 120.1, 115.9 ppm; **FTIR (ATR, neat, cm^{-1}):** $\tilde{\nu}$ = 3046 (w), 3013 (w), 2962 (w), 2933 (w), 2868 (w), 1732 (m), 1678 (s), 1597 (w), 1566 (w), 1442 (s) 1416 (m); **HRMS (APCI):** *m/z* $[M]^{\bullet+}$ calcd. for: $C_{19}H_{12}BrNO^+$ m/z = 349.0097, found 349.0096; The analytical data are in accordane with the literature. [11]

(2-Bromophenyl)(9*H*-carbazol-9-yl)methanone (2k)

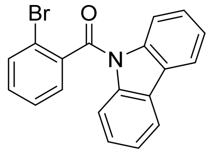

2k

Prepared according to **GP1** from 2-bromobenzamide (40.0 mg, 200 µmol, 1.00 equiv.) and **1a** (171 mg, 400 µmol, 2.00 equiv.). Column chromatography (SiO_2/Cyclohexane:EtOAc 97:3 (V:V)) gave the product as a colourless solid (38.8 mg, 111 µmol, 55%)

^{1}H NMR (600 MHz, $CDCl_3$): δ = 8.0 (d, *J* = 7.7 Hz, 2H), 7.7 (d, *J* = 8.0 Hz, 1H), 7.6 – 7.4 (m, 4H), 7.4 – 7.3 (m, 5H) ppm; **^{13}C NMR (151 MHz, $CDCl_3$):** δ = 167.4, 138.6, 138.6, 133.8, 132.1, 129.0, 128.4, 127.5, 126.8, 124.3, 119.9, 116.0 ppm; **FTIR (ATR, neat, cm^{-1}):** $\tilde{\nu}$ = 3061 (w), 2958 (w), 2922 (w), 2852 (w), 1674 (s), 1598 (w), 1587 (m), 1489 (w), 1467 (m), 1443 (s), 1359 (s); **HRMS (APCI): *m/z* [M+H]$^+$** calcd. for: $C_{19}H_{13}BrNO^+$ 350.0175, found 350.0169; Mp: 102-103 °C; The analytical data are in accordane with the literature. [11]

(3,5-Bis(trifluoromethyl)phenyl)(9*H*-carbazol-9-yl)methanone (2l)

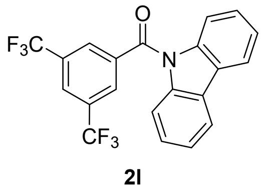

2l

Prepared according to **GP1** from 3,5-bis(trifluoromethyl)benzamide (51.4 mg, 200 µmol, 1.00 equiv.) and **1a** (171 mg, 400 µmol, 2.00 equiv.). Column chromatography (SiO_2/Cyclohexane:EtOAc 97:3 (V:V)) gave the product as a colourless solid (74.4 mg, 183 µmol, 91%)

^{1}H NMR (600 MHz, $CDCl_3$): δ = 8.19 (d, *J* = 1.6 Hz, 2H), 8.16 (s, 1H), 8.04 (dd, *J* = 7.6, 1.4 Hz, 2H), 7.45 (d, *J* = 8.2 Hz, 2H), 7.41 (td, *J* = 7.4, 1.1 Hz, 2H), 7.39 – 7.35 (m, 2H) ppm;**^{13}C NMR (151 MHz, $CDCl_3$):** δ = 166.3, 138.7, 137.7, 132.8 (q, *J* = 34.2 Hz), 129.6 (d, *J* = 4.0 Hz), 127.3, 124.5, 122.8 (q, *J* = 272.3, 271.6 Hz), 115.7 ppm; **^{19}F NMR (565 MHz, $CDCl_3$):** δ = -62.97 ppm; **FTIR (ATR, neat, cm^{-1}):** $\tilde{\nu}$ = 3052 (w), 2928 (w), 2854 (w), 2219 (w), 1686 (m), 1670 (m), 1616 (w), 1490 (w), 1453 (m), 1444 (m), 1275 (s); **HRMS (APCI):** *m/z* $[M]^{\bullet+}$: calcd. for $C_{21}H_{11}F_6NO^+$: 407.0739, found 407.0739; **Mp:** T = 143-145 °C;

(9*H*-Carbazol-9-yl)(3,5-dibromophenyl)methanone (2m)

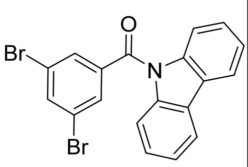

2m

Prepared according to **GP1** from 3,5-dibromobenzamide (55.8 mg, 200 µmol, 1.00 equiv.) and **1a** (171 mg, 400 µmol, 2.00 equiv.). Column chromatography (SiO_2/Cyclohexane:EtOAc 97:3 (V:V)) gave the product as a colourless solid (75.5 mg, 176 µmol, 88%)

^{1}H NMR (600 MHz, $CDCl_3$): δ = 8.07 – 8.02 (m, 2H), 7.97 (t, *J* = 1.8 Hz, 1H), 7.80 (d, *J* = 1.8 Hz, 2H), 7.60 – 7.51 (m, 2H), 7.41 (m, 4H) ppm.; **^{13}C NMR (151 MHz, $CDCl_3$):** δ = 166.4, 139.0, 138.8, 137.8, 130.7, 127.2, 126.4, 124.2, 123.7, 120.2, 115.9 ppm; **FTIR (ATR, neat, cm^{-1}):** $\tilde{\nu}$ = 3068 (w), 2962 (w), 2829 (w), 1665 (s), 1556 (m), 1478 (w), 1453 (m), 1442 (m), 1320 (s), 1291 (s); **HRMS (APCI):** *m/z* $[M]^{\bullet+}$: calcd. for $C_{19}H_{11}Br_2NO^{\bullet+}$: 426.9202, found 426.9196; **Mp:** T = 146-148 °C;

(9*H*-Carbazol-9-yl)(3,5-dimethoxyphenyl)methanone (2n)

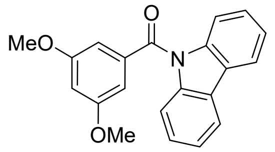

2n

Prepared according to **GP1** from 3,5-dimethoxybenzamide (36.2 mg, 200 µmol, 1.00 equiv.) and **1a** (171 mg, 400 µmol, 2.00 equiv.). Column chromatography (SiO_2/Cyclohexane:EtOAc 97:3 (V:V)) gave the product as a colourless solid (36.9 mg, 111 µmol, 56%)

^{1}H NMR (600 MHz, $CDCl_3$): δ = 8.03 – 7.95 (m, 2H), 7.64 – 7.52 (m, 2H), 7.45 – 7.30 (m, 4H), 6.83 (d, *J* = 2.3 Hz, 2H), 6.72 (t, *J* = 2.3 Hz, 1H), 3.79 (s, 6H) ppm; **^{13}C NMR (151 MHz, $CDCl_3$):** δ = 169.5, 161.3, 139.2, 137.8, 127.0, 126.2, 123.7, 119.9, 116.1, 106.6, 104.9, 55.8 ppm; **FTIR (ATR, neat, cm^{-1}):** $\tilde{\nu}$ = 3096 (w); 3056 (w), 3008 (w), 2960 (w), 2845 (w), 2229 (w), 1669 (s), 1489 (w), 1443 (s), 1424 (s), 1361 (m); **HRMS (APCI):** *m/z* $[M+H]^+$ calcd. for $C_{21}H_{18}NO_3^+$: 332.1281, found 332.1276; **Mp:** T = 113-114 °C;

(9*H*-Carbazol-9-yl)(mesityl)methanone (2o)

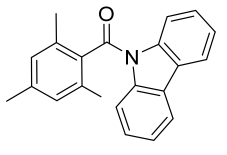

2o

Prepared according to **GP1** from 2,4,6-trimethylbenzamide (32.6 mg, 200 µmol, 1.00 equiv.) and **1a** (171 mg, 400 µmol, 2.00 equiv.). Column chromatography (SiO_2/Cyclohexane:EtOAc 97:3 (V:V)) gave the product as a colourless solid (57.0 mg, 182 µmol, 91%)

^{1}H NMR (600 MHz, $CDCl_3$): δ = 8.97 – 8.87 (m, 1H), 8.06 – 7.93 (m, 2H), 7.63 – 7.43 (m, 2H), 7.32 – 7.03 (m, 2H), 7.00 (s, 2H), 6.18 – 6.06 (m, 1H), 2.41 (s, 3H), 2.15 (s, 6H)., aromatic ^{1}H-signals were broadened;**^{13}C NMR (151 MHz, $CDCl_3$):** δ = 170.4, 140.0, 139.1/138.0 (two signals caused by rotamers), 134.7, 134.5, 129.2, 128.0/127.0(two signals caused by rotamers), 126.9/126.5 (two signals caused by rotamers), 124.6/123.6 (two signals, caused by rotamers), 120.1/119.5 (two signals, caused by rotamers), 118.2/113.8 (two signals, caused by rotamers), 21.5, 19.1 ppm doublets are caused by rotational barrier of amide; **FTIR (ATR, neat, cm^{-1}):** $\tilde{\nu}$ = 3066 (w), 2948 (w), 2916 (w), 1693 (s), 1609 (w), 1490 (w), 1476 (w), 1441 (m), 1426 (m), 1328 (s), 1301 (s); **HRMS (APCI):** *m/z* $[M+H]^+$ calcd. for: $C_{22}H_{20}NO^+$ 314.1539, found 314.1536; **Mp:** T = 205-206 °C

(9*H*-Carbazol-9-yl)(3,4,5-trimethoxyphenyl)methanone (2p)

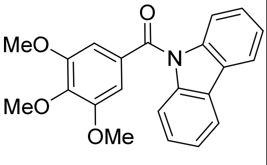

2p

Prepared according to **GP1** from 3,4,5-trimethoxybenzamide (42.2 mg, 200 µmol, 1.00 equiv.) and **1a** (171 mg, 400 µmol, 2.00 equiv.). Column chromatography (SiO_2/Cyclohexane:EtOAc 97:3 (V:V)) gave the product as a colourless solid (49.6 mg, 137 µmol, 69%)

^{1}H NMR (600 MHz, $CDCl_3$): δ = 8.11 – 7.96 (m, 2H), 7.65 – 7.51 (m, 2H), 7.47 – 7.32 (m, 4H), 6.98 (s, 2H), 3.97 (s, 3H), 3.80 (s, 6H) ppm;**^{13}C NMR (151 MHz, $CDCl_3$):** δ = 169.3, 153.6, 141.8, 139.3, 130.6, 126.9, 126.1, 123.6, 120.0, 116.0 106.7, 61.3, 56.5 ppm; **FTIR (ATR, neat, cm^{-1}):** $\tilde{\nu}$ = 3061 (w), 3005 (w), 2935 (w), 2835 (w), 1732 (w), 1670 (s), 1584 (s), 1477 (w), 1443 (s), 1413 (m), 1357 (m), 1325 (s), **HRMS (APCI):** *m/z* $[M+H]^+$ calcd. for $C_{22}H_{20}NO_4^+$: 362.1387, found 362.1383; HRMS (APCI): *m/z* $[M+Na]^+$ calcd. for $C_{22}H_{19}NO_4Na$ $[M+Na]^+$ 384.1206, found 384.1204; **Mp:**T = 122-124 °C;

Ethyl 9*H*-carbazole-9-carboxylate (2q)

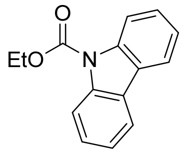

2q

Prepared according to **GP1** from urethane (17.8 mg, 200 µmol, 1.00 equiv.) and **1a** (171 mg, 400 µmol, 2.00 equiv.). Column chromatography (SiO_2/Cyclohexane:EtOAc 97:3 (V:V)) gave the product as a colourless solid (35.5 mg, 148 µmol, 74%)

^{1}H NMR (600 MHz, $CDCl_3$): δ = 8.33 (d, *J* = 8.4 Hz, 2H), 7.99 (ddd, *J* = 7.7, 1.4, 0.7 Hz, 2H), 7.49 (ddd, *J* = 8.4, 7.3, 1.3 Hz, 2H), 7.38 (td, *J* = 7.5, 1.0 Hz, 2H), 4.61 (q, *J* = 7.2 Hz, 2H), 1.58 (t, *J* = 7.1 Hz, 3H) ppm;**^{13}C NMR (151 MHz, $CDCl_3$):** δ = 152.6, 138.4, 127.3, 126.0, 123.4, 120.0, 116.4, 63.2, 14.6 ppm; **FTIR (ATR, neat, cm^{-1}):** $\tilde{\nu}$ = 3058 (w), 3044 (w), 3012 (w), 2981 (w), 2934 (w), 2903 (w), 2867 (w), 1938 (w), 1903 (w), 1732 (s), 1597 (w), 1476 (w), 1443 (s), 1370 (m); **HRMS (APCI):** *m/z* $[M]^{\bullet+}$ calcd. for: $C_{15}H_{13}NO_2^+$ 239.0941, found 239.0939; Mp: 70-71 °C; The analytical data is in accordance with the literature [12]

1-(9*H*-Carbazol-9-yl)-2-phenylethan-1-one (2r)

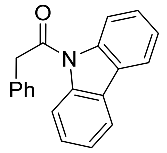

2r

Prepared according to **GP1** from 2-phenylacetamide (27.0 mg, 200 µmol, 1.00 equiv.) and **1a** (171 mg, 400 µmol, 2.00 equiv.). Column chromatography (SiO_2/Cyclohexane:EtOAc 97:3 (V:V)) gave the product as a colourless solid (46.6 mg, 163 µmol, 82%)

^{1}H NMR (600 MHz, $CDCl_3$): δ = 8.26 (d, *J* = 8.3 Hz, 2H), 8.05 – 8.00 (m, 2H), 7.48 (ddd, *J* = 8.5, 7.2, 1.4 Hz, 2H), 7.41 (td, *J* = 7.5, 0.9 Hz, 4H), 7.38 – 7.32 (m, 4H), 4.52 (s, 2H) ppm; **^{13}C NMR (151 MHz, $CDCl_3$):** δ = 171.3, 138.7, 133.7, 129.7, 129.0, 127.6, 127.5, 126.7, 124.0, 120.0, 116.7, 45.5 ppm; **FTIR (ATR, neat, cm^{-1}):** $\tilde{\nu}$ = 3063 (w), 3028 (w), 3006 (w), 2962 (w), 2925 (w), 1694 (s), 1615 (w), 1599 (m), 1586 (w), 1486 (m), 1476 (m), 1443 (s), 1420 (s); **HRMS (APCI):** *m/z* $[M+H]^+$ calcd. for: $C_{20}H_{16}NO^+$ 286.1226, found 286.1224; **Mp:** T = 138-140 °C; The analytical data is in accordance with the literature [13]

9-(2,4,6-Trimethylbenzoyl)-9*H*-carbazole-2-carbonitrile (2s)

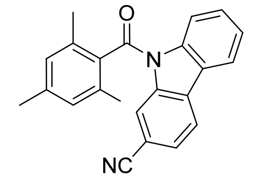

2s

Prepared according to **GP1** from 2,4,6-trimethylbenzamide (32.6 mg, 200 µmol, 1.00 equiv.) and **1s** (181 mg, 400 µmol, 2.00 equiv.). Column chromatography (SiO_2/Cyclohexane:EtOAc 97:3 (V:V)) gave the product as a colourless solid (41.0 mg, 121 µmol, 61%).

^{1}H NMR (600 MHz, $CDCl_3$): δ = 9.63 – 8.69 (m, rotamers, 1H), 8.20 – 7.91 (m, 2H), 7.85 – 7.29 (m, 3H), 7.02 (s, 2H), 6.21 (br, rotamers, 1H), 2.42 (s, 3H), 2.13 (s, 6H) ppm aromatic ^{1}H-signals were broadened;**^{13}C NMR (151 MHz, $CDCl_3$):** δ = Aromatic carbon were not identifiable due to rotation 21.4, 19.0 ppm; **FTIR (ATR, neat, cm^{-1}):** $\tilde{\nu}$ = 3052 (w), 2225 (m), 1937 (w), 1921 (w), 1786 (w), 1633 (w), 1535 (m), 1452 (s), 1416 (m), 1375 (m); **HRMS (EI):** *m/z* $[M]^{•+}$ calcd. for $C_{23}H_{18}N_2O^+$: 338.1419, found 338.1418; **Mp:** T = 192-194 °C;

6-Bromo-9-(2,4,6-trimethylbenzoyl)-9*H*-carbazole-2-carbonitrile (2t)

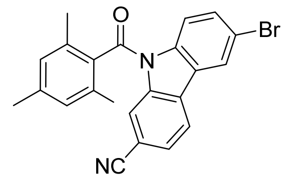

2t

Prepared according to **GP1** from 2,4,6-trimethylbenzamide (32.6 mg, 200 µmol, 1.00 equiv.) and **1t** (213 mg, 400 µmol, 2.00 equiv.). Column chromatography (SiO_2/Cyclohexane:EtOAc 97:3 (V:V)) gave the product as a colourless solid (39.2 mg, 93.9 µmol, 47%)

^{1}H NMR (600 MHz, $CDCl_3$): δ 9.06 (br, rotamers, 1H), 8.37 – 7.97 (m, 2H), 7.68 (br. rotamers, 1H), 7.42 – 7.17 (m, 1H), 7.05 (s, 2H), 6.14 (br, rotamers 1H), 2.44 (s, 3H), 2.14 (s, 6H) ppm aromatic ^{1}H-signals were broadened;**^{13}C NMR (151 MHz, $CDCl_3$):** δ =13C NMR (151 MHz, Chloroform-d) δ = 170.1, 140.9, 138.5, 137.7, 134.5, 133.4, 132.6, 131.9, 129.5, 128.1, 127.1, 123.8, 123.4, 122.3, 121.0, 120.5, 119.7, 119.2, 118.1, 117.6, 115.5, 111.5, 21.5, 19.0 ppm (aromatic signals were separated by rotational barriers); **FTIR (ATR, neat, cm^{-1}):** $\tilde{\nu}$ = 2918 (w), 2853 (w), 2218 (m), 1682 (s), 1610 (m), 1577 (w), 1482 (s), 1454 (m), 1426 (s), 1413 (s); **HRMS (EI):** *m/z* $[M]^{•+}$ calcd. for $C_{22}H_{17}N_2BrO^+$: 416.0524, found 416.0513; **Mp:** T = 227-230 °C;

Acridin-10(9*H*)-yl(mesityl)methanone (3a)

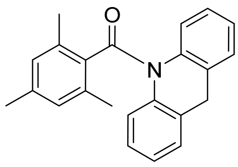

3a

Prepared according to **GP1** from 2,4,6-trimethylbenzamide (32.6 mg, 200 µmol, 1.00 equiv.) and 10*H*-dibenzo[*b,e*]iodinin-5-ium trifluoromethanesulfonate (176 mg, 400 µmol, 2.00 equiv.). Column chromatography (SiO_2/Cyclohexane:EtOAc 97:3 (V:V)) gave the product as a colorless solid (31.0 mg, 94.7 µmol, 47%)

^{1}H NMR (600 MHz, $CDCl_3$): δ = 7.9 (s, 1H), 7.4 – 7.3 (m, 1H), 7.1 (d, J = 114.1 Hz, 4H), 6.7 (d, J = 110.2 Hz, 4H), 3.9 (s, 2H), 2.2 (s, 9H) ppm;**^{13}C NMR (151 MHz, $CDCl_3$):** δ = 169.4, 138.3, 134.0, 133.8, 133.5, 132.6, 130.2, 129.8, 129.6, 128.2, 127.2, 126.2, 125.6, 123.8, 34.0, 26.9, 20.2 ppm; **FTIR (ATR, neat, cm^{-1}):** $\tilde{\nu}$ = 2948 (w), 2917 (w), 2862 (w), 2818 (w), 1655 (s), 1610 (m), 1473 (s), 1327 (s), 1269 (s), 1074 (m); **HRMS**

(APCI): *m/z* $[M+H]^+$ calcd. for $C_{23}H_{21}NO^+$: 328.1696, found 328.1693; **Mp:** T = 149-152 °C

Mesityl(10*H*-phenoxazin-10-yl)methanone (3b)

Prepared according to **GP1** with varied temperature at 135 C from 2,4,6-trimethylbenzamide (16.3 mg, 100 µmol, 1.00 equiv.) and Dibenzo[*b,e*][1,4]iodaoxin-5-ium trifluoromethansulfonate (88.8 mg, 200 µmol, 2.00 equiv.). Column chromatography (SiO_2/PE:EtOAc 20:1 (V:V)) gave the product as a colourless solid (27.6 mg, 83.8 µmol, 84%)

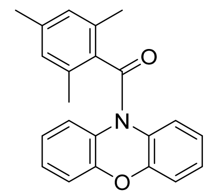

3b

This substrate required low-temperature NMR measurement to yield any meaningful data.

^{1}H NMR (600 MHz, $CDOD_3$): δ = 7.89 (dd, *J* = 8.0, 1.6 Hz, 1H), 7.32 (td, *J* = 7.8, 1.6 Hz, 1H), 7.27 – 7.22 (m, 2H), 7.15 – 7.10 (m, 2H), 6.84 (q, *J* = 0.7 Hz, 2H), 6.72 (ddd, *J* = 8.5, 6.7, 2.2 Hz, 1H), 6.50 – 6.47 (m, 1H), 2.26 (s, 3H), 2.20 (s, 6H) ppm;**^{13}C NMR (151 MHz, $CDOD_3$):** δ = 170.7, 150.8, 150.8, 139.2, 134.2, 132.6, 128.4, 127.9, 127.8, 127.2, 127.0, 125.8, 123.6, 123.1, 122.9, 116.4, 116.3, 19.8, 17.8 ppm; **FTIR (ATR, neat, cm^{-1}):** $\tilde{\nu}$ = 2918, 1660, 1587, 1480, 1322, 1266, 1072, 860, 761, 748; **HRMS (ESI):** *m/z* [M+H] cald. for: $C_{22}H_{20}NO_2^+$: 330.1489, found 330.1488; **Mp:** T = 141-143 °C

This compound was published synthesized with the identical procedure in earlier work and is given as a reference point towards other compunds **3**. [14]

Mesityl(2-methyl-10*H*-phenoxazin-10-yl)methanone (3c)

Prepared according to **GP1** with varied temperature at 135 °C from 2,4,6-trimethylbenzamide (32.6 mg, 200 µmol, 1.00 equiv.) and 3-methyldibenzo[b,e][1,4]iodaoxin-5-ium trifluoromethanesulfonate (183 mg, 400 µmol, 2.00 equiv.). Column chromatography (SiO_2/PE:EtOAc 20:1 (V:V)) gave the product as a colourless solid (50.1 mg, 146 µmol, 73%)

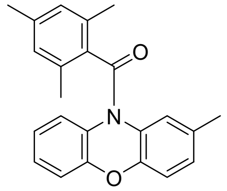

3c

NMR spectroscopy gave no meaningful data at room temperature due strong broadening of signals. Measuring at lower temperature gave no meaningful signals due to diminished solubility; **^{1}H NMR (600 MHz, $CDCl_3$)** δ: 8.03 – 7.66 (m, 1H), 7.24 – 5.73 (m, 9H), 2.25 (s, 3H), 2.22 (s, 6H), 2.44 – 1.76 (m, 3H) ppm; **^{13}C NMR (151 MHz, Chloroform-*d*):** δ = 169.9, 151.1, 148.7, 138.8, 134.6, 133.2, 128.2, 127.6, 127.1 – 125.8 (rotamers), 124.4, 123.8, 123.2, 122.8, 116.7, 116.3, 29.8, 21.2, 19.2 ppm; **FTIR (ATR, neat, cm^{-1}):** $\tilde{\nu}$ = 2918, 1664, 1611, 1480, 1322, 1264, 1069, 856, 755; **HRMS (ESI):** *m/z* $[M+Na]^+$: calcd. for $C_{23}H_{21}NNaO_2^+$: 366.1463, found 366.1465; **Mp:** T = 175-177 °C

(2-Bromo-10*H*-phenoxazin-10-yl)(mesityl)methanone (3d)

Prepared according to **GP1** with varied temperature at 135 °C from 2,4,6-trimethylbenzamide (32.6 mg, 200 µmol, 1.00 equiv.) and 3-bromodibenzo[*b,e*][1,4]iodaoxin-5-ium trifluoromethanesulfonate (209 mg, 400 µmol, 2.00 equiv.). Column chromatography (SiO_2/PE:EtOAc 20:1 (V:V)) gave the product as a colourless solid (20.1 mg, 49.2 µmol, 25%)

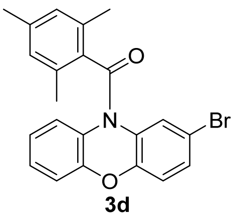

3d

NMR spectroscopy gave no meaningful data at room temperature due strong broadening of signals. Measuring at lower temperature gave no meaningful signals due to diminished solubility; **^{1}H NMR (600 MHz, $CDCl_3$):** δ = 8.20 – 7.81 (m, 1H), 7.17 – 6.26 (m, 8H), 2.26 (s, 3H), 2.21 (s, 9H) ppm; **^{13}C NMR (151 MHz, $CDCl_3$):** δ = 21.3, 19.2 ppm Aromatic signals gave no useful data due to broadening; **FTIR (ATR, neat, cm^{-1}):** $\tilde{\nu}$ = 2916, 1669, 1610, 1475, 1317, 1201, 1065, 850, 820, 754; **HRMS (ESI):** *m/z* [M+H]$^+$ calcd. for $C_{22}H_{19}BrNO_2^+$: 408.0594, found 408.0592; **Mp:** T = 177-179 °C;

10-(2,4,6-Trimethylbenzoyl)-10*H*-phenoxazine-2-carbonitrile (3e)

Prepared according to **GP1** with varied temperature at 135 °C from 2,4,6-trimethylbenzamide (32.6 mg, 200 µmol, 1.00 equiv.) and 3-cyanodibenzo[b,e][1,4]iodaoxin-5-ium trifluoromethanesulfonate (209 mg, 400 µmol, 2.00 equiv.). Column chromatography (SiO_2/PE:EtOAc 20:1 (V:V)) gave the product as a colourless solid (18.3 mg, 51.6 µmol, 26%)

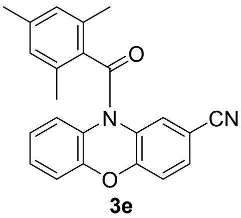

3e

NMR spectroscopy gave no meaningful data at room temperature due strong broadening of signals. Measuring at lower temperature gave no meaningful signals due to diminished solubility; **^{1}H NMR (600 MHz, $CDCl_3$):** δ = 8.58 – 7.66 (m, 1H), 7.21 – 6.20 (m, 8H), 2.27 (s, 3H), 2.20 (s, 6H) ppm; **^{13}C NMR (151 MHz, $CDCl_3$):** δ = 169.7, 154.1, 149.6, 134.5, 128.4, 117.7, 116.8, 21.2, 19.1 ppm, Phenoxazine signals were broadened and split due to rotamers; **IR (ATR, neat, cm^{-1}):** $\tilde{\nu}$ = 2920, 2228, 1673, 1611, 1479,1296, 1265, 1065, 852, 751; **HR-MS (ESI):** *m/z* [M+Na] calcd. For: $C_{23}H_{18}N_2NaO_2^+$: 377.1261, found 377.1261; **Mp:** T = 58-59 °C

(6*H*-Imidazo[4,5,1-de]12henazine-6-yl)(phenyl)methanone (3f)

Prepared according to **GP1** from Benzamide (24.2 mg, 200µmol, 1.00 equiv.) and 2,10b-diazaaceanthrylen-6-ium trifluoromethanesulfonate (187 mg, 400 µmol, 2.00 equiv.). Column chromatography (SiO_2/Cyclohexane:EtOAc 97:3 (V:V)) gave the product as a yellow solid (4.8 mg, 15.4 µmol, 8%) Single crystals were obtained *via* slow evaporation of a solution in EtOAc

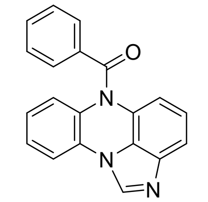

3f

^{1}H NMR (600 MHz, $CDCl_3$): δ = 8.47 (s, 1H), 7.73 (d, *J* = 7.8 Hz, 1H), 7.61 (d, *J* = 7.3 Hz, 2H), 7.48 (d, *J* = 8.2 Hz, 1H), 7.47 – 7.40 (m, 2H), 7.33 (t, *J* = 7.6 Hz, 2H), 7.21 (t, *J* = 7.9 Hz, 1H), 7.13 (t, *J* = 7.2 Hz, 1H), 6.90 – 6.81 (m, 2H) ppm;**^{13}C NMR (151 MHz, $CDCl_3$):** δ = 169.9, 169.4, 135.0, 133.4, 132.1, 131.8, 130.4, 129.6, 128.8, 128.6, 127.5, 126.4, 126.3, 125.6, 125.2, 116.2, 115.0,

112.8 ppm; **FTIR (ATR, neat, cm^{-1}):** $\tilde{\nu}$ = 2918 (w), 2853 (w), 2218 (m), 1682 (s), 1610 (m), 1577 (w), 1482 (s), 1454 (m), 1426 (s), 1413 (s); HR-MS (ESI): *m/z* [M+H]$^+$ calcd. for $C_{20}H_{14}N_3O^+$: 312.1131, found 312.1130; **Mp:** T = 227-230 °C;

NMR-Spectra

2-Bromo-7-cyanodibenzo[*b,d*]iodol-5-ium trifluoromethanesulfonate (1t)

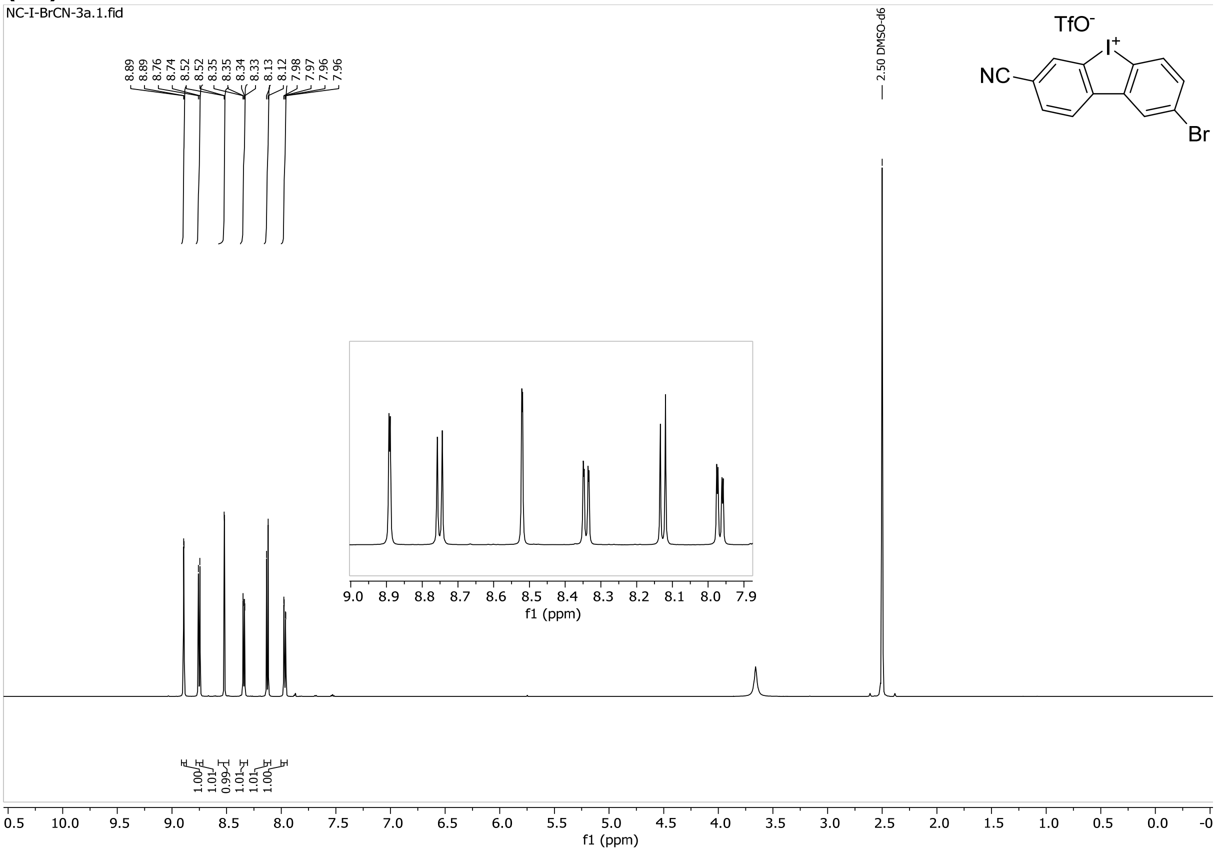

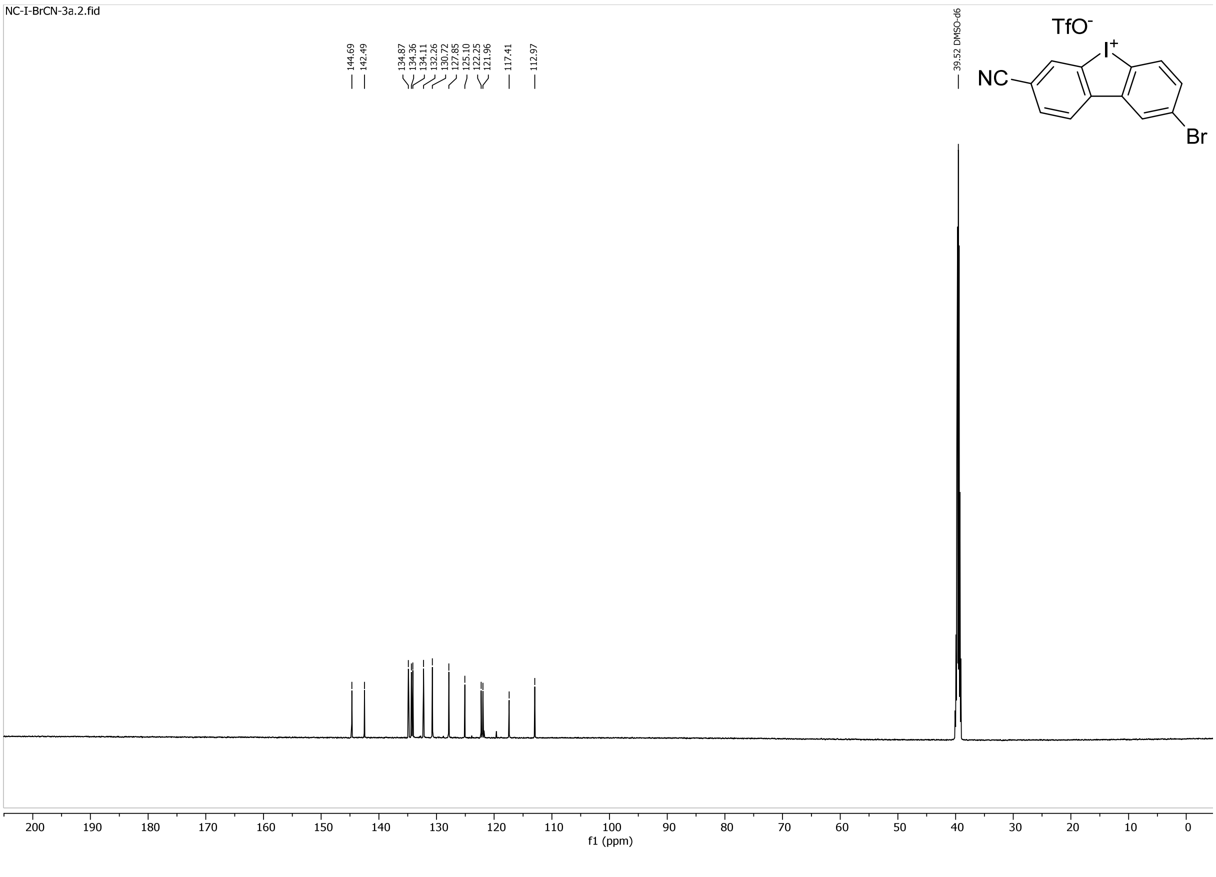

(4-Bromophenyl)(9*H*-carbazol-9-yl)methanone (2e)

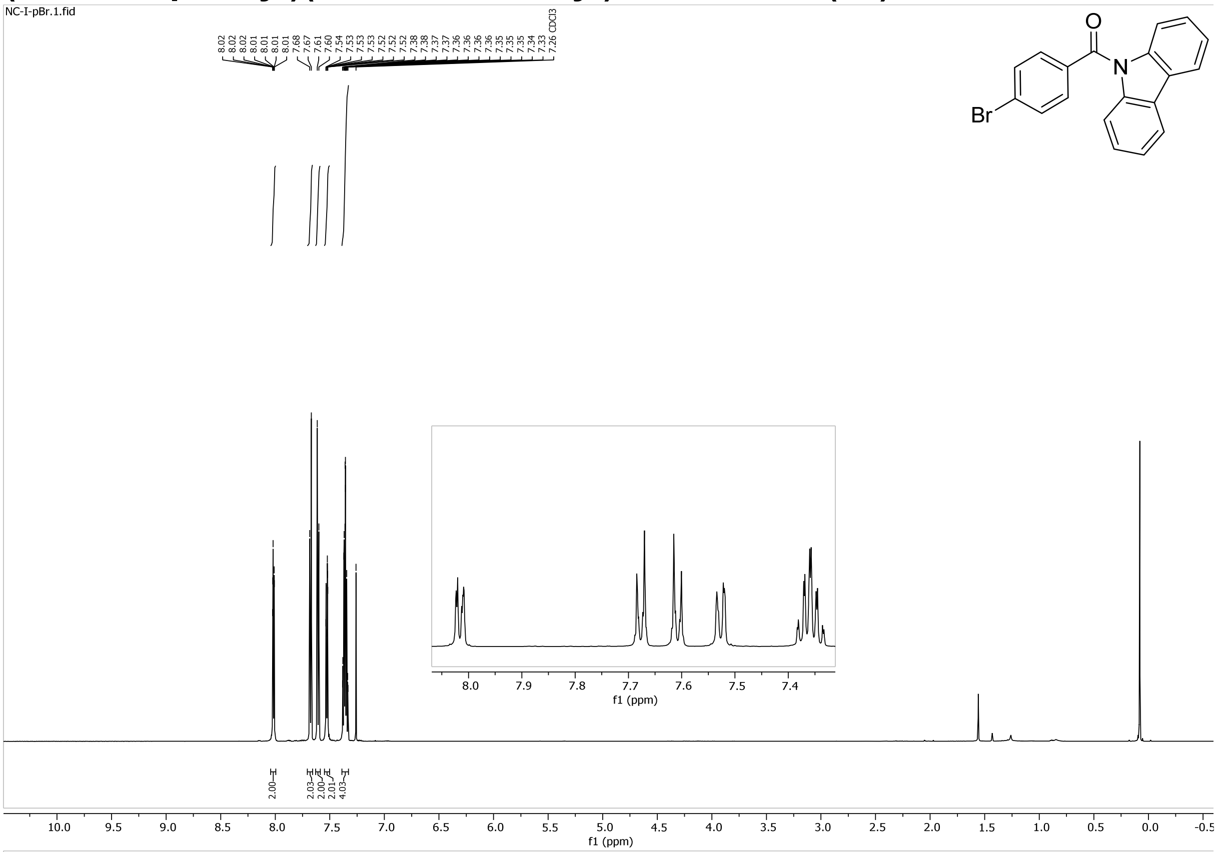

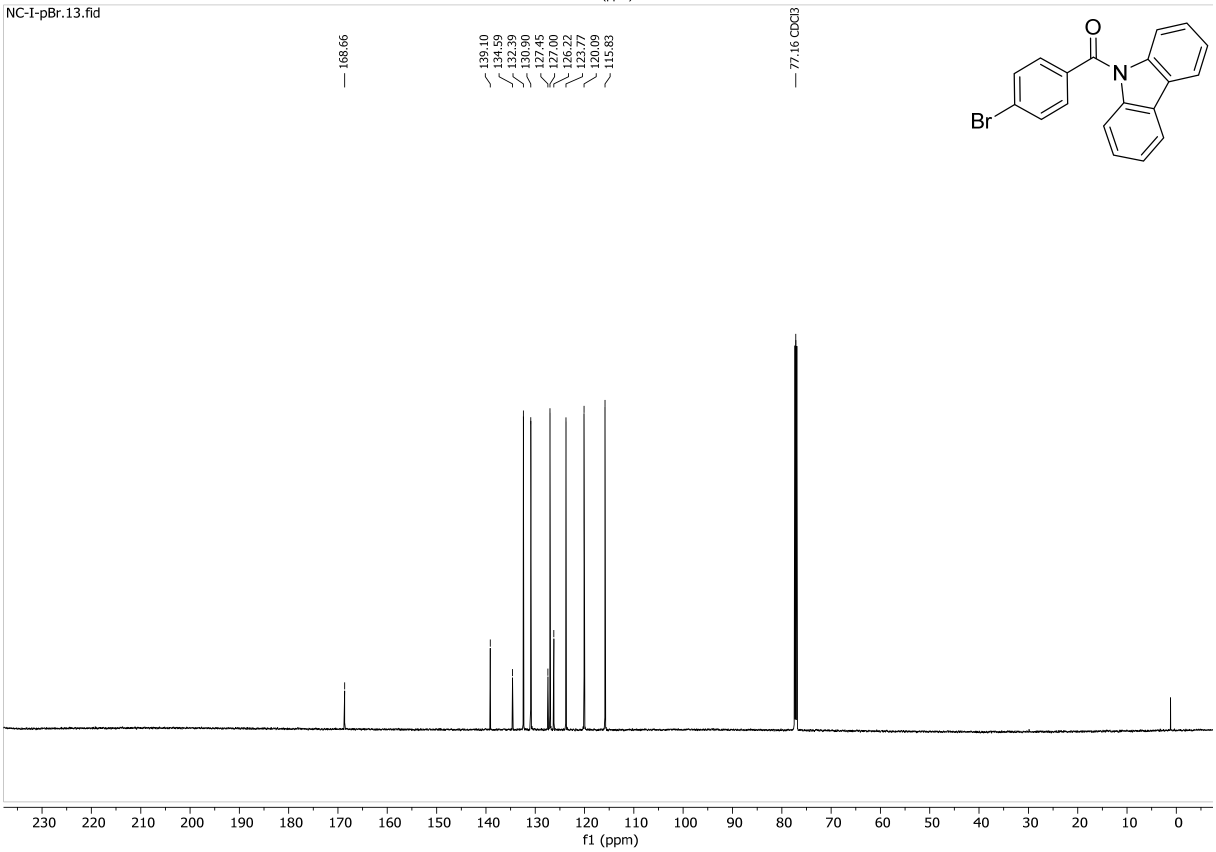

(9*H*-Carbazol-9-yl)(4-nitrophenyl)methanone(2g)

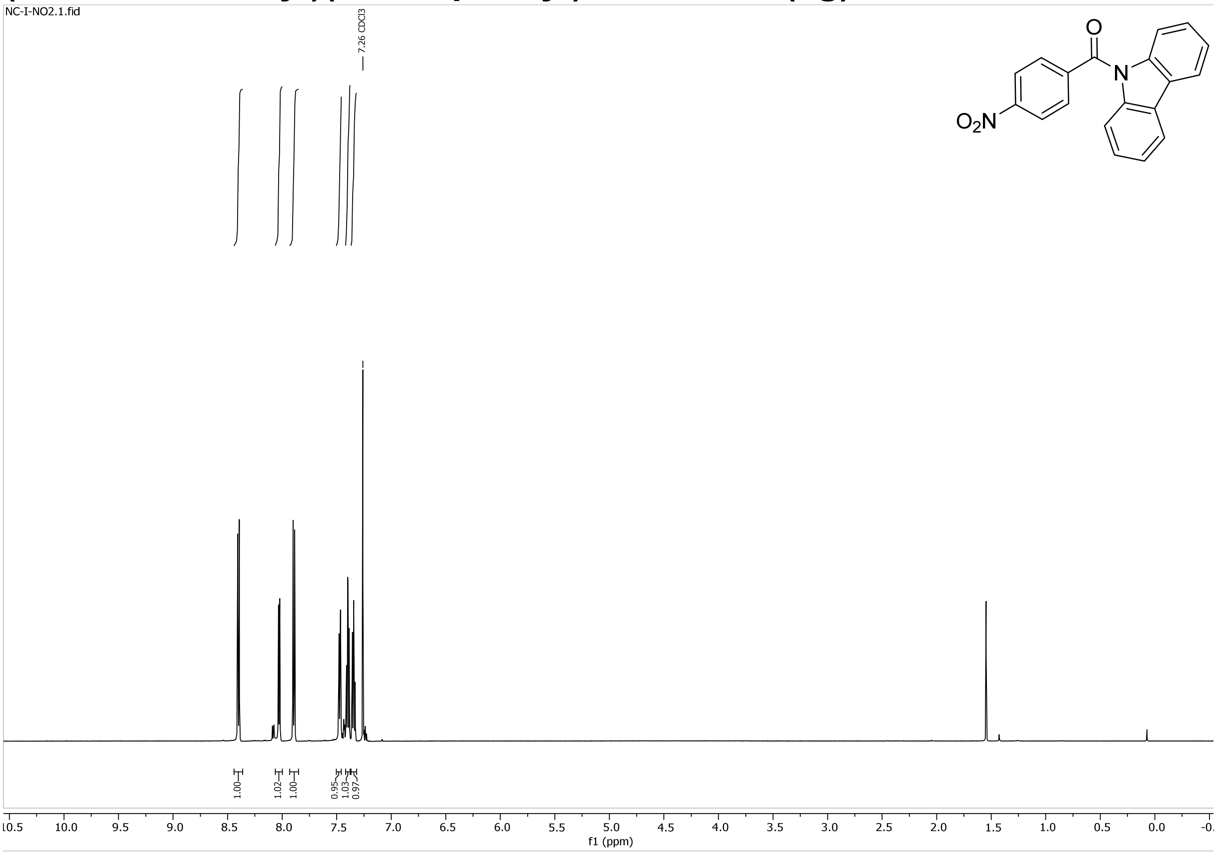

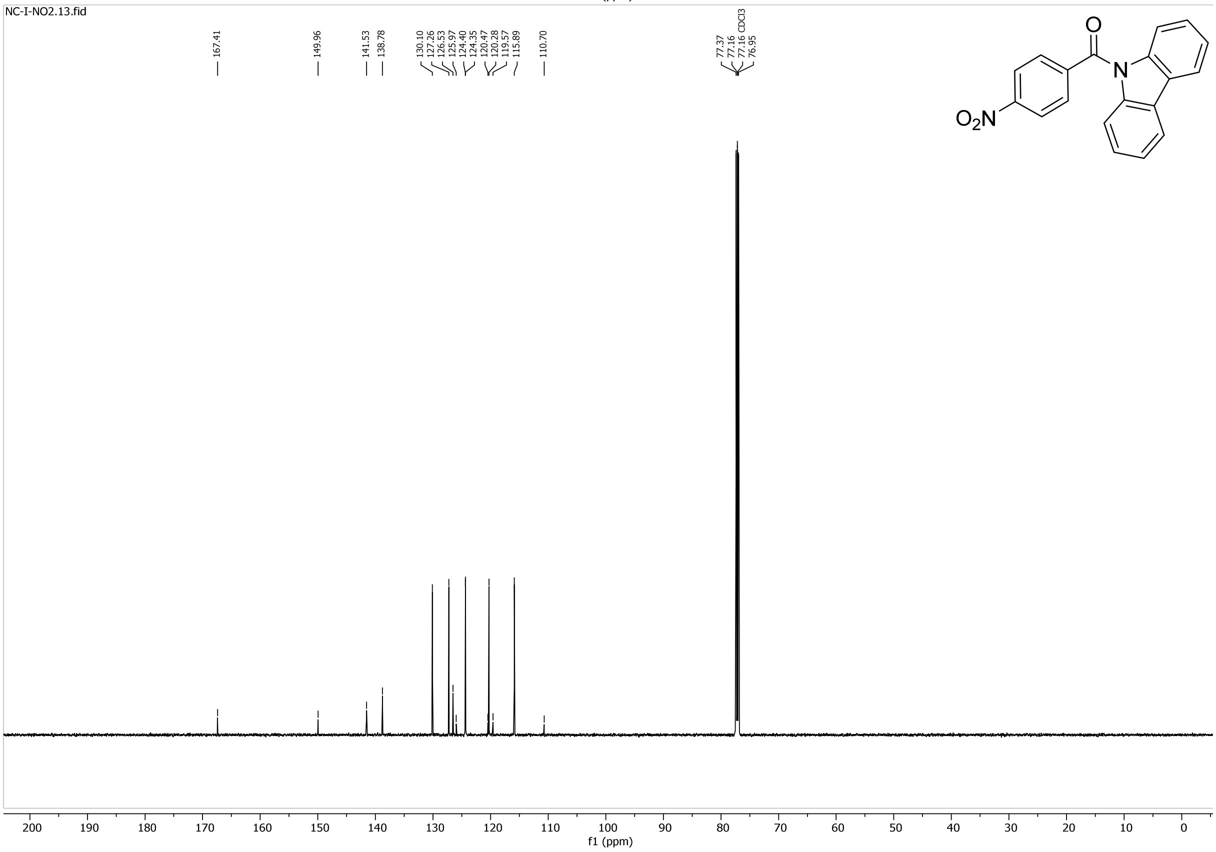

Methyl 4-(9*H*-carbazole-9-carbonyl)benzoate (2i)

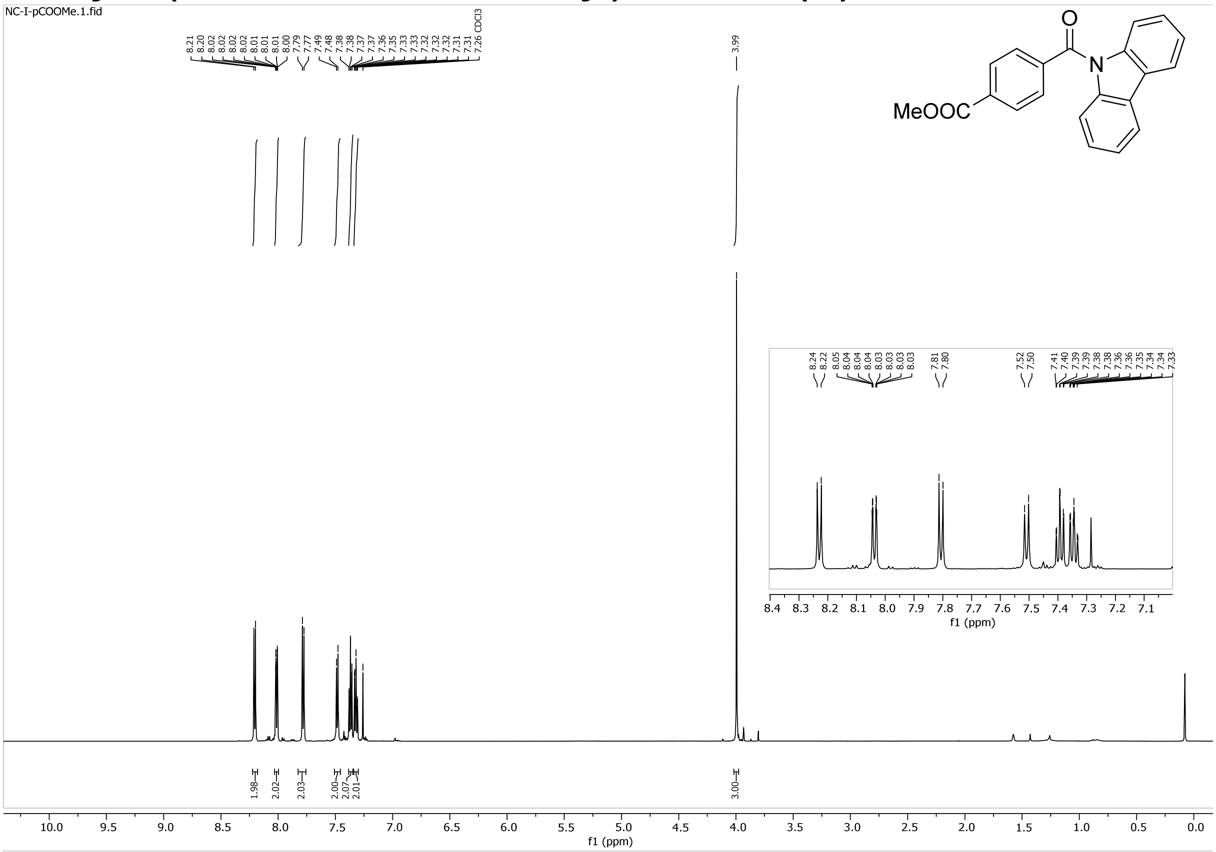

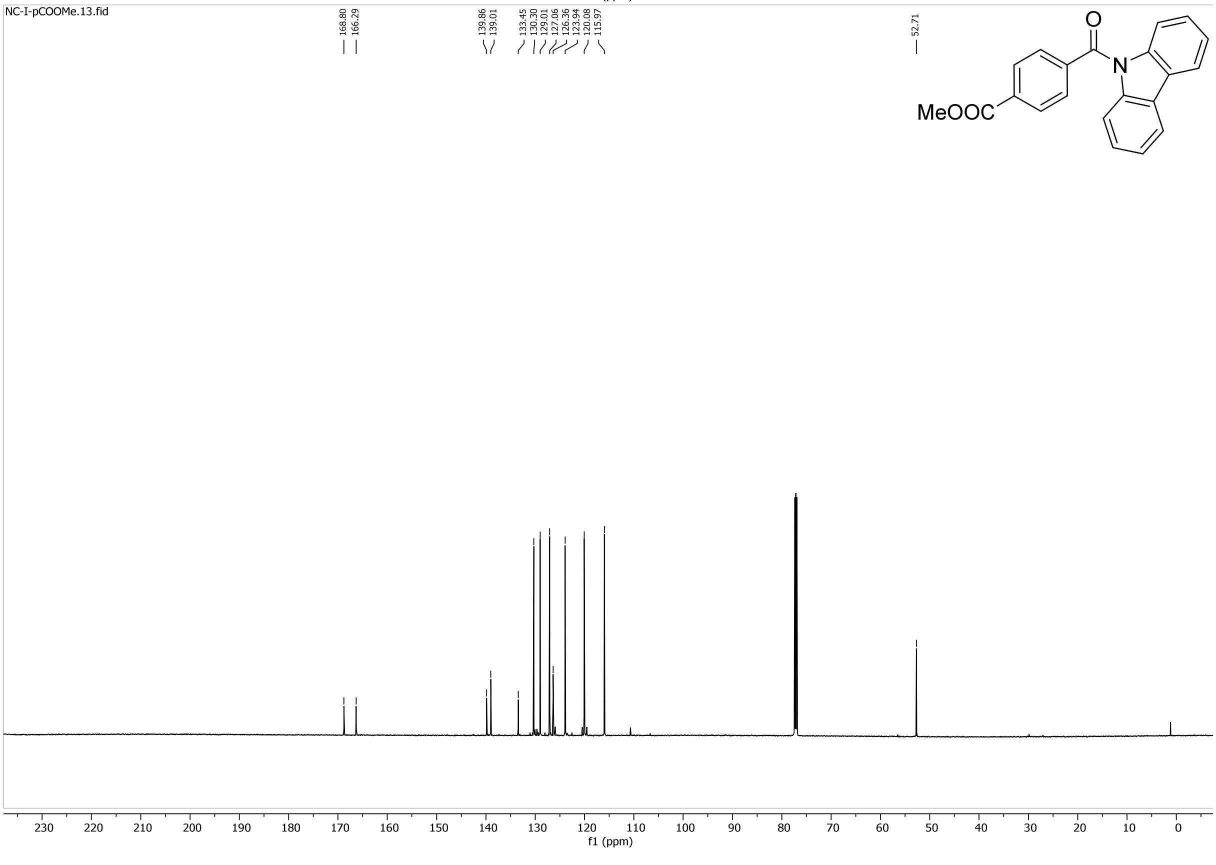

(3-Bromophenyl)(9*H*-carbazol-9-yl)methanone (2j)

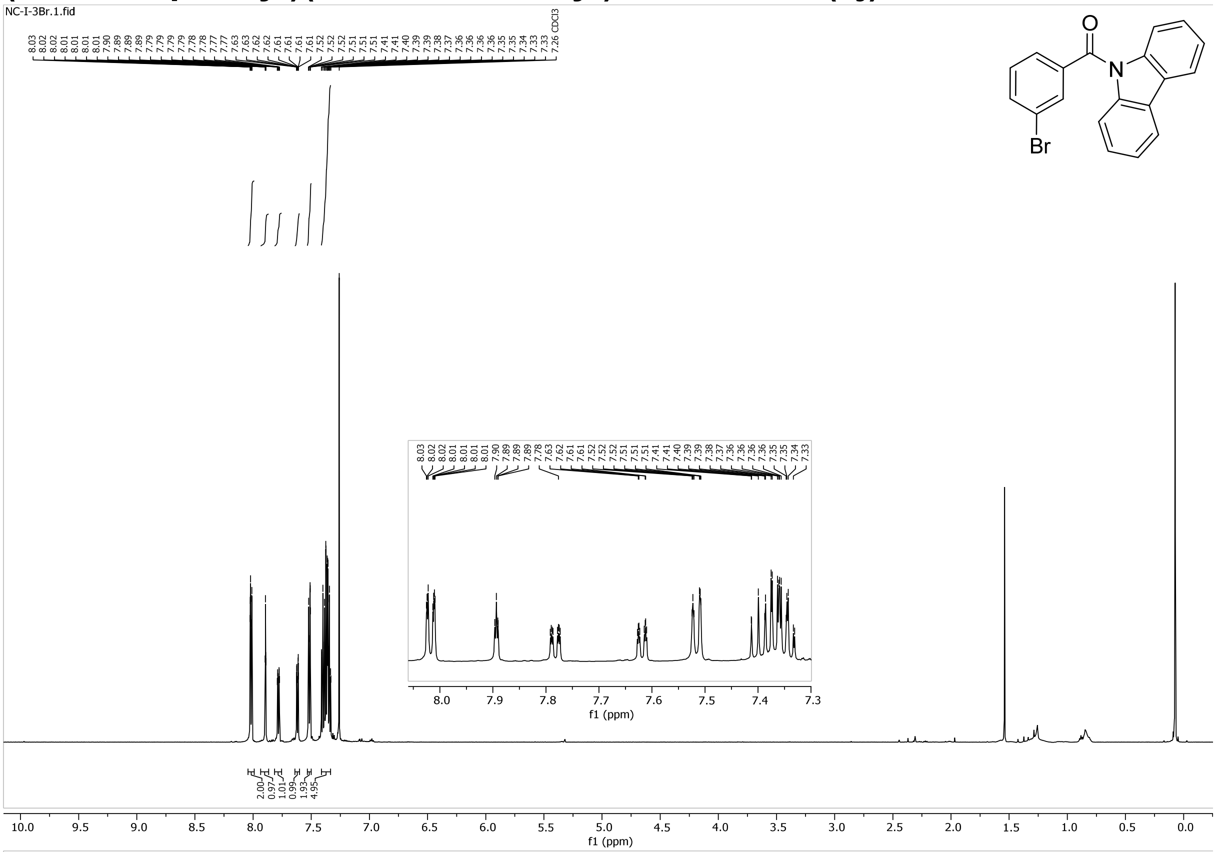

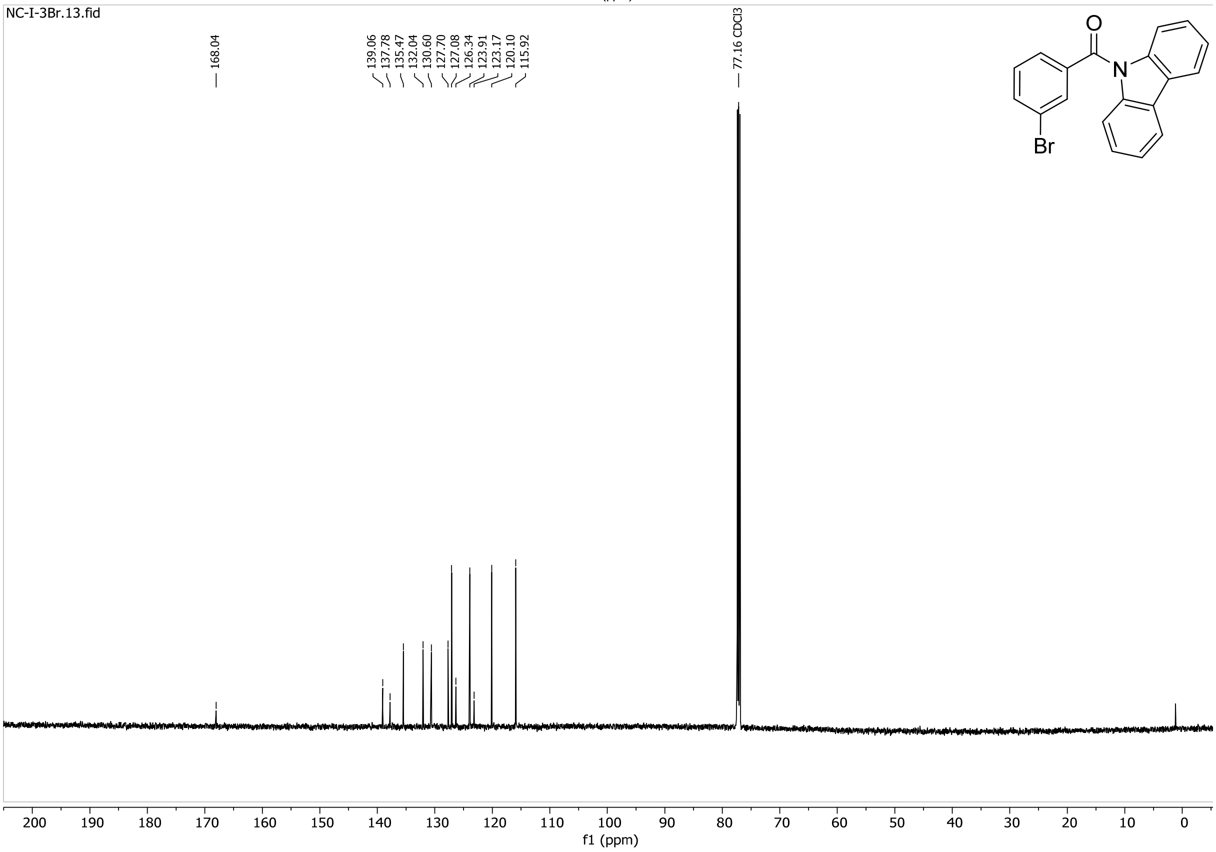

(2-Bromophenyl)(9*H*-carbazol-9-yl)methanone (2k)

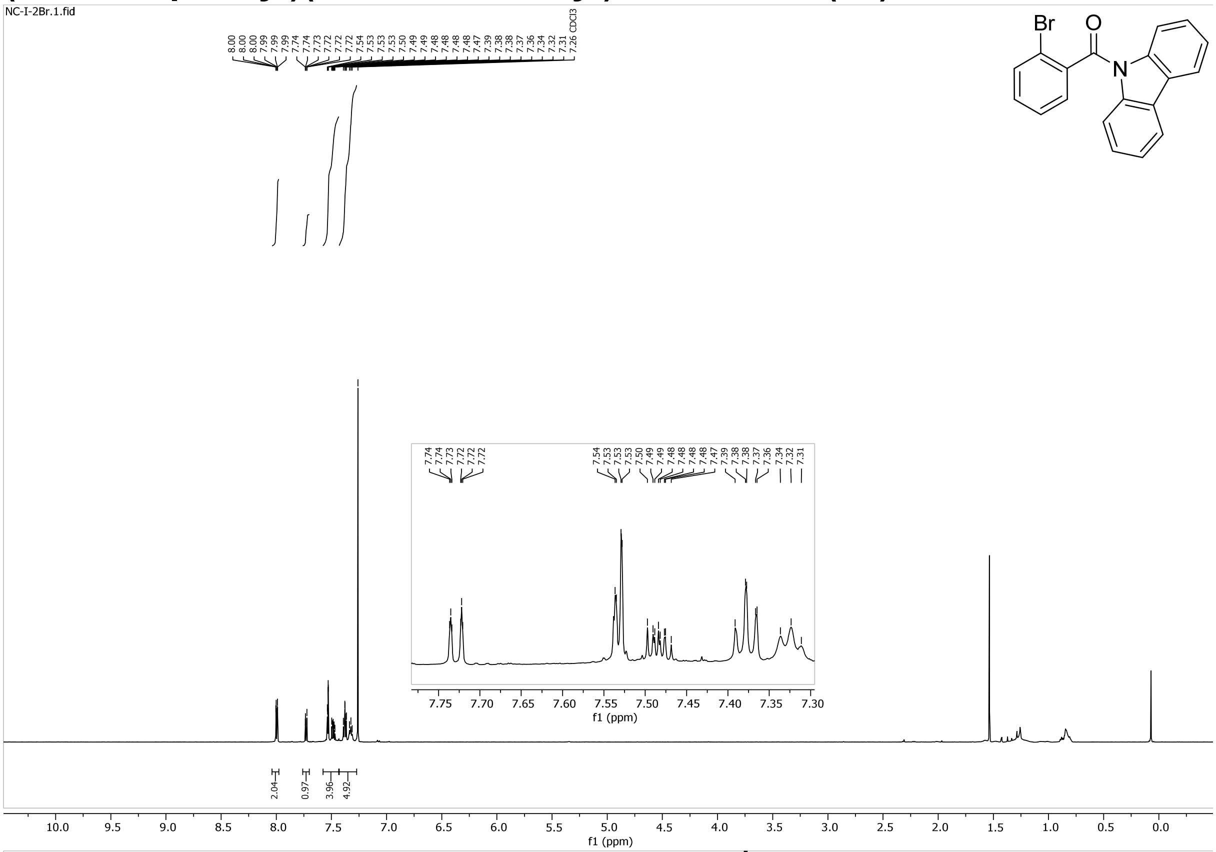

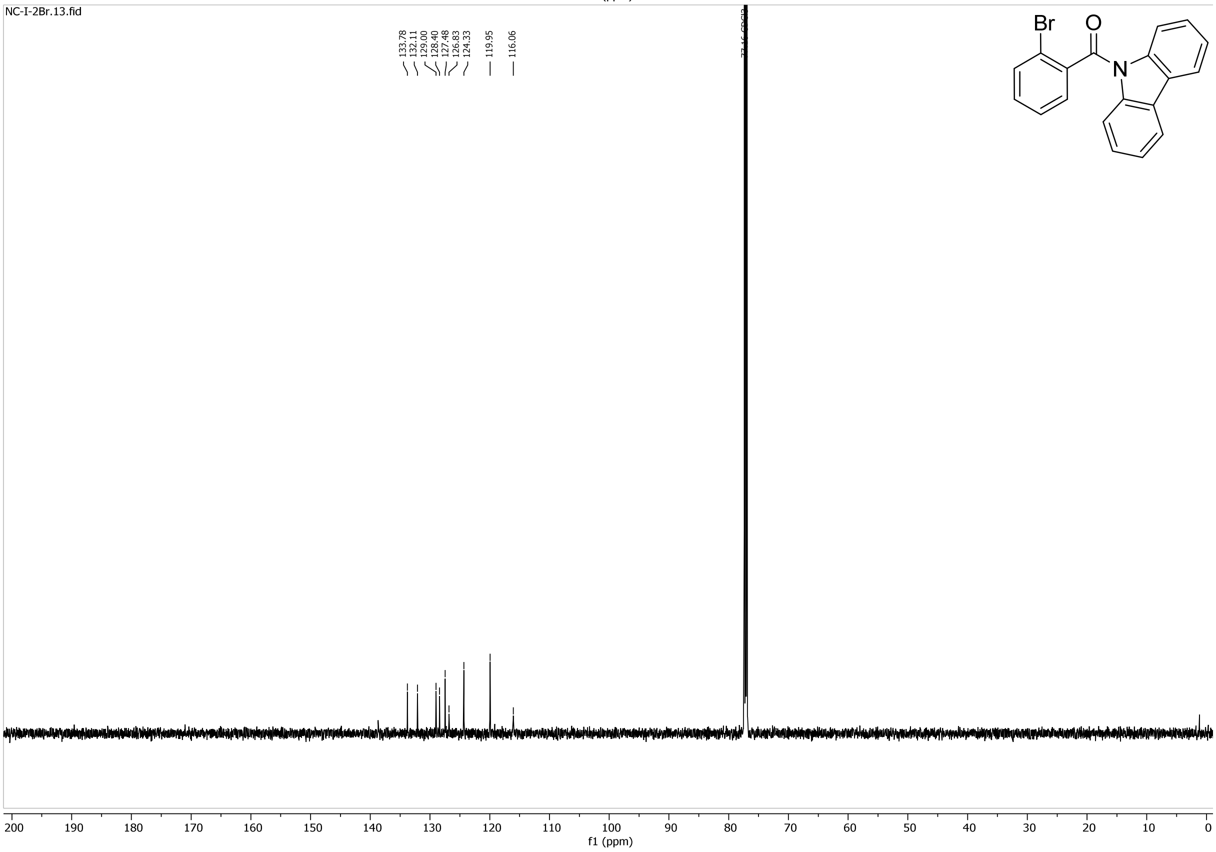

3,5-Bis(trifluoromethyl)phenyl)(9*H*-carbazol-9-yl)methanone (2l)

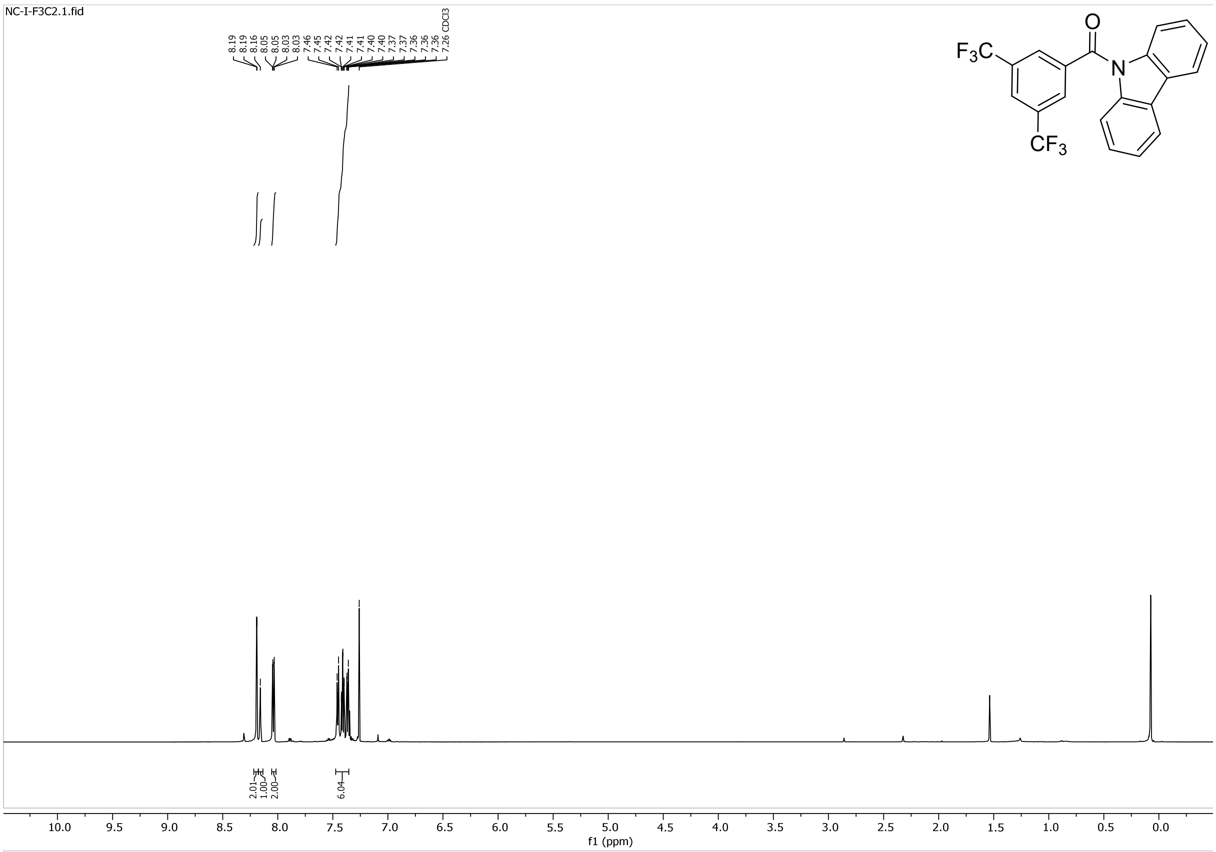

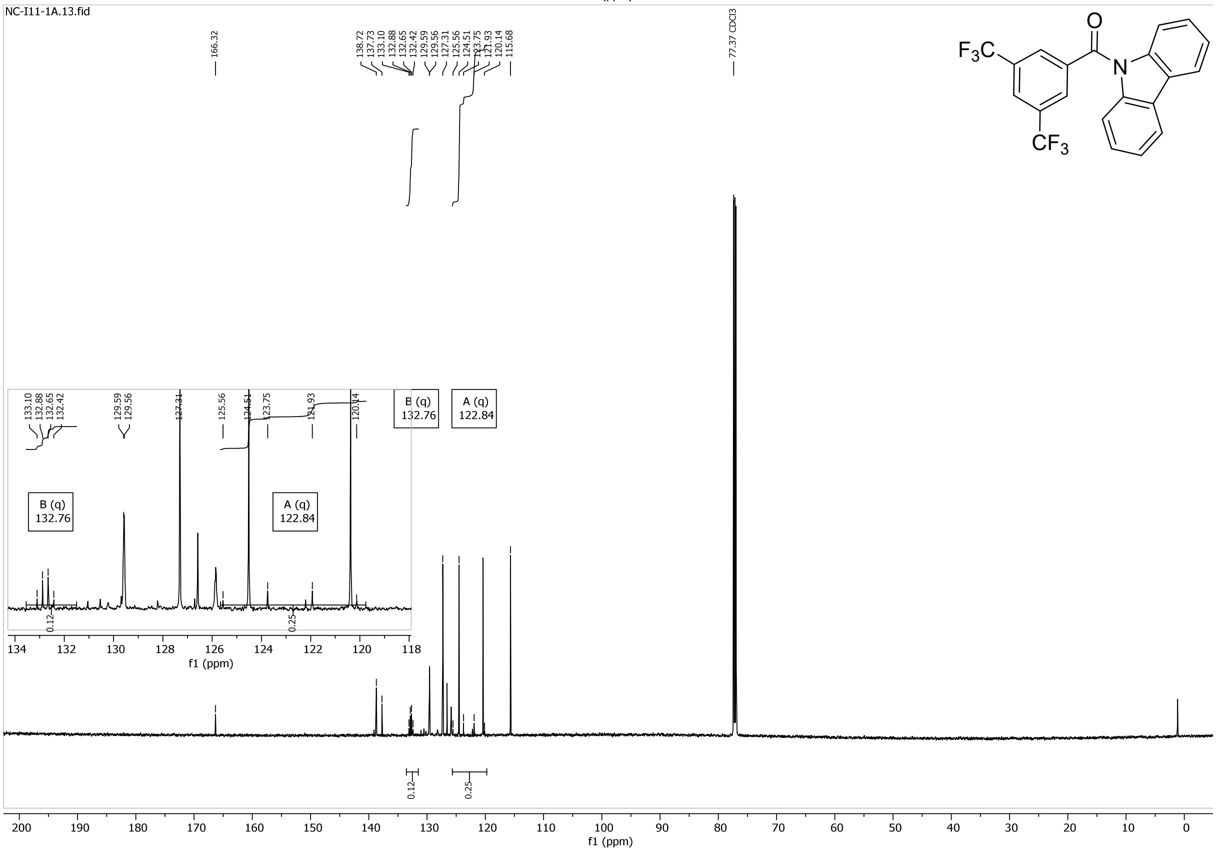

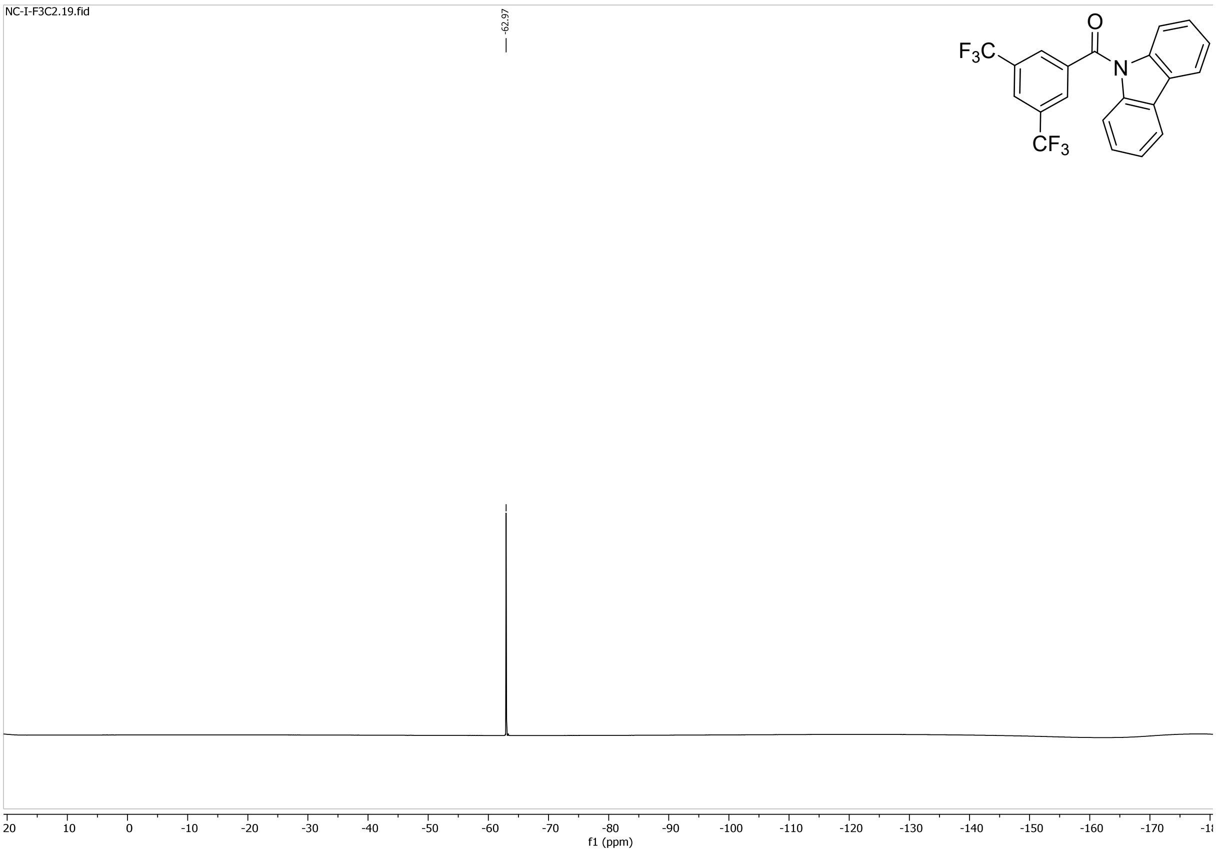

NC-I-F3C2.19.fid
-62.97
F3C
CF3
O
N
f1 (ppm)

(9*H*-Carbazol-9-yl)(3,5-dibromophenyl)methanone (2m)

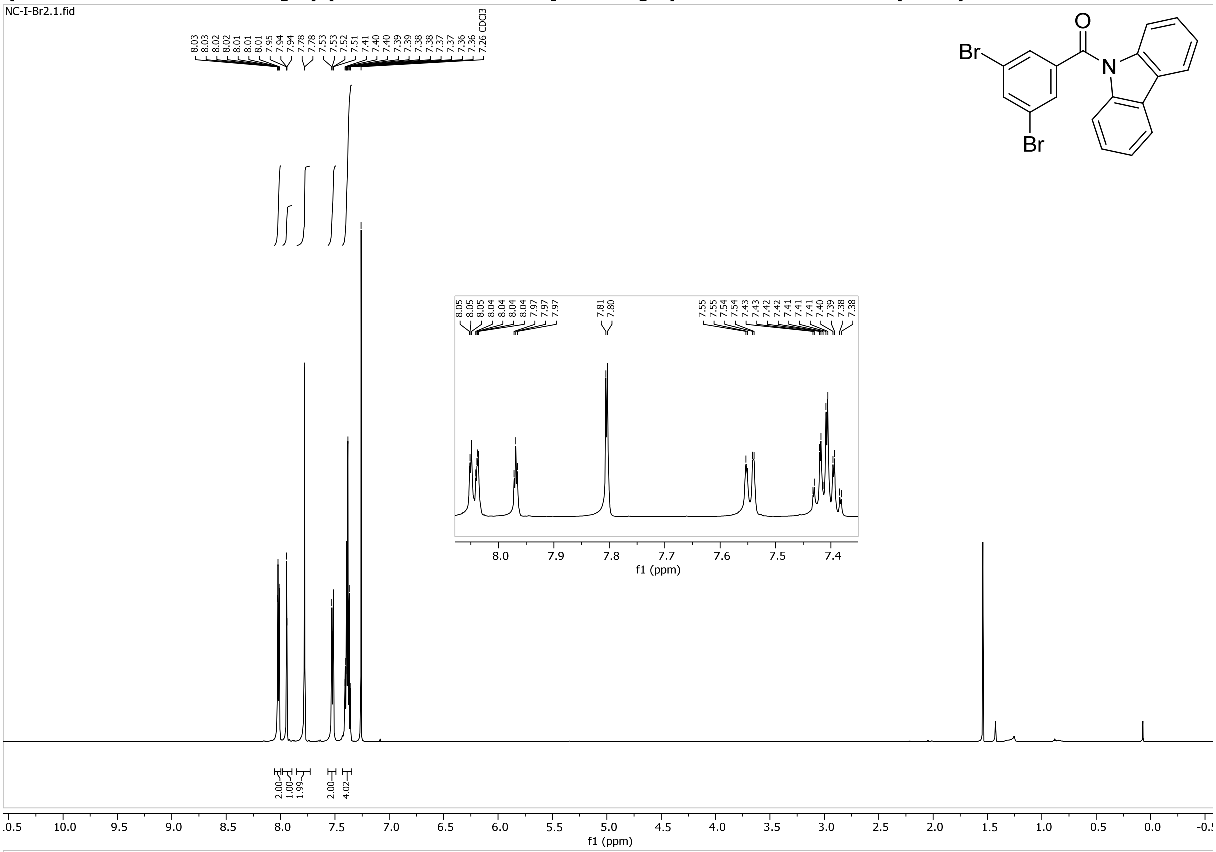

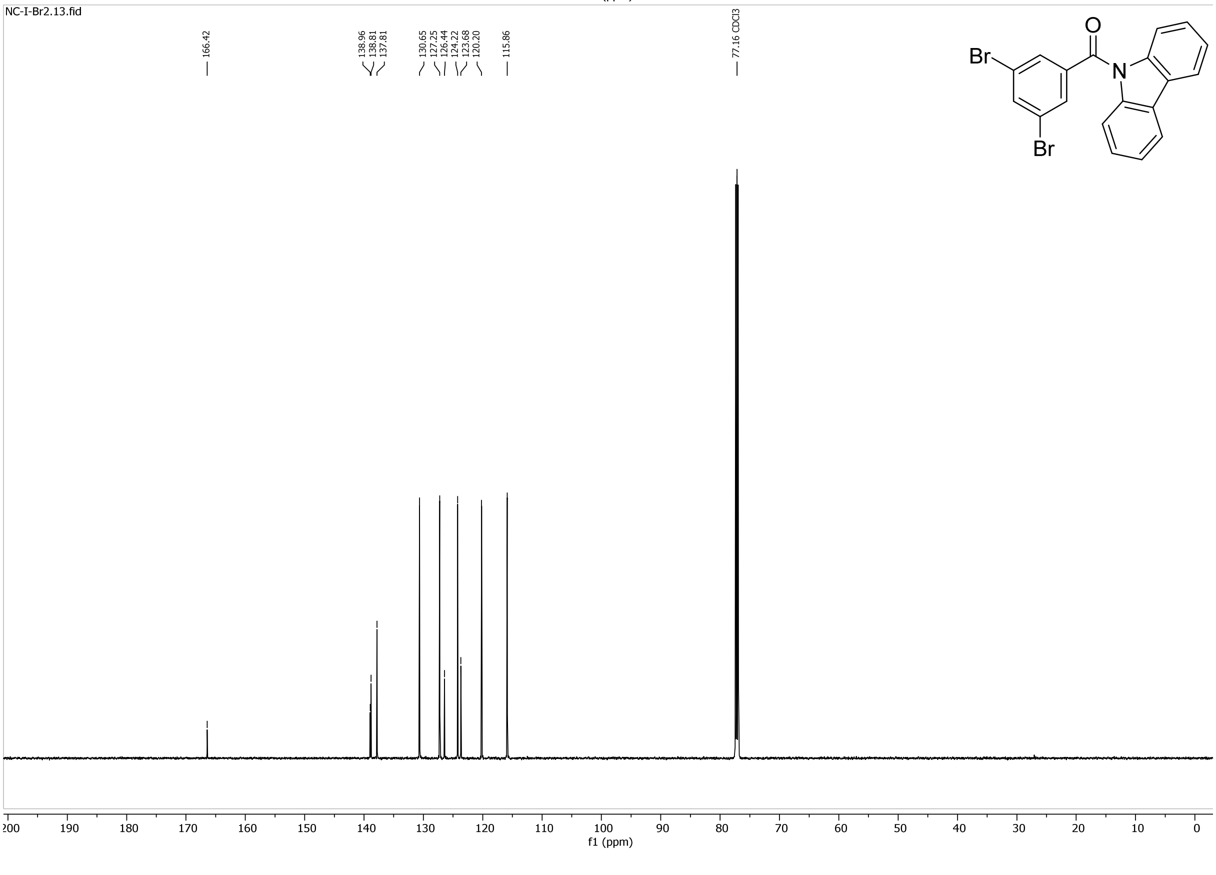

(9*H*-Carbazol-9-yl)(3,5-dimethoxyphenyl)methanone(2n)

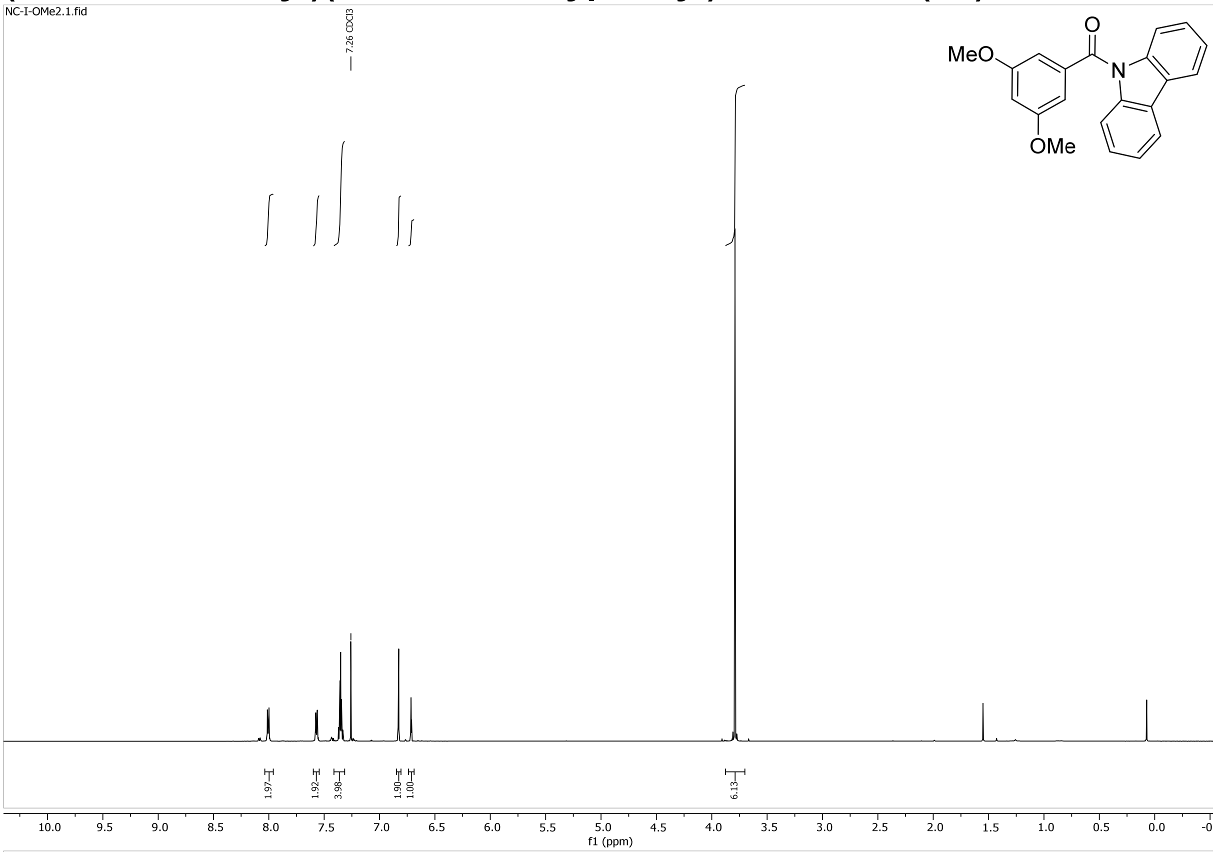

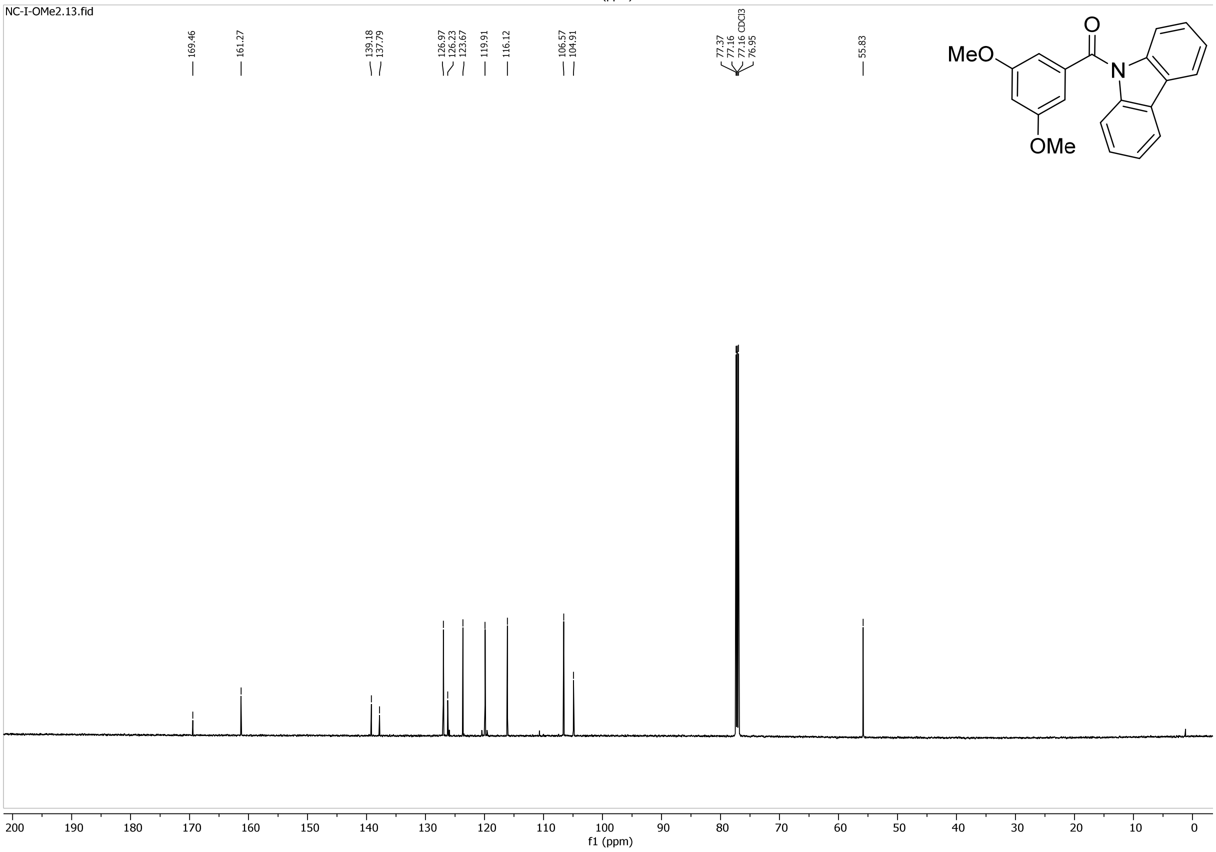

(9*H*-Carbazol-9-yl)(mesityl)methanone (2o)

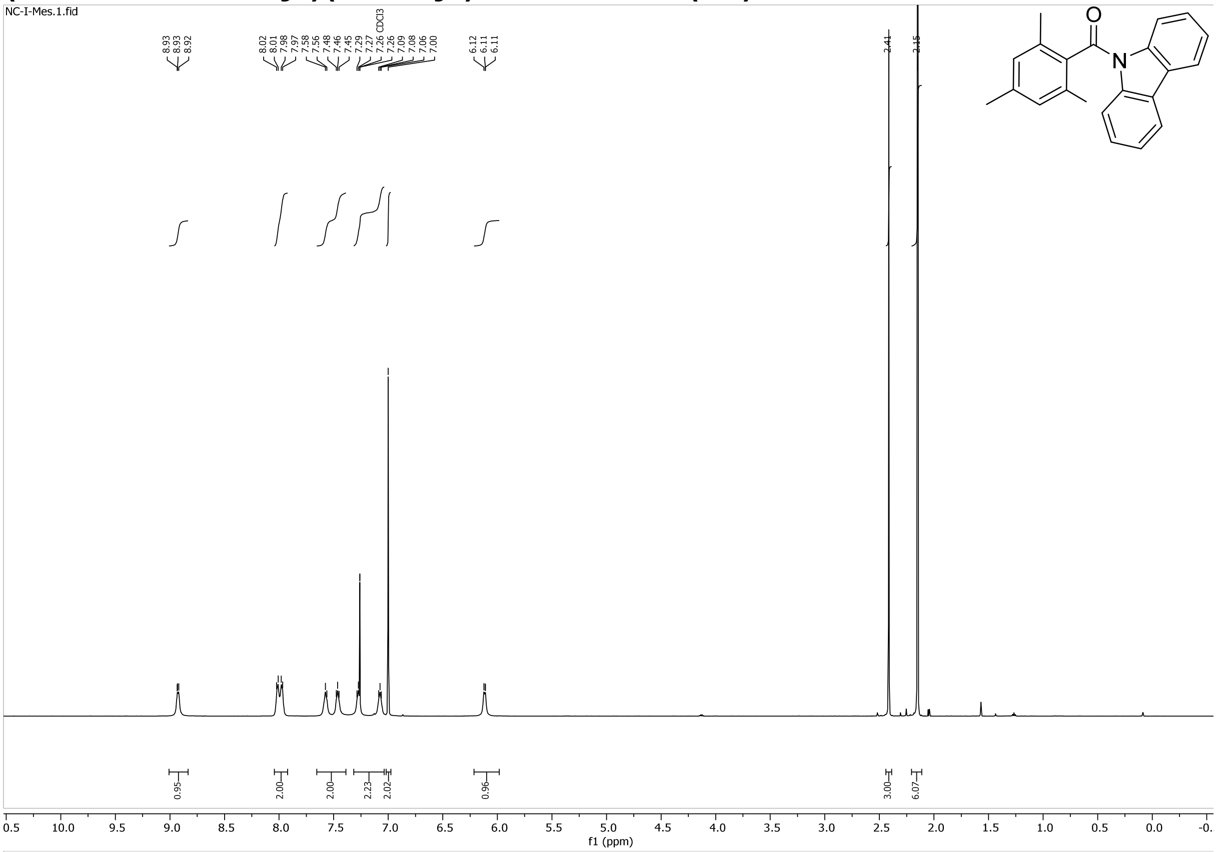

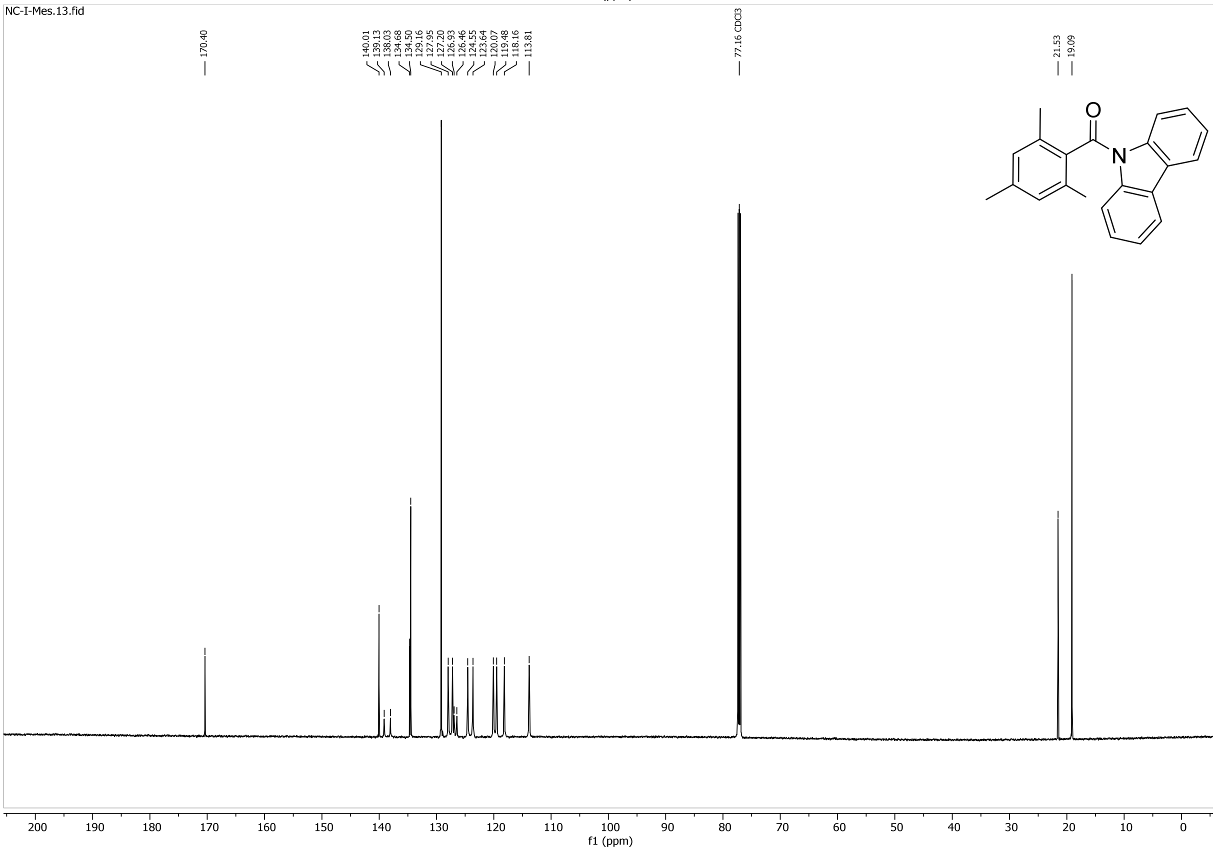

(9*H*-Carbazol-9-yl)(3,4,5-trimethoxyphenyl)methanone (2p)

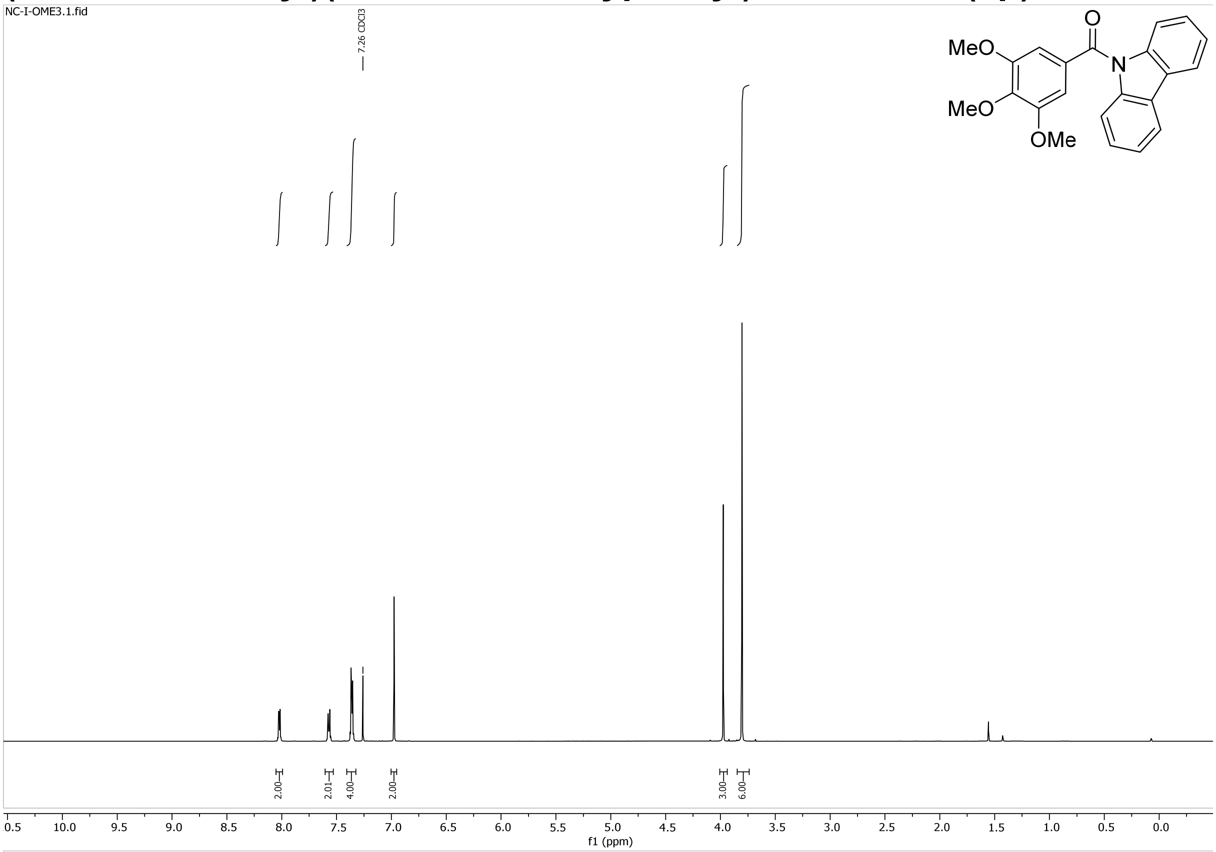

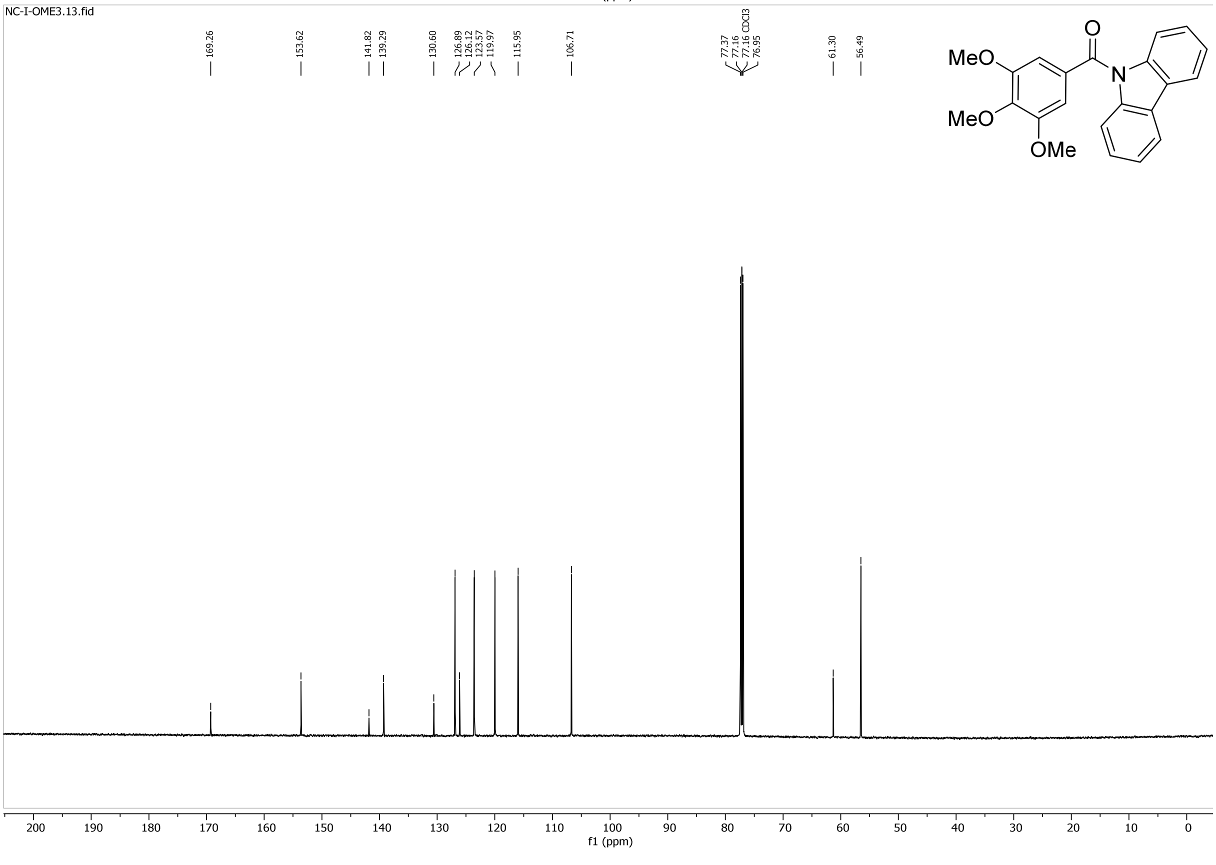

1-(9*H*-Carbazol-9-yl)-2-phenylethan-1-one (2r)

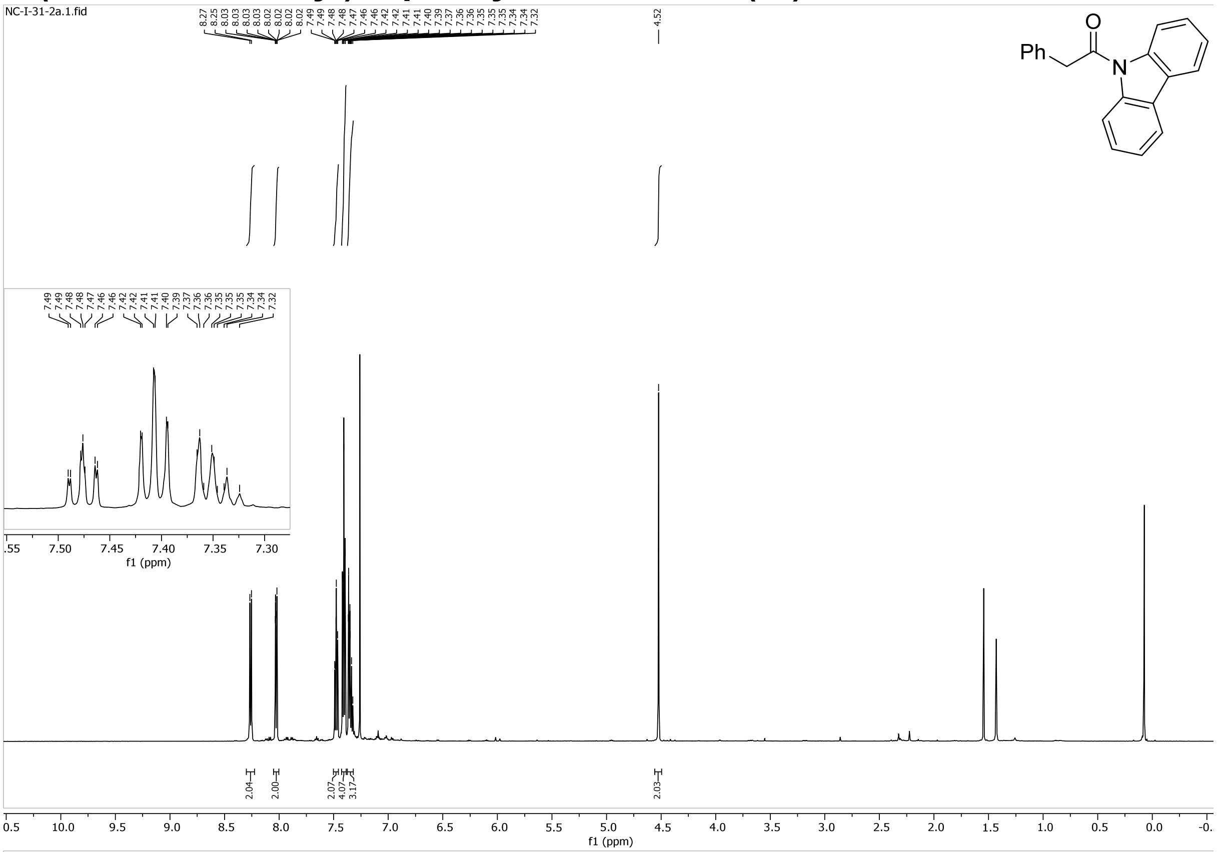

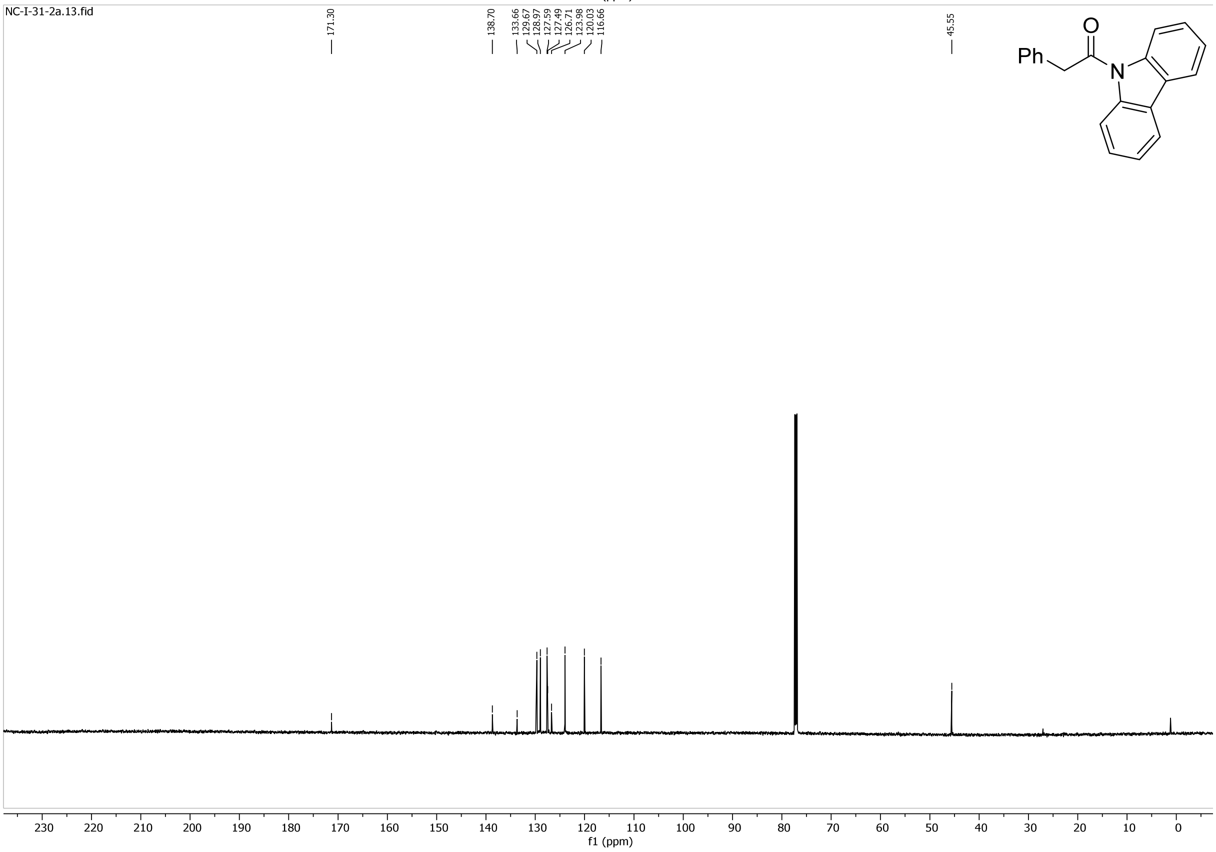

9-(2,4,6-Trimethylbenzoyl)-9*H*-carbazole-2-carbonitrile (2s)

The NMR spectrum of this substrate is broadened by rotational barriers

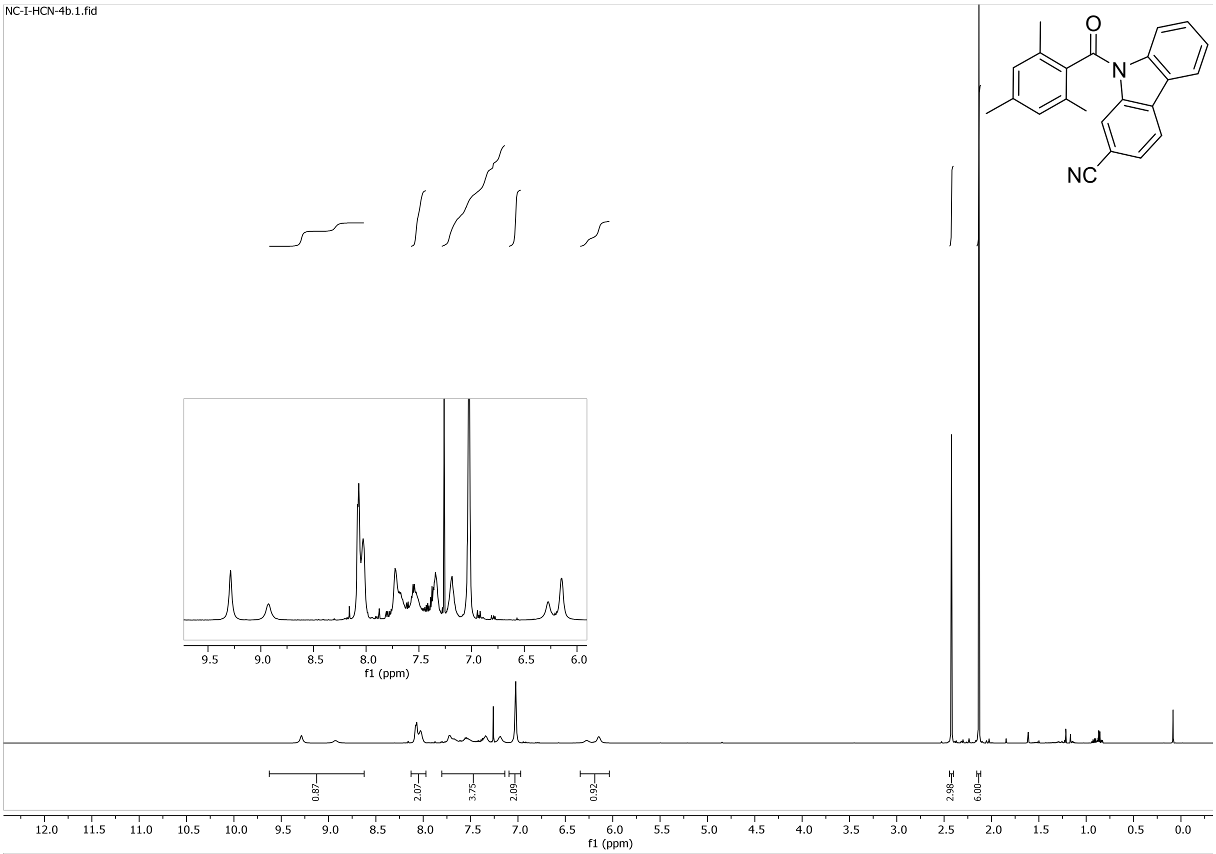

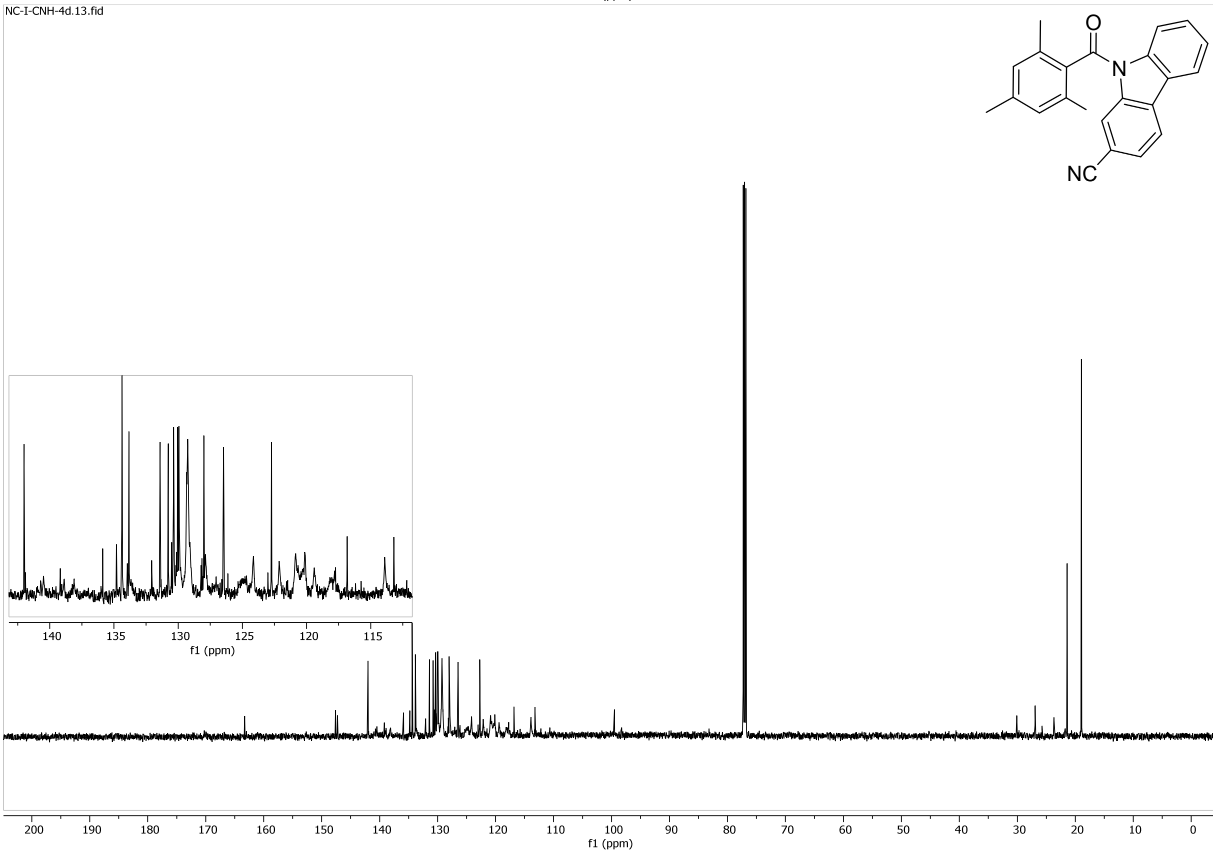

6-Bromo-9-(2,4,6-trimethylbenzoyl)-9*H*-carbazole-2-carbonitrile (2t)

The NMR spectrum of this substrate is broadened by rotational barriers

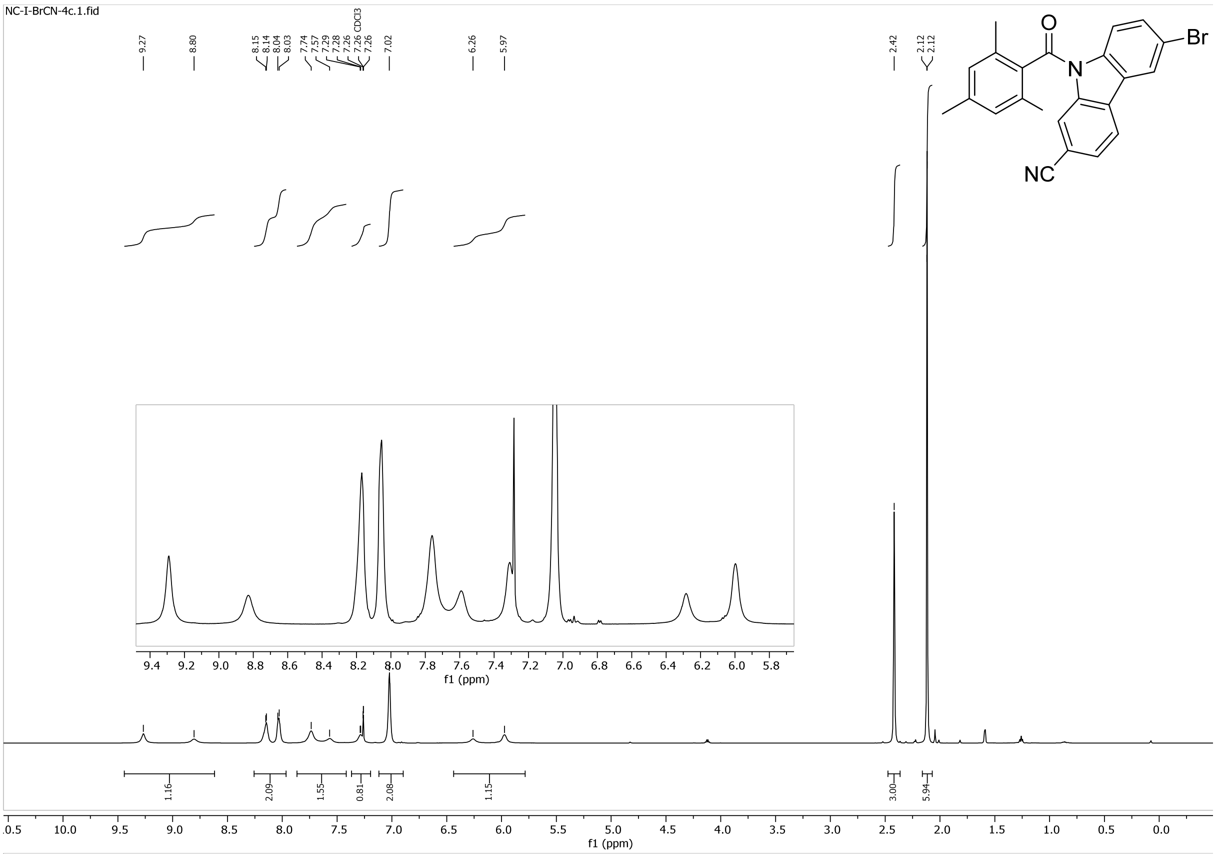

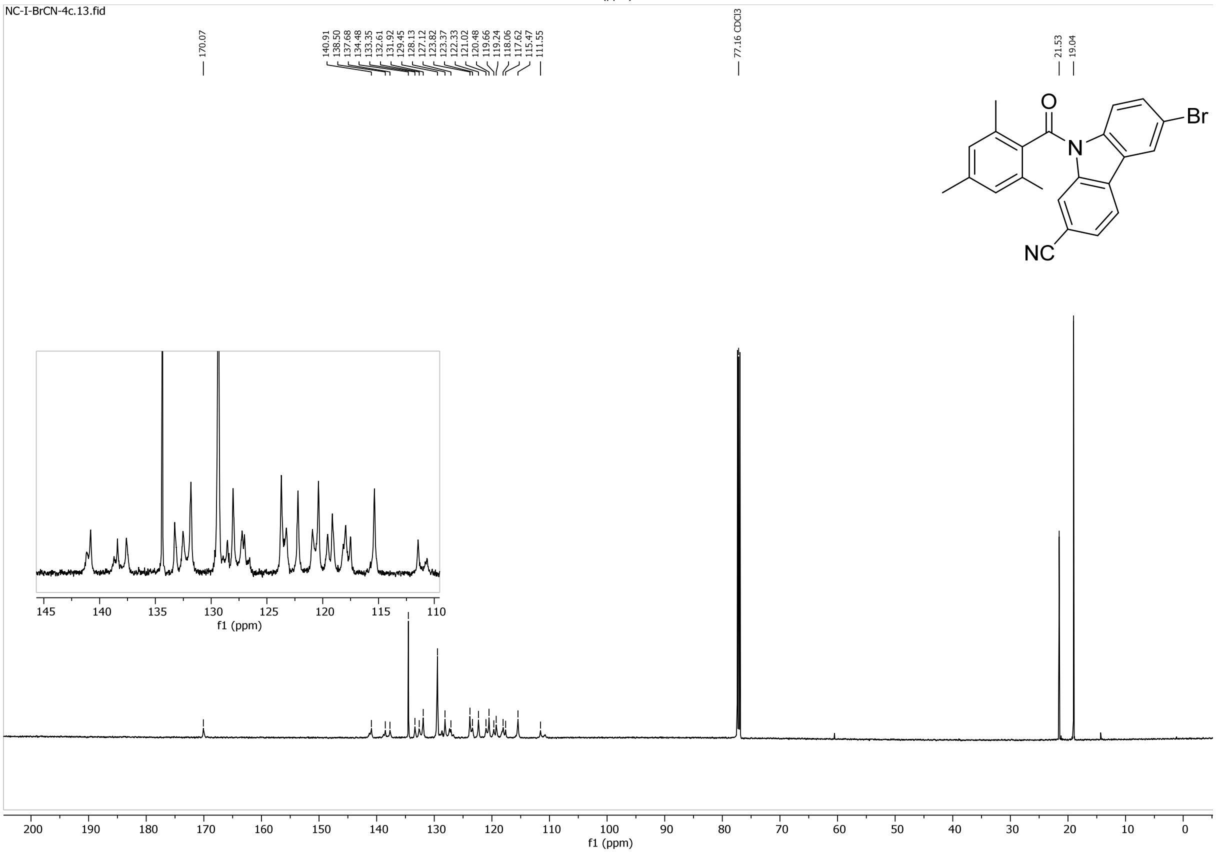

Acridin-10(9*H*)-yl(mesityl)methanone (3a)

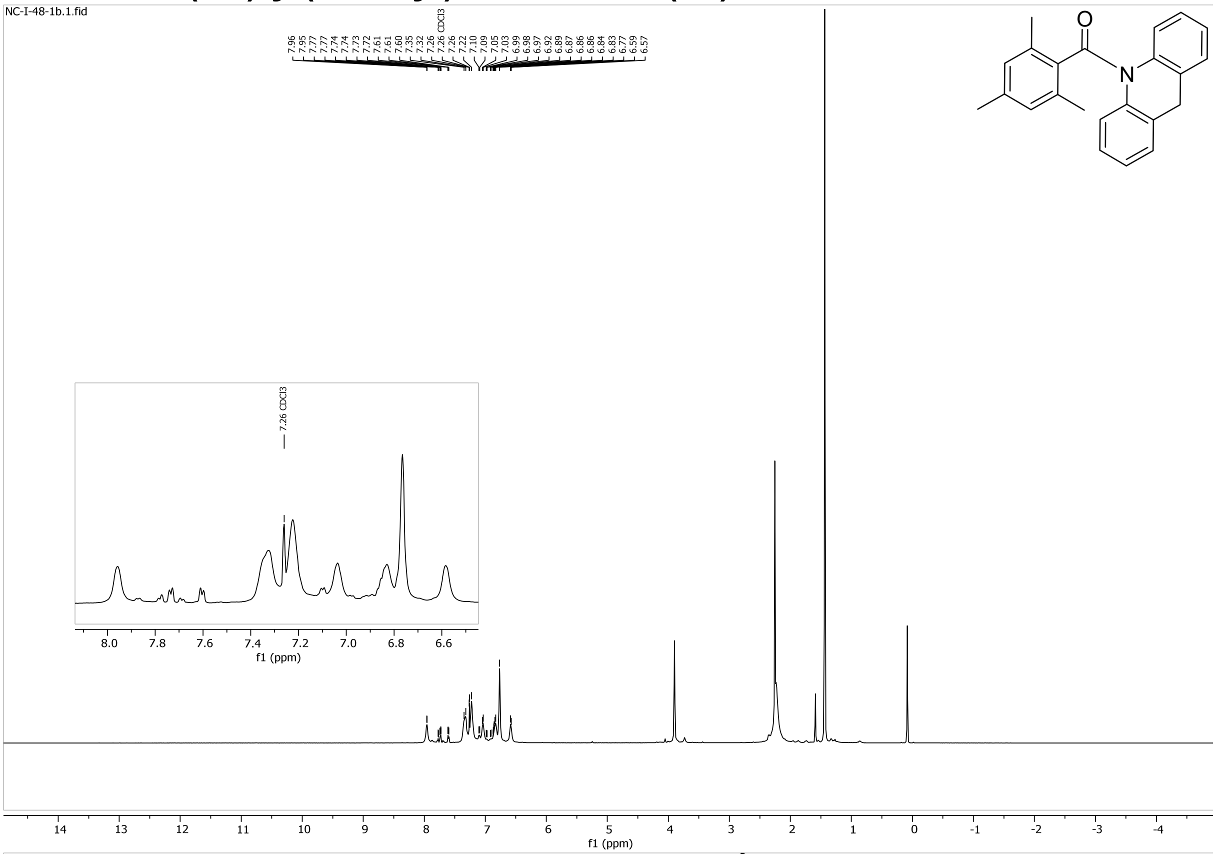

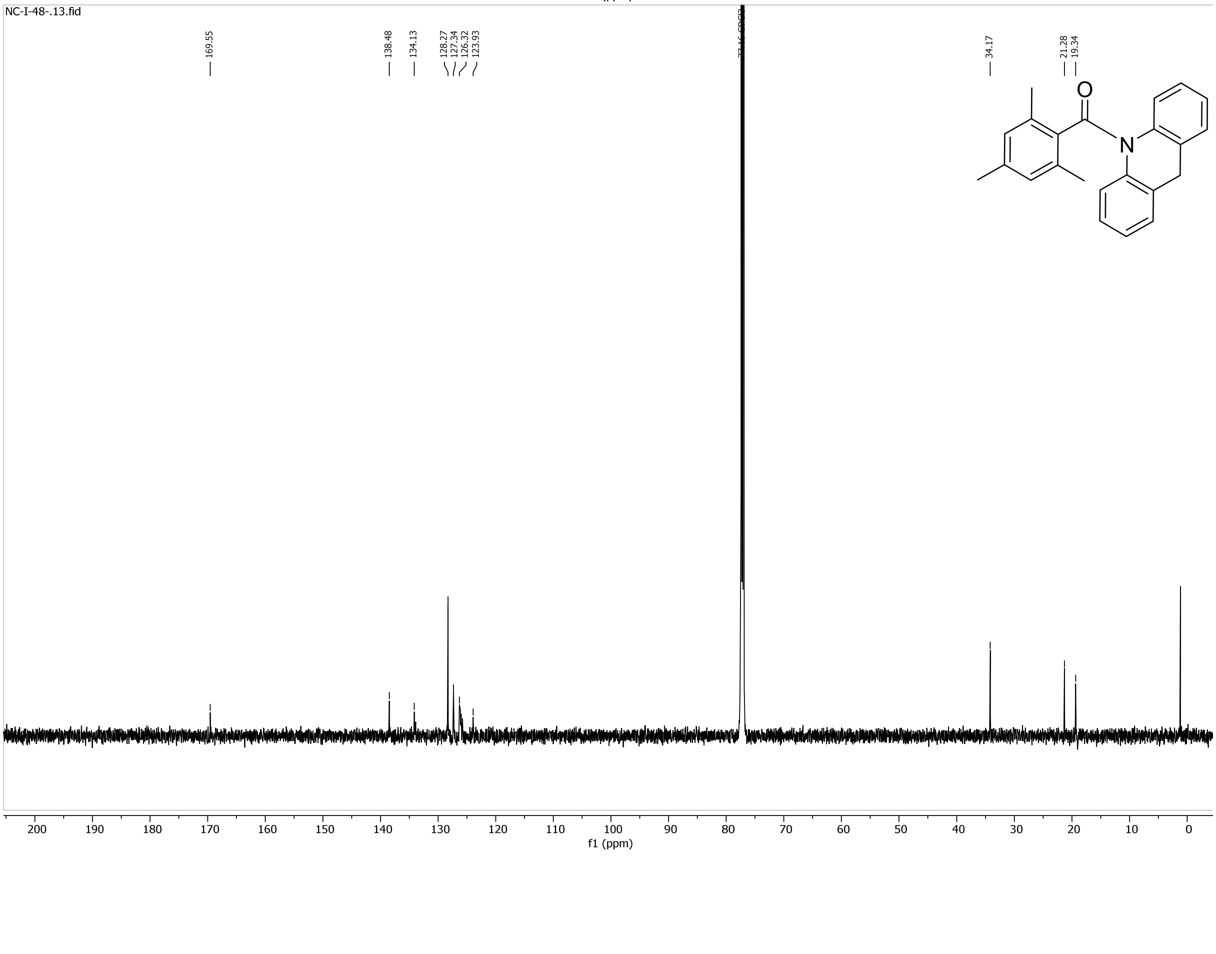

Mesityl(10*H*-phenoxazin-10-yl)methanone (3b) [14]

This substrate-class requires low-temperature measurements to give meaningful data

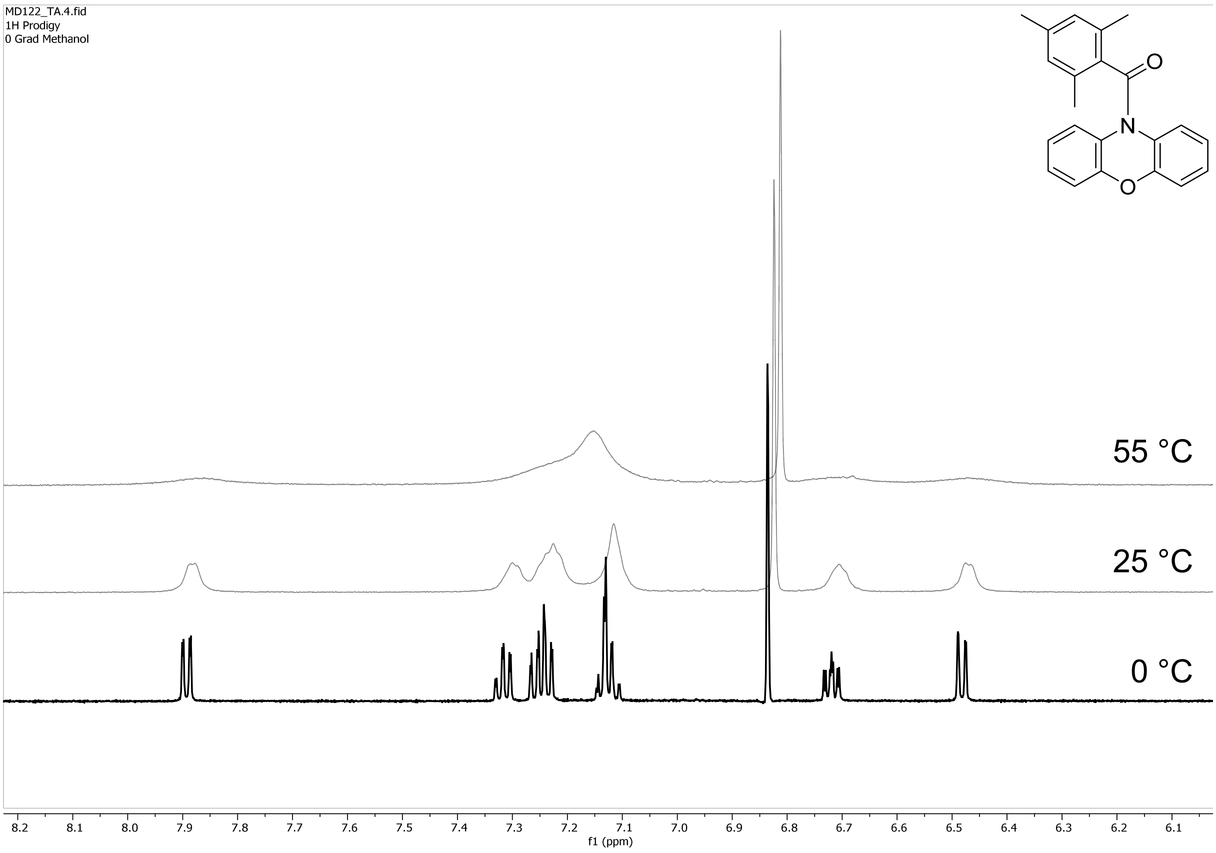

0 °C

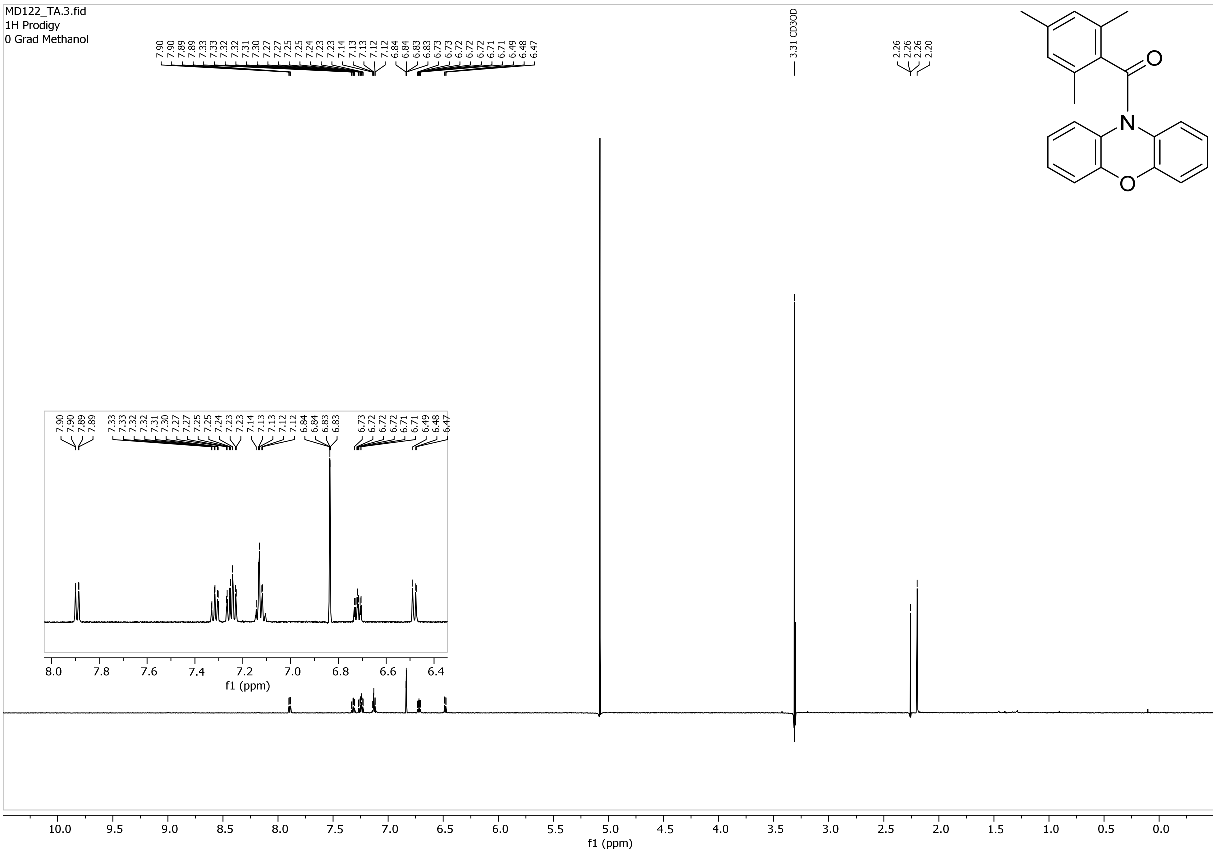

Mesityl(2-methyl-10*H*-phenoxazin-10-yl)methanone (3c)

Solubility of this compound made low-temperature NMR inefficient

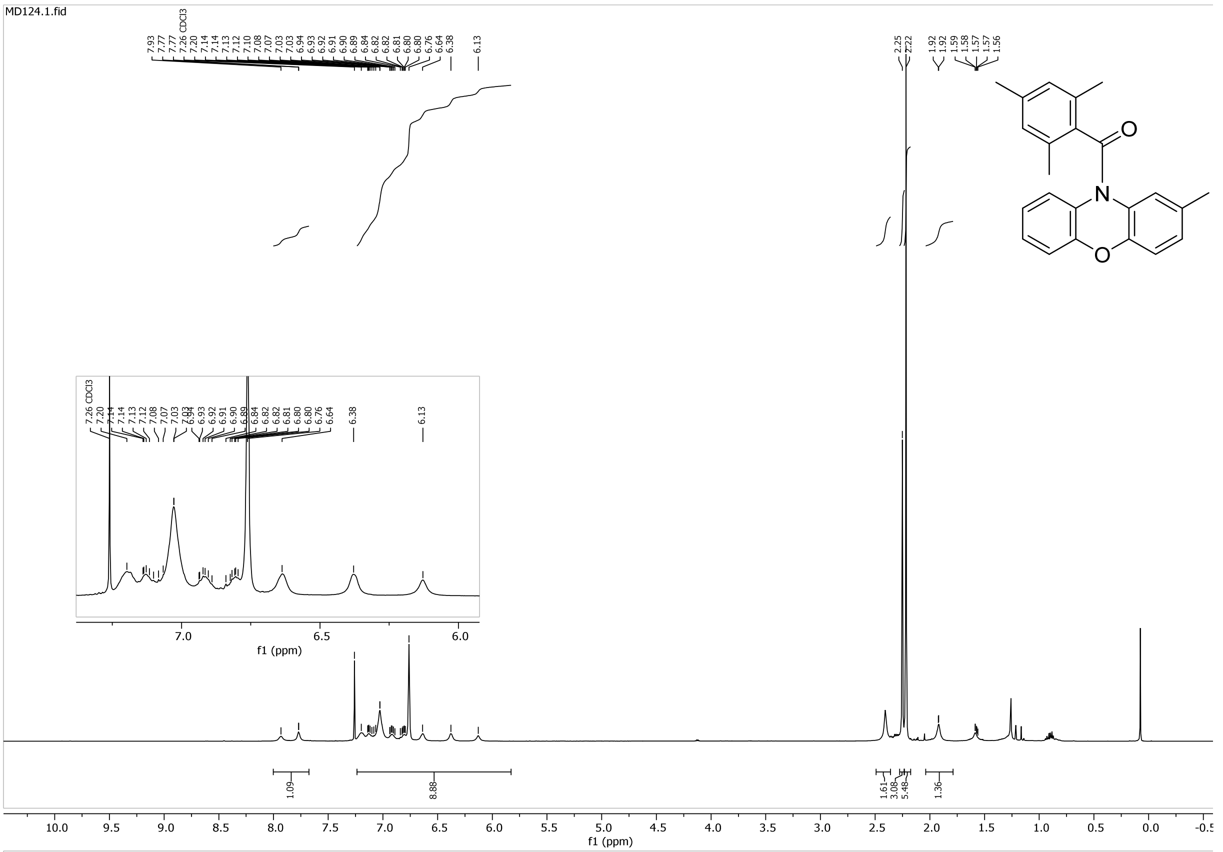

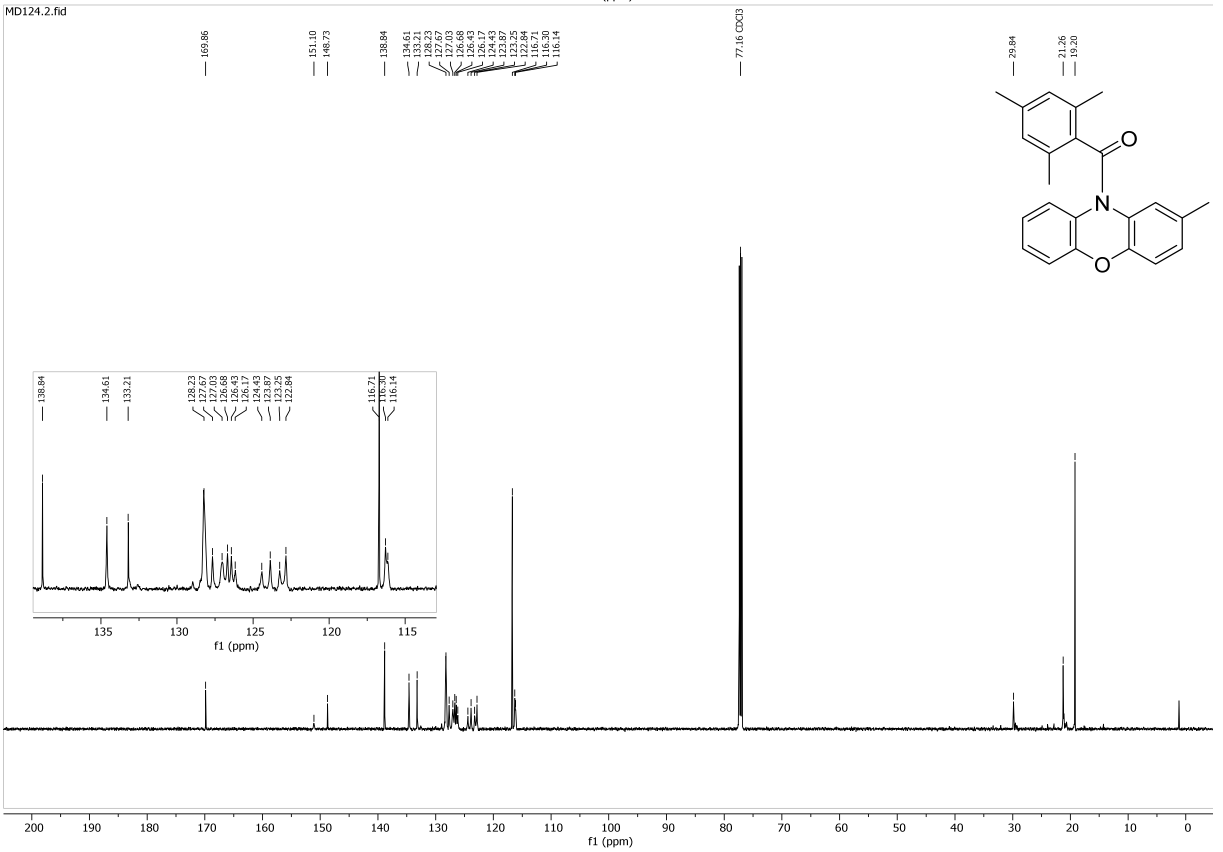

(2-Bromo-10*H*-phenoxazin-10-yl)(mesityl)methanone (3d)

Solubility of this compound made low-temperature NMR inefficient

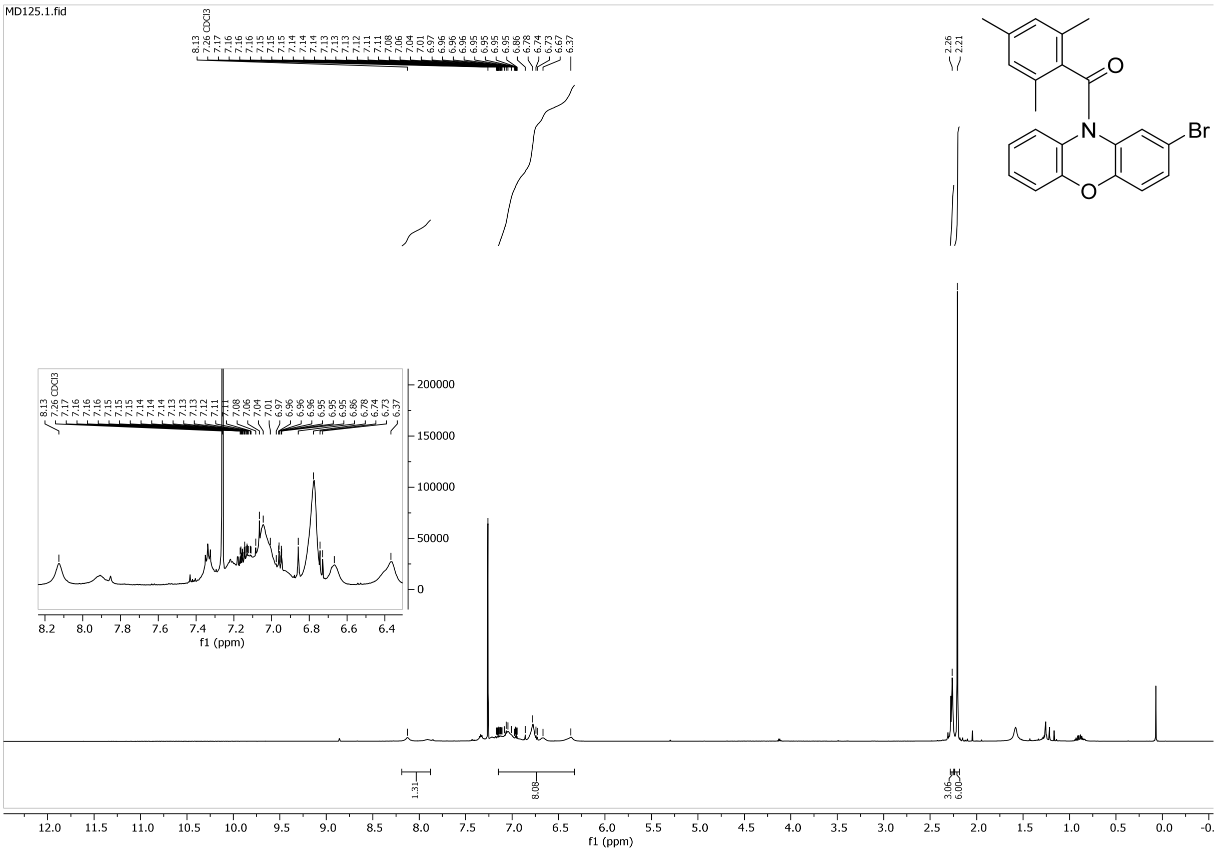

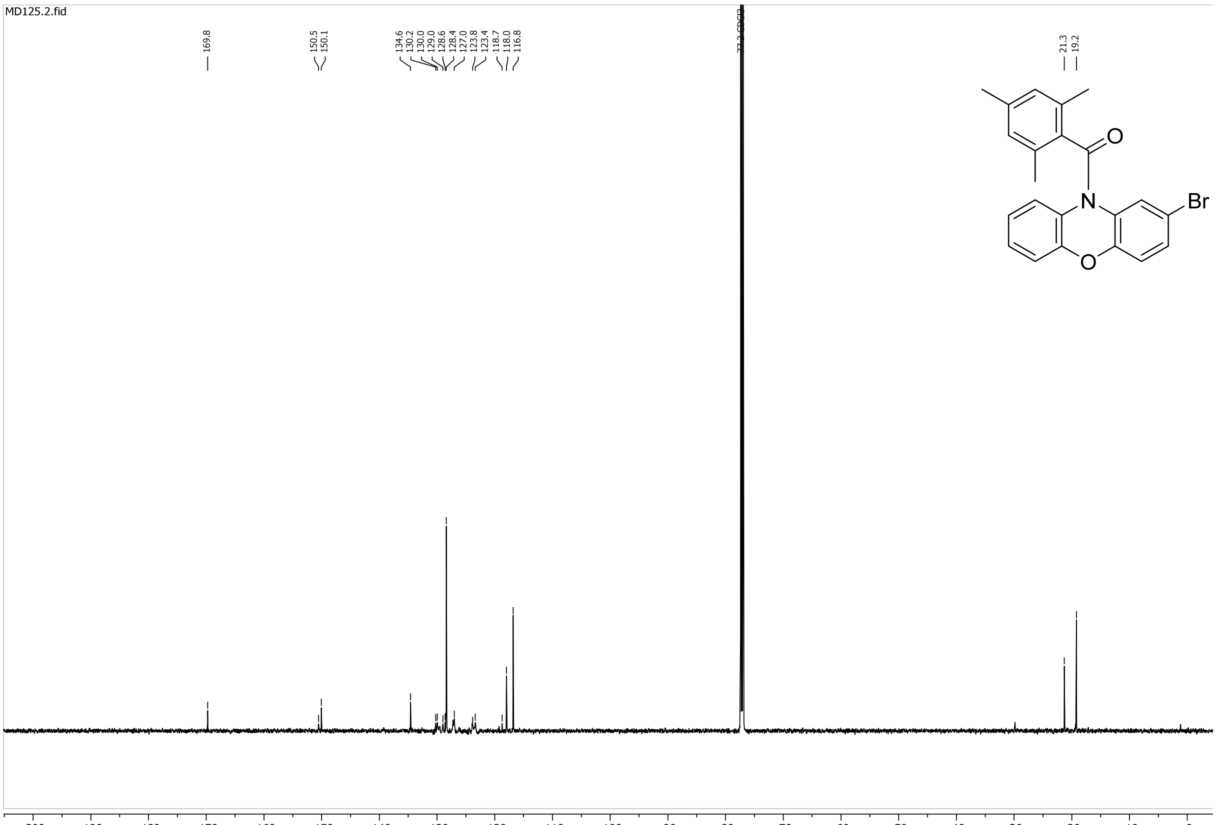

10-(2,4,6-Trimethylbenzoyl)-10*H*-phenoxazine-2-carbonitrile (3e)

Solubility of this compound made low-temperature NMR inefficient

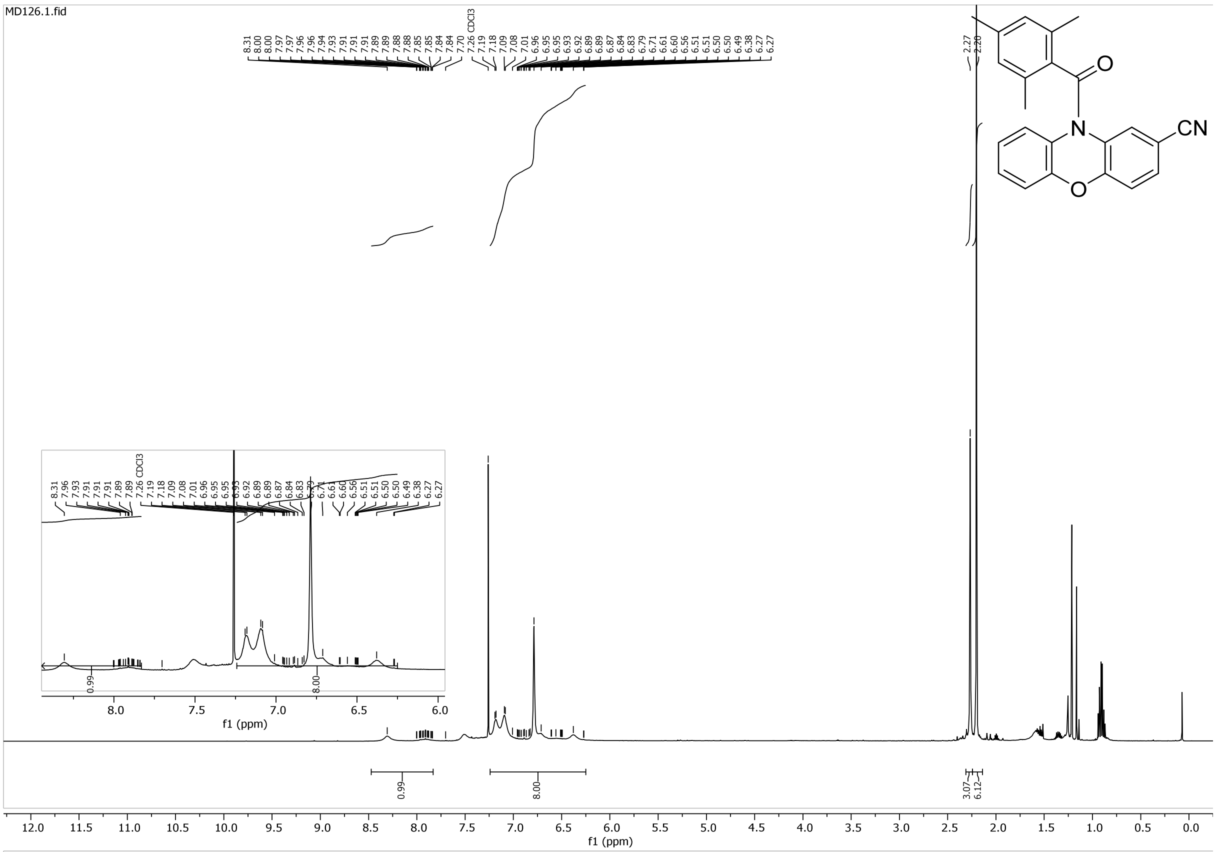

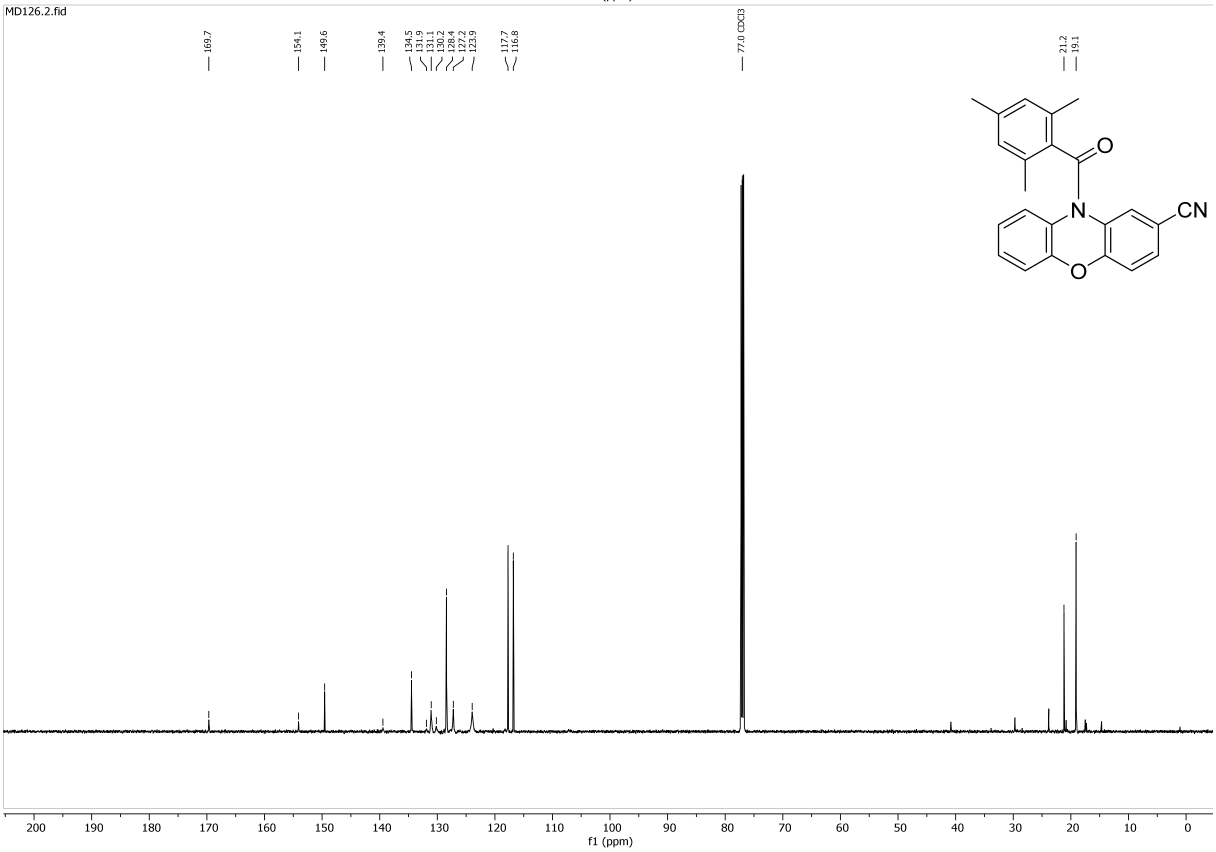

(6*H*-Imidazo[4,5,1-de]phenazin-6-yl)(phenyl)methanone (3f)

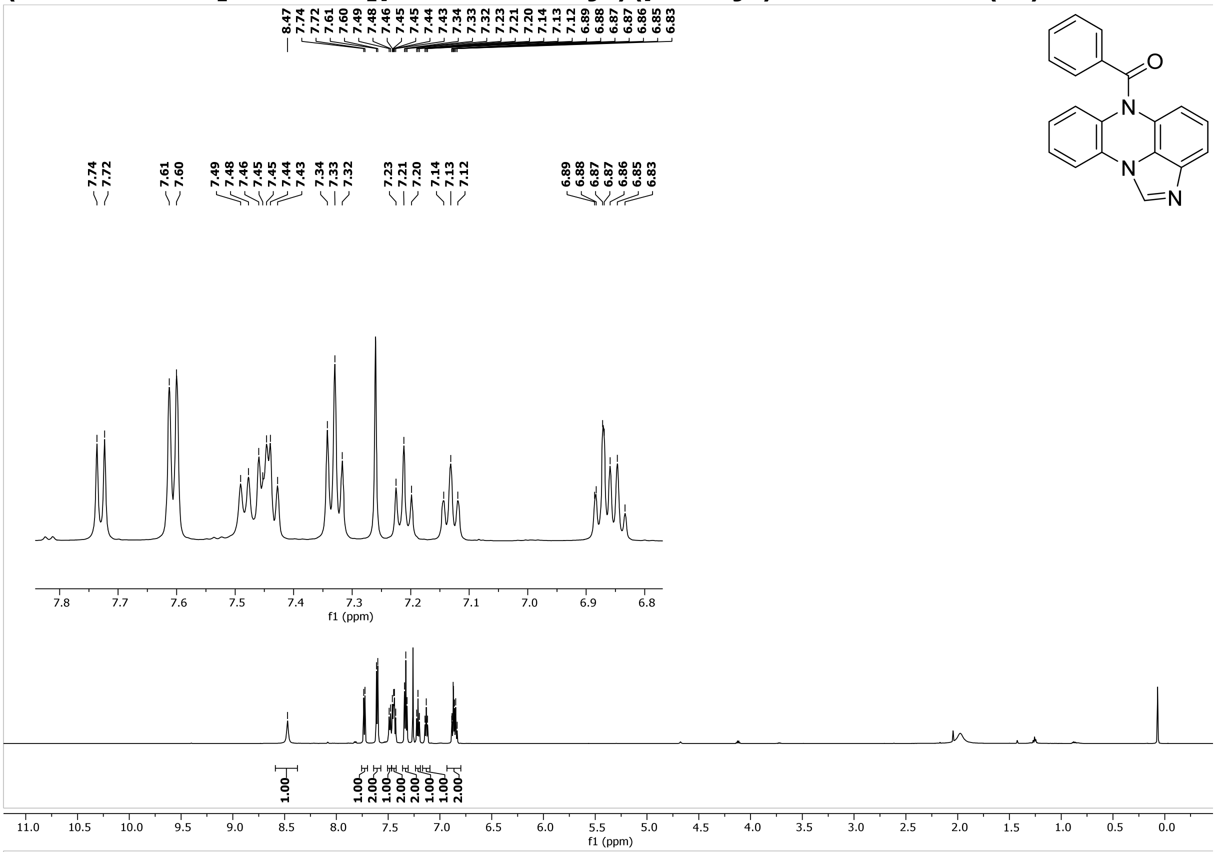

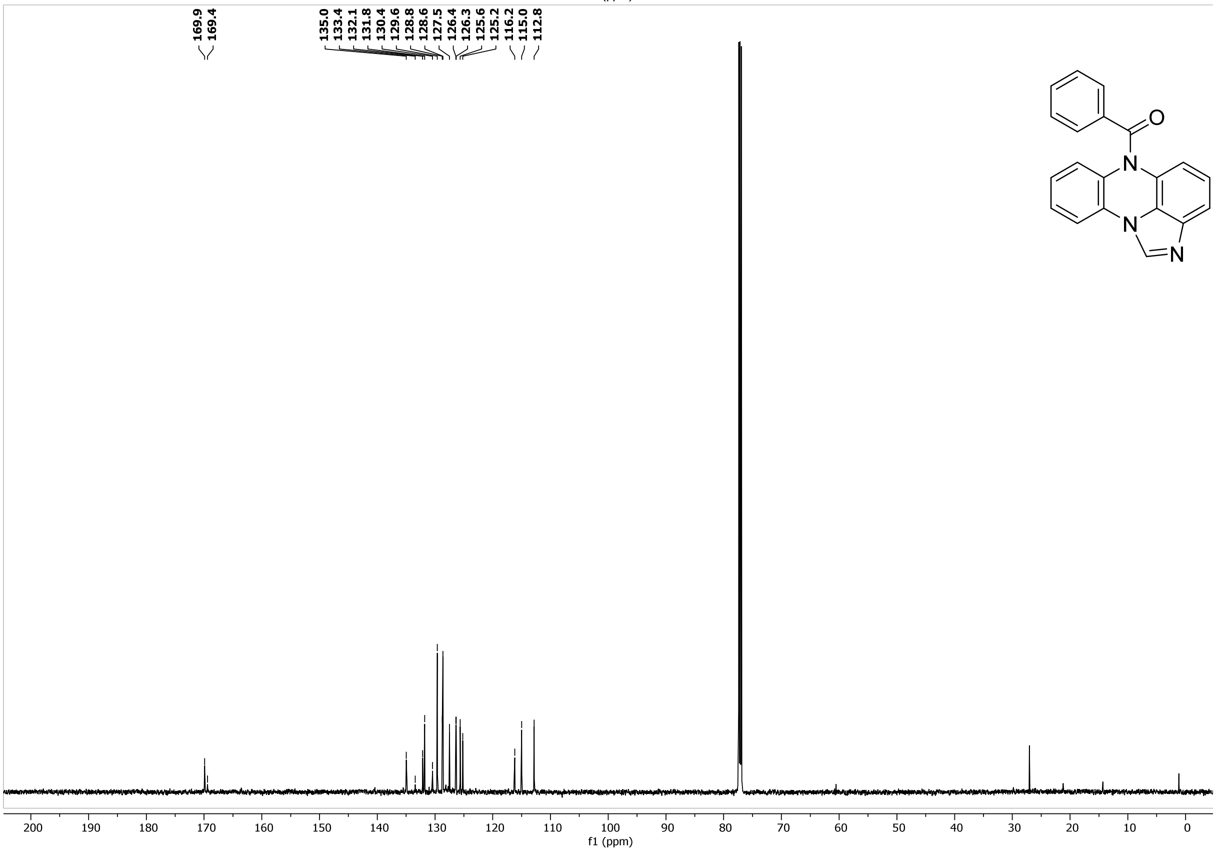

Crystallographic data

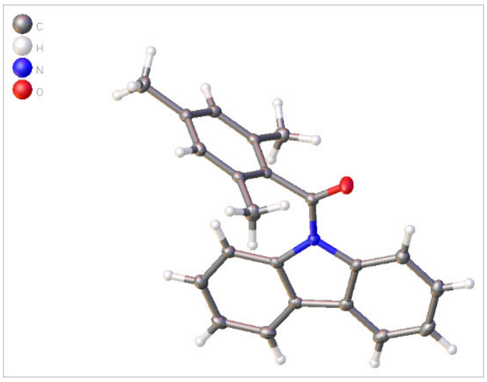

Table 1 Crystal data and structure refinement for ClamorN210312_0m.

CCDC 2288546

Identification code	ClamorN210312_0m
Empirical formula	$C_{22}H_{19}NO$
Formula weight	313.38
Temperature/K	100.00
Crystal system	monoclinic
Space group	$P2_1/n$
a/Å	10.4211(10)
b/Å	9.3263(7)
c/Å	17.4921(14)
α/°	90
β/°	104.003(3)
γ/°	90
Volume/Å^3	1649.5(2)
Z	4
ρ_{calc}g/cm^3	1.262
μ/mm^{-1}	0.077
F(000)	664.0
Crystal size/mm^3	0.28 × 0.27 × 0.24

Radiation	MoKα (λ = 0.71073)
2Θ range for data collection/°	4.8 to 54.202
Index ranges	-13 ≤ h ≤ 13, -11 ≤ k ≤ 11, -22 ≤ l ≤ 22
Reflections collected	43124
Independent reflections	3637 [R_{int} = 0.0694, R_{sigma} = 0.0353]
Data/restraints/parameters	3637/0/220
Goodness-of-fit on F^2	1.030
Final R indexes [I>=2σ (I)]	R_1 = 0.0429, wR_2 = 0.1056
Final R indexes [all data]	R_1 = 0.0520, wR_2 = 0.1114
Largest diff. peak/hole / e Å^{-3}	0.25/-0.31

Table 2 Fractional Atomic Coordinates (×10^4) and Equivalent Isotropic Displacement Parameters (Å^2×10^3) for ClamorN210312_0m. U_{eq} is defined as 1/3 of of the trace of the orthogonalised U_{IJ} tensor.

Atom	x	y	z	U(eq)
O1	4450.6(11)	1114.6(11)	2359.6(6)	27.3(2)
N1	5780.5(10)	1733.4(11)	3539.4(6)	15.2(2)
C1	6648.8(12)	2636.2(14)	4094.5(7)	14.7(3)
C4	5761.7(12)	358.0(13)	3893.7(7)	15.4(3)
C14	5109.9(12)	3535.7(13)	2465.4(7)	15.2(3)
C15	4253.9(12)	4569.6(14)	2640.5(7)	16.1(3)
C3	6607.7(12)	399.6(14)	4650.7(7)	16.1(3)
C12	5052.6(13)	-872.2(14)	3606.8(8)	18.7(3)
C2	7162.8(12)	1828.3(14)	4776.5(7)	16.0(3)
C5	7013.4(12)	4063.1(14)	4044.1(7)	17.2(3)
C6	7892.2(13)	4653.1(14)	4697.4(8)	19.3(3)
C13	5084.4(12)	2040.6(14)	2775.9(7)	16.4(3)

Table 2 Fractional Atomic Coordinates (×10[4]) and Equivalent Isotropic Displacement Parameters (Å[2]×10[3]) for ClamorN210312_0m. U_{eq} is defined as 1/3 of of the trace of the orthogonalised U_{IJ} tensor.

Atom	x	y	z	U(eq)
C18	5810.1(13)	5168.2(15)	1587.3(8)	18.9(3)
C16	4189.4(12)	5898.3(14)	2272.4(7)	18.0(3)
C17	4959.4(13)	6219.9(14)	1744.7(8)	18.6(3)
C9	6768.9(14)	-809.4(14)	5132.8(8)	20.5(3)
C8	8035.9(13)	2444.9(15)	5423.9(8)	20.0(3)
C7	8395.8(13)	3862.9(15)	5378.9(8)	21.0(3)
C10	6076.2(14)	-2040.0(14)	4847.7(8)	22.2(3)
C19	5895.3(12)	3817.3(14)	1935.8(7)	17.1(3)
C11	5227.7(14)	-2062.9(14)	4098.9(8)	21.4(3)
C20	3446.6(14)	4280.5(15)	3233.5(8)	21.2(3)
C22	6804.7(14)	2689.1(16)	1745.2(9)	25.3(3)
C21	4872.3(15)	7671.3(15)	1354.7(9)	25.9(3)

Table 3 Anisotropic Displacement Parameters (Å[2]×10[3]) for ClamorN210312_0m. The Anisotropic displacement factor exponent takes the form: $-2\pi^2[h^2a^{*2}U_{11}+2hka^*b^*U_{12}+\ldots]$.

Atom	U_{11}	U_{22}	U_{33}	U_{23}	U_{13}	U_{12}
O1	34.3(6)	21.0(5)	20.3(5)	1.0(4)	-5.7(4)	-9.2(4)
N1	15.4(5)	13.0(5)	15.7(5)	-0.4(4)	0.9(4)	-0.9(4)
C1	11.1(6)	17.6(6)	14.4(6)	-2.5(5)	1.4(4)	1.5(5)
C4	15.1(6)	15.0(6)	16.5(6)	1.6(5)	4.8(5)	3.0(5)
C14	12.5(6)	16.4(6)	14.0(6)	0.1(5)	-1.9(4)	-1.9(5)
C15	12.4(6)	19.8(6)	14.3(6)	0.4(5)	-0.3(4)	-1.5(5)
C3	13.9(6)	18.3(6)	16.7(6)	0.2(5)	4.9(5)	3.6(5)
C12	19.1(6)	17.6(6)	19.3(6)	-0.8(5)	4.6(5)	-0.8(5)

Table 3 Anisotropic Displacement Parameters (Å^2×10^3) for ClamorN210312_0m. The Anisotropic displacement factor exponent takes the form: -2π^2[h^{2}a*^{2}U$_{11}$+2hka*b*U$_{12}$+…].

Atom	U_{11}	U_{22}	U_{33}	U_{23}	U_{13}	U_{12}
C2	13.7(6)	18.3(6)	16.3(6)	0.4(5)	4.4(5)	3.4(5)
C5	15.6(6)	16.6(6)	17.9(6)	-0.1(5)	1.1(5)	1.1(5)
C6	16.8(6)	17.3(6)	22.8(6)	-3.5(5)	2.7(5)	-1.0(5)
C13	14.6(6)	17.9(6)	15.3(6)	0.4(5)	1.0(5)	-0.8(5)
C18	15.8(6)	22.3(7)	18.9(6)	1.8(5)	4.7(5)	-3.0(5)
C16	14.4(6)	18.7(6)	19.6(6)	0.5(5)	1.4(5)	1.6(5)
C17	15.7(6)	18.3(6)	19.8(6)	3.4(5)	0.6(5)	-2.2(5)
C9	22.2(7)	22.0(7)	17.8(6)	3.5(5)	5.5(5)	5.8(5)
C8	17.8(6)	24.1(7)	16.3(6)	-0.4(5)	0.7(5)	3.3(5)
C7	17.0(6)	25.1(7)	18.3(6)	-5.9(5)	-0.5(5)	-0.1(5)
C10	28.6(7)	17.4(6)	23.1(7)	5.5(5)	11.4(6)	4.7(5)
C19	13.3(6)	19.1(6)	17.3(6)	-1.7(5)	0.6(5)	-1.4(5)
C11	25.2(7)	15.8(6)	25.7(7)	-1.2(5)	11.1(6)	-1.4(5)
C20	20.2(7)	22.7(7)	22.1(6)	3.1(5)	8.0(5)	1.1(5)
C22	22.2(7)	23.6(7)	32.3(7)	-1.8(6)	11.0(6)	1.6(6)
C21	25.2(7)	21.8(7)	31.3(8)	8.7(6)	7.9(6)	-0.2(6)

Table 4 Bond Lengths for ClamorN210312_0m.

Atom	Atom	Length/Å	Atom	Atom	Length/Å
O1	C13	1.2163(16)	C3	C9	1.3937(18)
N1	C1	1.4302(15)	C12	C11	1.3897(18)
N1	C4	1.4268(16)	C2	C8	1.3936(18)
N1	C13	1.3867(16)	C5	C6	1.3931(18)
C1	C2	1.4030(17)	C6	C7	1.3917(19)

Table 4 Bond Lengths for ClamorN210312_0m.

Atom	Atom	Length/Å	Atom	Atom	Length/Å
C1	C5	1.3927(18)	C18	C17	1.3935(19)
C4	C3	1.4026(17)	C18	C19	1.3931(18)
C4	C12	1.3910(18)	C16	C17	1.3945(18)
C14	C15	1.3969(18)	C17	C21	1.5087(18)
C14	C13	1.4990(17)	C9	C10	1.383(2)
C14	C19	1.4014(18)	C8	C7	1.382(2)
C15	C16	1.3906(18)	C10	C11	1.392(2)
C15	C20	1.5096(18)	C19	C22	1.5064(18)
C3	C2	1.4480(18)			

Table 5 Bond Angles for ClamorN210312_0m.

Atom	Atom	Atom	Angle/˚	Atom	Atom	Atom	Angle/˚
C4	N1	C1	107.89(10)	C8	C2	C1	120.61(12)
C13	N1	C1	129.04(11)	C8	C2	C3	131.41(12)
C13	N1	C4	123.03(10)	C1	C5	C6	117.43(12)
C2	C1	N1	108.05(11)	C7	C6	C5	122.02(13)
C5	C1	N1	131.07(11)	O1	C13	N1	120.77(12)
C5	C1	C2	120.87(11)	O1	C13	C14	119.88(11)
C3	C4	N1	108.28(11)	N1	C13	C14	119.35(11)
C12	C4	N1	130.30(11)	C19	C18	C17	121.76(12)
C12	C4	C3	121.41(12)	C15	C16	C17	121.80(12)
C15	C14	C13	119.63(11)	C18	C17	C16	118.36(12)
C15	C14	C19	121.44(12)	C18	C17	C21	120.91(12)
C19	C14	C13	118.57(11)	C16	C17	C21	120.74(12)
C14	C15	C20	121.27(11)	C10	C9	C3	118.50(12)

Table 5 Bond Angles for ClamorN210312_0m.

Atom	Atom	Atom	Angle/˚	Atom	Atom	Atom	Angle/˚
C16	C15	C14	118.40(12)	C7	C8	C2	118.77(12)
C16	C15	C20	120.31(12)	C8	C7	C6	120.28(12)
C4	C3	C2	107.80(11)	C9	C10	C11	120.62(12)
C9	C3	C4	120.34(12)	C14	C19	C22	120.98(12)
C9	C3	C2	131.86(12)	C18	C19	C14	118.24(12)
C11	C12	C4	117.19(12)	C18	C19	C22	120.78(12)
C1	C2	C3	107.97(11)	C12	C11	C10	121.93(13)

Table 6 Torsion Angles for ClamorN210312_0m.

A	B	C	D	Angle/˚	A	B	C	D	Angle/˚
N1	C1	C2	C3	-0.21(13)	C3	C9	C10	C11	-0.6(2)
N1	C1	C2	C8	-179.40(11)	C12	C4	C3	C2	-178.47(12)
N1	C1	C5	C6	179.82(12)	C12	C4	C3	C9	0.77(19)
N1	C4	C3	C2	0.31(13)	C2	C1	C5	C6	-0.65(18)
N1	C4	C3	C9	179.55(11)	C2	C3	C9	C10	178.91(13)
N1	C4	C12	C11	-179.11(12)	C2	C8	C7	C6	-0.04(19)
C1	N1	C4	C3	-0.44(13)	C5	C1	C2	C3	-179.84(11)
C1	N1	C4	C12	178.20(13)	C5	C1	C2	C8	0.98(19)
C1	N1	C13	O1	175.08(12)	C5	C6	C7	C8	0.4(2)
C1	N1	C13	C14	-5.34(19)	C13	N1	C1	C2	-177.47(12)
C1	C2	C8	C7	-0.61(19)	C13	N1	C1	C5	2.1(2)
C1	C5	C6	C7	0.0(2)	C13	N1	C4	C3	177.59(11)
C4	N1	C1	C2	0.40(13)	C13	N1	C4	C12	-3.8(2)
C4	N1	C1	C5	179.98(13)	C13	C14	C15	C16	-172.72(11)
C4	N1	C13	O1	-2.51(19)	C13	C14	C15	C20	9.24(18)

Table 6 Torsion Angles for ClamorN210312_0m.

A	B	C	D	Angle/˚	A	B	C	D	Angle/˚
C4	N1	C13	C14	177.07(11)	C13	C14	C19	C18	173.50(11)
C4	C3	C2	C1	-0.06(14)	C13	C14	C19	C22	-6.29(18)
C4	C3	C2	C8	179.00(13)	C17	C18	C19	C14	-0.89(19)
C4	C3	C9	C10	-0.12(19)	C17	C18	C19	C22	178.90(12)
C4	C12	C11	C10	-0.1(2)	C9	C3	C2	C1	-179.18(13)
C14	C15	C16	C17	-0.56(19)	C9	C3	C2	C8	-0.1(2)
C15	C14	C13	O1	97.01(15)	C9	C10	C11	C12	0.8(2)
C15	C14	C13	N1	-82.58(15)	C19	C14	C15	C16	0.30(18)
C15	C14	C19	C18	0.41(18)	C19	C14	C15	C20	-177.75(12)
C15	C14	C19	C22	-179.38(12)	C19	C14	C13	O1	-76.21(16)
C15	C16	C17	C18	0.11(19)	C19	C14	C13	N1	104.21(14)
C15	C16	C17	C21	-179.87(12)	C19	C18	C17	C16	0.64(19)
C3	C4	C12	C11	-0.62(19)	C19	C18	C17	C21	-179.38(12)
C3	C2	C8	C7	-179.58(13)	C20	C15	C16	C17	177.50(12)

Table 7 Hydrogen Atom Coordinates (Å×10^4) and Isotropic Displacement Parameters (Å^2×10^3) for ClamorN210312_0m.

Atom	x	y	z	U(eq)
H12	4472.96	-897.43	3096.01	22
H5	6675.87	4613.05	3582.02	21
H6	8155.8	5624.68	4677.08	23
H18	6346.35	5378.16	1232.91	23
H16	3604.68	6606.41	2383.61	22
H9	7341.73	-788.77	5645.74	25
H8	8377.33	1900.55	5887.58	24
H7	8989.49	4299.99	5814.96	25

Table 7 Hydrogen Atom Coordinates (Å×10^4) and Isotropic Displacement Parameters (Å^2×10^3) for ClamorN210312_0m.

Atom	x	y	z	U(eq)
H10	6180.49	-2877.49	5165.94	27
H11	4754.37	-2917.54	3918.93	26
H20A	3157.13	3277.72	3192.76	32
H20B	2671.16	4910.74	3127.06	32
H20C	3987.6	4465.49	3765.81	32
H22A	7454.54	2413.36	2227.67	38
H22B	7264.73	3073.42	1362.97	38
H22C	6286.5	1846.58	1520.94	38
H21A	5319	8385.62	1738.7	39
H21B	3941.35	7938.82	1157.9	39
H21C	5300.14	7632.29	914.05	39

Crystal structure determination of [ClamorN210312_0m]

Crystal Data for $C_{22}H_{19}NO$ (*M* =313.38 g/mol): monoclinic, space group P2$_1$/n (no. 14), *a* = 10.4211(10) Å, *b* = 9.3263(7) Å, *c* = 17.4921(14) Å, *β* = 104.003(3)°, *V* = 1649.5(2) Å^3, *Z* = 4, *T* = 100.00 K, μ(MoKα) = 0.077 mm^{-1}, *Dcalc* = 1.262 g/cm^3, 43124 reflections measured (4.8° ≤ 2Θ ≤ 54.202°), 3637 unique (R_{int} = 0.0694, R_{sigma} = 0.0353) which were used in all calculations. The final R_1 was 0.0429 (I > 2σ(I)) and wR_2 was 0.1114 (all data).

Refinement model description

Number of restraints - 0, number of constraints - unknown.

Details:

1. Fixed Uiso
 At 1.2 times of:
  All C(H) groups
 At 1.5 times of:
  All C(H,H,H) groups
2.a Aromatic/amide H refined with riding coordinates:
 C12(H12), C5(H5), C6(H6), C18(H18), C16(H16), C9(H9), C8(H8), C7(H7), C10(H10), C11(H11)
2.b Idealised Me refined as rotating group:
 C20(H20A,H20B,H20C), C22(H22A,H22B,H22C), C21(H21A,H21B,H21C)

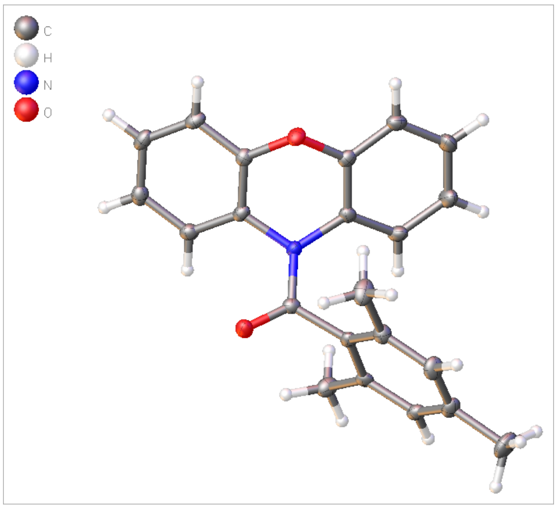

Table 1 Crystal data and structure refinement for DamrathM201007_0m.

CCDC 2288547

Empirical formula	$C_{22}H_{19}NO_2$
Formula weight	329.38
Temperature/K	100.0
Crystal system	monoclinic
Space group	$P2_1/c$
a/Å	8.9727(5)
b/Å	12.4511(6)
c/Å	16.2174(9)
α/°	90
β/°	103.216(2)
γ/°	90
Volume/Å^3	1763.82(16)
Z	4
ρ_{calc}g/cm^3	1.240
μ/mm^{-1}	0.079
F(000)	696.0

Crystal size/mm^3	0.19 × 0.18 × 0.14
Radiation	MoKα (λ = 0.71073)
2Θ range for data collection/°	4.166 to 52.732
Index ranges	-11 ≤ h ≤ 11, -15 ≤ k ≤ 15, -20 ≤ l ≤ 20
Reflections collected	40307
Independent reflections	3607 [R_{int} = 0.0678, R_{sigma} = 0.0332]
Data/restraints/parameters	3607/0/229
Goodness-of-fit on F^2	1.051
Final R indexes [I>=2σ (I)]	R_1 = 0.0423, wR_2 = 0.1021
Final R indexes [all data]	R_1 = 0.0548, wR_2 = 0.1090
Largest diff. peak/hole / e Å^{-3}	0.23/-0.29

Table 2 Fractional Atomic Coordinates (×10^4) and Equivalent Isotropic Displacement Parameters (Å^2×10^3) for DamrathM201007_0m. U_{eq} is defined as 1/3 of of the trace of the orthogonalised U_{IJ} tensor.

Atom	x	y	z	U(eq)
O1	4255.3(11)	5932.2(8)	8281.7(6)	19.1(2)
O2	9101.6(11)	5231.5(9)	8130.8(6)	21.1(2)
N1	6684.4(13)	4675.9(9)	8116.0(7)	15.6(3)
C12	6709.0(16)	5168.9(11)	8921.3(9)	16.1(3)
C13	7926.2(16)	4794.2(11)	7752.2(9)	15.5(3)
C19	8421.3(16)	3324.8(12)	6795.1(9)	16.9(3)
C11	7884.4(17)	5013.2(12)	9634.9(9)	19.1(3)
C14	7797.1(16)	4339.1(11)	6878.6(9)	15.9(3)
C18	8352.5(16)	2933.2(12)	5984.3(9)	19.0(3)
C1	3974.3(16)	5042.0(11)	7752.3(9)	16.8(3)
C7	5468.1(17)	5802.5(11)	8978.2(9)	16.6(3)
C9	6600.3(18)	6185.3(12)	10426.0(9)	21.0(3)

C8	5406.7(17)	6325.6(11)	9724.2(9)	19.3(3)
C6	5172.9(16)	4375.7(12)	7659.2(9)	16.4(3)
C16	7137.7(18)	4544.3(13)	5369.2(9)	24.0(3)
C15	7161.3(17)	4961.6(12)	6168.9(9)	19.8(3)
C5	4845.1(17)	3459.2(12)	7164.6(9)	20.7(3)
C10	7824.2(18)	5523.3(12)	10388.1(9)	21.3(3)
C17	7724.8(18)	3533.3(13)	5266.4(9)	22.7(3)
C22	9154.3(18)	2671.2(12)	7560.4(9)	22.8(3)
C2	2484.3(17)	4838.2(12)	7332.1(9)	20.7(3)
C4	3350.4(18)	3264.9(13)	6723.1(10)	26.1(4)
C3	2182.2(18)	3958.4(14)	6797.3(10)	25.8(4)
C20	6538(2)	6065.4(13)	6268.9(11)	29.7(4)
C21	7718(2)	3095.5(15)	4397.6(10)	33.9(4)

Table 3 Anisotropic Displacement Parameters (Å^2×10^3) for DamrathM201007_0m. The Anisotropic displacement factor exponent takes the form: $-2\pi^2[h^2a^{*2}U_{11}+2hka^*b^*U_{12}+\dots]$.

Atom	U_{11}	U_{22}	U_{33}	U_{23}	U_{13}	U_{12}
O1	21.5(5)	16.5(5)	19.3(5)	-1.6(4)	4.3(4)	3.6(4)
O2	18.1(5)	26.8(6)	19.0(5)	-2.9(4)	5.3(4)	-5.1(4)
N1	15.4(6)	17.1(6)	15.0(6)	-2.3(5)	4.9(5)	-1.6(5)
C12	19.0(7)	14.9(7)	16.1(7)	-1.8(5)	7.7(6)	-2.7(5)
C13	15.9(7)	14.7(7)	16.6(7)	2.1(5)	5.1(6)	1.4(5)
C19	13.1(7)	20.6(7)	18.6(7)	0.8(6)	7.3(5)	-2.3(6)
C11	17.9(7)	20.1(7)	20.7(7)	0.2(6)	7.2(6)	-0.7(6)
C14	14.4(7)	18.7(7)	15.8(7)	-0.4(6)	5.9(5)	-4.0(5)
C18	18.7(7)	19.7(7)	20.9(7)	-2.3(6)	9.7(6)	-2.5(6)
C1	21.0(7)	15.5(7)	15.4(7)	1.2(5)	7.0(6)	-0.9(6)

Table 3 Anisotropic Displacement Parameters (Å^2×10^3) for DamrathM201007_0m. The Anisotropic displacement factor exponent takes the form: -$2\pi^2[h^2a^{*2}U_{11}+2hka^*b^*U_{12}+\ldots]$.

Atom	U_{11}	U_{22}	U_{33}	U_{23}	U_{13}	U_{12}
C7	19.4(7)	14.3(7)	16.4(7)	1.5(6)	4.7(6)	-1.1(6)
C9	29.1(8)	18.1(7)	18.1(7)	-3.7(6)	10.3(6)	-5.7(6)
C8	24.1(8)	14.5(7)	22.1(7)	-1.2(6)	10.9(6)	0.2(6)
C6	15.3(7)	19.1(7)	16.1(7)	1.2(5)	6.0(6)	-2.1(6)
C16	30.0(9)	24.7(8)	16.4(7)	5.1(6)	3.5(6)	-2.7(7)
C15	20.8(8)	18.6(7)	19.7(7)	1.6(6)	3.9(6)	-2.4(6)
C5	18.3(8)	20.6(8)	25.9(8)	-4.0(6)	10.8(6)	-2.2(6)
C10	23.2(8)	23.4(8)	16.9(7)	0.5(6)	4.0(6)	-5.3(6)
C17	26.3(8)	26.8(8)	16.7(7)	-1.1(6)	9.0(6)	-5.8(6)
C22	27.7(8)	21.5(8)	20.2(8)	2.5(6)	7.5(6)	4.1(6)
C2	18.6(8)	23.9(8)	21.2(7)	1.5(6)	8.2(6)	2.1(6)
C4	22.4(8)	27.5(8)	30.1(9)	-11.4(7)	9.8(7)	-7.3(7)
C3	16.3(7)	35.2(9)	26.4(8)	-4.9(7)	5.9(6)	-6.8(7)
C20	39.3(10)	21.5(8)	24.8(8)	2.1(7)	0.1(7)	4.8(7)
C21	48.9(11)	37.1(10)	17.8(8)	-2.3(7)	12.4(8)	0.5(8)

Table 4 Bond Lengths for DamrathM201007_0m.

Atom	Atom	Length/Å	Atom	Atom	Length/Å
O1	C1	1.3894(17)	C18	C17	1.390(2)
O1	C7	1.3871(18)	C1	C6	1.394(2)
O2	C13	1.2205(17)	C1	C2	1.378(2)
N1	C12	1.4388(17)	C7	C8	1.386(2)
N1	C13	1.3826(18)	C9	C8	1.384(2)
N1	C6	1.4382(18)	C9	C10	1.386(2)

Table 4 Bond Lengths for DamrathM201007_0m.

Atom	Atom	Length/Å	Atom	Atom	Length/Å
C12	C11	1.390(2)	C6	C5	1.387(2)
C12	C7	1.385(2)	C16	C15	1.393(2)
C13	C14	1.5056(19)	C16	C17	1.389(2)
C19	C14	1.401(2)	C15	C20	1.506(2)
C19	C18	1.390(2)	C5	C4	1.390(2)
C19	C22	1.504(2)	C17	C21	1.510(2)
C11	C10	1.389(2)	C2	C3	1.385(2)
C14	C15	1.397(2)	C4	C3	1.384(2)

Table 5 Bond Angles for DamrathM201007_0m.

Atom	Atom	Atom	Angle/˚	Atom	Atom	Atom	Angle/˚
C7	O1	C1	114.08(11)	C12	C7	O1	120.35(12)
C13	N1	C12	119.68(11)	C12	C7	C8	121.25(14)
C13	N1	C6	124.40(11)	C8	C7	O1	118.40(13)
C6	N1	C12	113.29(11)	C8	C9	C10	120.71(13)
C11	C12	N1	123.20(13)	C9	C8	C7	118.73(14)
C7	C12	N1	117.25(12)	C1	C6	N1	116.73(12)
C7	C12	C11	119.52(13)	C5	C6	N1	124.19(13)
O2	C13	N1	121.39(13)	C5	C6	C1	119.05(13)
O2	C13	C14	120.86(12)	C17	C16	C15	121.62(14)
N1	C13	C14	117.72(12)	C14	C15	C20	120.62(13)
C14	C19	C22	121.15(13)	C16	C15	C14	118.47(14)
C18	C19	C14	118.38(13)	C16	C15	C20	120.91(14)
C18	C19	C22	120.47(13)	C6	C5	C4	119.56(14)
C10	C11	C12	119.60(14)	C9	C10	C11	120.11(14)

Table 5 Bond Angles for DamrathM201007_0m.

Atom	Atom	Atom	Angle/°	Atom	Atom	Atom	Angle/°
C19	C14	C13	118.94(12)	C18	C17	C21	120.15(15)
C15	C14	C13	119.71(13)	C16	C17	C18	118.61(13)
C15	C14	C19	121.18(13)	C16	C17	C21	121.22(14)
C17	C18	C19	121.71(14)	C1	C2	C3	118.95(14)
O1	C1	C6	120.42(13)	C3	C4	C5	120.49(14)
C2	C1	O1	118.09(13)	C4	C3	C2	120.27(14)
C2	C1	C6	121.48(13)				

Table 6 Torsion Angles for DamrathM201007_0m.

A	B	C	D	Angle/°	A	B	C	D	Angle/°
O1	C1	C6	N1	-1.57(19)	C11	C12	C7	C8	3.0(2)
O1	C1	C6	C5	176.31(12)	C14	C19	C18	C17	1.9(2)
O1	C1	C2	C3	179.60(13)	C18	C19	C14	C13	-177.30(12)
O1	C7	C8	C9	178.90(12)	C18	C19	C14	C15	-2.0(2)
O2	C13	C14	C19	81.11(18)	C1	O1	C7	C12	32.20(17)
O2	C13	C14	C15	-94.29(17)	C1	O1	C7	C8	-148.18(13)
N1	C12	C11	C10	179.71(13)	C1	C6	C5	C4	4.7(2)
N1	C12	C7	O1	0.86(19)	C1	C2	C3	C4	3.4(2)
N1	C12	C7	C8	-178.75(13)	C7	O1	C1	C6	-31.81(17)
N1	C13	C14	C19	-97.35(16)	C7	O1	C1	C2	147.70(13)
N1	C13	C14	C15	87.24(17)	C7	C12	C11	C10	-2.1(2)
N1	C6	C5	C4	-177.56(14)	C8	C9	C10	C11	1.7(2)
C12	N1	C13	O2	6.3(2)	C6	N1	C12	C11	144.15(14)
C12	N1	C13	C14	-175.23(12)	C6	N1	C12	C7	-34.05(17)
C12	N1	C6	C1	34.24(17)	C6	N1	C13	O2	166.68(13)

Table 6 Torsion Angles for DamrathM201007_0m.

A	B	C	D	Angle/˚	A	B	C	D	Angle/˚
C12	N1	C6	C5	-143.52(14)	C6	N1	C13	C14	-14.9(2)
C12	C11	C10	C9	-0.2(2)	C6	C1	C2	C3	-0.9(2)
C12	C7	C8	C9	-1.5(2)	C6	C5	C4	C3	-2.3(2)
C13	N1	C12	C11	-53.41(19)	C15	C16	C17	C18	-0.2(2)
C13	N1	C12	C7	128.39(14)	C15	C16	C17	C21	-178.96(15)
C13	N1	C6	C1	-127.23(14)	C5	C4	C3	C2	-1.8(2)
C13	N1	C6	C5	55.0(2)	C10	C9	C8	C7	-0.9(2)
C13	C14	C15	C16	176.30(13)	C17	C16	C15	C14	0.1(2)
C13	C14	C15	C20	-2.8(2)	C17	C16	C15	C20	179.20(15)
C19	C14	C15	C16	1.0(2)	C22	C19	C14	C13	2.5(2)
C19	C14	C15	C20	-178.08(14)	C22	C19	C14	C15	177.87(13)
C19	C18	C17	C16	-0.8(2)	C22	C19	C18	C17	-177.98(13)
C19	C18	C17	C21	177.97(14)	C2	C1	C6	N1	178.93(12)
C11	C12	C7	O1	-177.41(13)	C2	C1	C6	C5	-3.2(2)

Table 7 Hydrogen Atom Coordinates (Å×10^4) and Isotropic Displacement Parameters (Å^2×10^3) for DamrathM201007_0m.

Atom	x	y	z	U(eq)
H11	8724.12	4560.53	9607.63	23
H18	8744.74	2237.41	5919.49	23
H9	6580.26	6546.81	10939.25	25
H8	4561.47	6771.97	9753.22	23
H16	6709.73	4961.29	4881.98	29
H5	5637.12	2967.62	7127.95	25
H10	8623.39	5418.2	10878.23	26
H22A	9914.59	3110.68	7945.71	34

Table 7 Hydrogen Atom Coordinates (Å×10^4) and Isotropic Displacement Parameters (Å^2×10^3) for DamrathM201007_0m.

Atom	x	y	z	U(eq)
H22B	9657.92	2042.31	7382.35	34
H22C	8367.88	2434.66	7851.8	34
H2	1677.21	5294.06	7408.03	25
H4	3128.46	2651.57	6367.72	31
H3	1169.15	3830.26	6480.06	31
H20A	5686.65	6008.95	6552.16	45
H20B	6174.39	6393	5709.52	45
H20C	7348.4	6512.46	6610.02	45
H21A	8744.64	2837.07	4385.96	51
H21B	7417.93	3664.96	3975.02	51
H21C	6987.1	2500.41	4267.68	51

Crystal structure determination of [DamrathM201007_0m]

Crystal Data for $C_{22}H_{19}NO_2$ (M =329.38 g/mol): monoclinic, space group P2$_1$/c (no. 14), a = 8.9727(5) Å, b = 12.4511(6) Å, c = 16.2174(9) Å, β = 103.216(2)°, V = 1763.82(16) Å^3, Z = 4, T = 100.0 K, μ(MoKα) = 0.079 mm^{-1}, $Dcalc$ = 1.240 g/cm^3, 40307 reflections measured (4.166° ≤ 2Θ ≤ 52.732°), 3607 unique (R_{int} = 0.0678, R_{sigma} = 0.0332) which were used in all calculations. The final R_1 was 0.0423 (I > 2σ(I)) and wR_2 was 0.1090 (all data).

Refinement model description

Number of restraints - 0, number of constraints - unknown.

Details:

1. Fixed Uiso
At 1.2 times of:
All C(H) groups
At 1.5 times of:
All C(H,H,H) groups
2.a Aromatic/amide H refined with riding coordinates:
C11(H11), C18(H18), C9(H9), C8(H8), C16(H16), C5(H5), C10(H10), C2(H2), C4(H4), C3(H3)
2.b Idealised Me refined as rotating group:
C22(H22A,H22B,H22C), C20(H20A,H20B,H20C), C21(H21A,H21B,H21C)

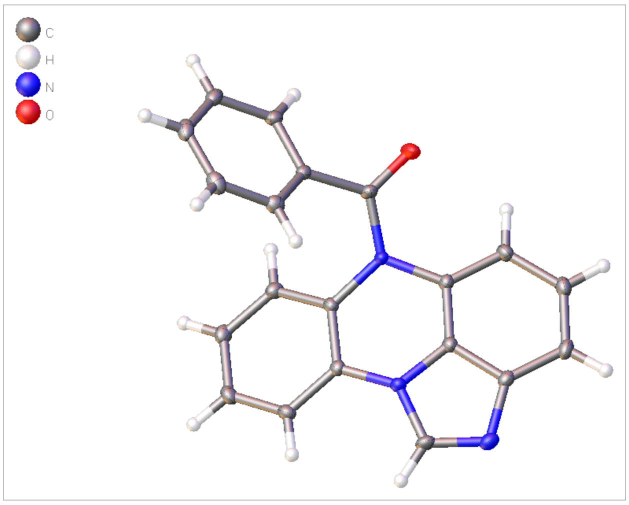

Table 1 Crystal data and structure refinement for KuczmeraT200917_0m.

CCDC 2288548

Identification code	KuczmeraT200917_0m
Empirical formula	$C_{20}H_{13}N_3O$
Formula weight	311.33
Temperature/K	100.0
Crystal system	monoclinic
Space group	$P2_1/c$
a/Å	10.0353(8)
b/Å	8.4931(8)
c/Å	17.5611(17)
α/°	90
β/°	99.888(3)
γ/°	90
Volume/Å^3	1474.5(2)
Z	4
ρ_{calc}g/cm^3	1.402
μ/mm^{-1}	0.089
F(000)	648.0
Crystal size/mm^3	0.23 × 0.23 × 0.17

Radiation	MoKα (λ = 0.71073)
2Θ range for data collection/°	4.708 to 60.996
Index ranges	-14 ≤ h ≤ 14, -12 ≤ k ≤ 12, -25 ≤ l ≤ 25
Reflections collected	43235
Independent reflections	4490 [R_{int} = 0.0431, R_{sigma} = 0.0250]
Data/restraints/parameters	4490/0/217
Goodness-of-fit on F^2	1.046
Final R indexes [I>=2σ (I)]	R_1 = 0.0457, wR_2 = 0.1128
Final R indexes [all data]	R_1 = 0.0595, wR_2 = 0.1215
Largest diff. peak/hole / e $Å^{-3}$	0.39/-0.36

Table 2 Fractional Atomic Coordinates ($\times 10^4$) and Equivalent Isotropic Displacement Parameters ($Å^2 \times 10^3$) for KuczmeraT200917_0m. U_{eq} is defined as 1/3 of of the trace of the orthogonalised U_{IJ} tensor.

Atom	x	y	z	U(eq)
O1	5207.8(9)	6354.1(13)	2226.8(5)	25.9(2)
N1	5742.4(9)	8032.1(12)	4949.1(5)	13.35(19)
C1	6477.8(12)	8683.2(14)	5612.5(6)	16.5(2)
N2	7760.5(10)	8900.1(12)	5572.0(6)	17.5(2)
C2	7914.8(11)	8301.8(13)	4848.9(6)	14.6(2)
N3	5021.9(9)	6631.1(12)	3495.4(5)	13.24(19)
C3	9045.8(12)	8065.0(14)	4490.6(7)	17.3(2)
C4	8817.6(12)	7308.1(15)	3779.8(7)	17.9(2)
C5	7530.7(12)	6786.9(14)	3405.2(7)	15.8(2)
C6	6411.9(11)	7023.2(13)	3758.1(6)	13.0(2)
C7	6666.2(11)	7771.7(13)	4468.7(6)	12.7(2)
C8	4187.8(11)	6439.6(13)	4079.2(6)	12.5(2)

Table 2 Fractional Atomic Coordinates (×10[4]) and Equivalent Isotropic Displacement Parameters (Å[2]×10[3]) for KuczmeraT200917_0m. U_{eq} is defined as 1/3 of of the trace of the orthogonalised U_{IJ} tensor.

Atom	x	y	z	U(eq)
C9	3063.1(11)	5448.7(13)	3956.2(6)	15.0(2)
C10	2196.9(11)	5367.3(14)	4494.4(7)	16.7(2)
C11	2480.1(12)	6213.8(15)	5180.1(7)	17.3(2)
C12	3651.6(12)	7109.3(14)	5340.2(6)	15.4(2)
C13	4499.8(11)	7219.0(13)	4795.0(6)	12.5(2)
C14	4489.6(12)	6615.4(14)	2707.0(6)	15.2(2)
C15	3026.5(11)	7018.1(13)	2467.3(6)	12.8(2)
C16	2269.4(11)	6179.1(14)	1859.3(6)	14.9(2)
C17	917.6(12)	6568.3(15)	1606.0(7)	17.9(2)
C18	338.2(12)	7808.7(15)	1945.9(7)	20.0(2)
C19	1099.8(13)	8668.9(14)	2538.4(7)	19.5(2)
C20	2440.6(12)	8268.2(13)	2803.8(7)	16.1(2)

Table 3 Anisotropic Displacement Parameters (Å[2]×10[3]) for KuczmeraT200917_0m. The Anisotropic displacement factor exponent takes the form: $-2\pi^2[h^2a^{*2}U_{11}+2hka^*b^*U_{12}+\ldots]$.

Atom	U_{11}	U_{22}	U_{33}	U_{23}	U_{13}	U_{12}
O1	16.8(4)	48.6(6)	12.6(4)	-5.5(4)	3.2(3)	4.7(4)
N1	12.1(4)	17.5(5)	10.3(4)	-1.9(3)	1.6(3)	-0.2(3)
C1	17.9(5)	19.3(5)	11.3(5)	-3.2(4)	-0.7(4)	-0.7(4)
N2	16.6(5)	20.1(5)	14.4(4)	-1.2(4)	-1.3(3)	-1.1(4)
C2	12.8(5)	16.0(5)	14.1(5)	1.4(4)	-0.5(4)	-0.2(4)
N3	10.3(4)	20.4(5)	9.0(4)	-2.1(3)	1.7(3)	-1.1(3)
C3	10.9(5)	20.5(5)	19.6(5)	3.6(4)	0.2(4)	-0.8(4)
C4	12.9(5)	22.5(6)	19.1(5)	3.4(4)	5.1(4)	1.6(4)

Table 3 Anisotropic Displacement Parameters ($Å^2×10^3$) for KuczmeraT200917_0m. The Anisotropic displacement factor exponent takes the form: $-2\pi^2[h^2a^{*2}U_{11}+2hka^*b^*U_{12}+…]$.

Atom	U_{11}	U_{22}	U_{33}	U_{23}	U_{13}	U_{12}
C5	14.5(5)	19.6(5)	13.8(5)	-0.1(4)	4.1(4)	2.1(4)
C6	10.8(5)	15.6(5)	12.3(5)	0.6(4)	1.2(4)	0.5(4)
C7	10.9(5)	15.2(5)	11.8(5)	1.0(4)	1.6(4)	0.7(4)
C8	11.6(5)	15.1(5)	11.0(5)	0.9(4)	2.7(4)	1.3(4)
C9	15.5(5)	15.5(5)	13.4(5)	0.8(4)	0.9(4)	-2.0(4)
C10	14.5(5)	17.5(5)	17.8(5)	4.0(4)	2.0(4)	-2.4(4)
C11	15.7(5)	21.0(5)	16.5(5)	3.7(4)	6.6(4)	0.9(4)
C12	16.3(5)	18.9(5)	11.5(5)	0.1(4)	3.5(4)	1.6(4)
C13	10.7(5)	14.3(5)	12.3(5)	0.7(4)	1.1(4)	1.1(4)
C14	13.6(5)	21.1(5)	10.5(5)	-2.3(4)	0.5(4)	0.0(4)
C15	12.1(5)	15.8(5)	10.4(5)	0.8(4)	1.8(4)	-1.3(4)
C16	15.8(5)	18.1(5)	10.6(5)	0.0(4)	1.5(4)	-1.1(4)
C17	14.9(5)	23.7(6)	13.9(5)	4.3(4)	-0.5(4)	-3.8(4)
C18	13.7(5)	24.9(6)	21.3(6)	10.3(5)	2.9(4)	2.0(4)
C19	20.3(6)	16.2(5)	24.0(6)	4.2(4)	9.1(5)	4.0(4)
C20	18.6(5)	14.0(5)	16.2(5)	-0.6(4)	4.3(4)	-1.7(4)

Table 4 Bond Lengths for KuczmeraT200917_0m.

Atom	Atom	Length/Å	Atom	Atom	Length/Å
O1	C14	1.2202(14)	C6	C7	1.3846(15)
N1	C1	1.3828(14)	C8	C9	1.3947(15)
N1	C7	1.3743(14)	C8	C13	1.4074(15)
N1	C13	1.4104(14)	C9	C10	1.3913(16)
C1	N2	1.3143(16)	C10	C11	1.3893(17)

Table 4 Bond Lengths for KuczmeraT200917_0m.

Atom	Atom	Length/Å	Atom	Atom	Length/Å
N2	C2	1.4009(15)	C11	C12	1.3879(17)
C2	C3	1.4034(16)	C12	C13	1.3893(15)
C2	C7	1.3897(15)	C14	C15	1.4959(15)
N3	C6	1.4313(14)	C15	C16	1.3951(15)
N3	C8	1.4398(14)	C15	C20	1.3934(16)
N3	C14	1.3957(14)	C16	C17	1.3929(16)
C3	C4	1.3878(17)	C17	C18	1.3868(18)
C4	C5	1.4158(16)	C18	C19	1.3883(19)
C5	C6	1.3870(15)	C19	C20	1.3886(17)

Table 5 Bond Angles for KuczmeraT200917_0m.

Atom	Atom	Atom	Angle/˚	Atom	Atom	Atom	Angle/˚
C1	N1	C13	133.15(10)	C9	C8	C13	118.17(10)
C7	N1	C1	105.24(9)	C13	C8	N3	121.03(10)
C7	N1	C13	118.08(9)	C10	C9	C8	120.54(10)
N2	C1	N1	113.36(10)	C11	C10	C9	120.29(11)
C1	N2	C2	105.07(9)	C12	C11	C10	119.95(11)
N2	C2	C3	133.02(11)	C11	C12	C13	119.68(10)
C7	C2	N2	108.79(10)	C8	C13	N1	117.10(10)
C7	C2	C3	118.04(10)	C12	C13	N1	121.78(10)
C6	N3	C8	116.76(9)	C12	C13	C8	121.02(10)
C14	N3	C6	120.52(9)	O1	C14	N3	121.09(10)
C14	N3	C8	122.34(9)	O1	C14	C15	120.98(10)
C4	C3	C2	116.64(10)	N3	C14	C15	117.84(10)
C3	C4	C5	124.00(11)	C16	C15	C14	118.54(10)

Table 5 Bond Angles for KuczmeraT200917_0m.

Atom	Atom	Atom	Angle/°	Atom	Atom	Atom	Angle/°
C6	C5	C4	119.40(11)	C20	C15	C14	121.34(10)
C5	C6	N3	129.88(10)	C20	C15	C16	119.98(10)
C7	C6	N3	114.58(9)	C17	C16	C15	119.72(11)
C7	C6	C5	115.54(10)	C18	C17	C16	120.00(11)
N1	C7	C2	107.49(9)	C17	C18	C19	120.32(11)
N1	C7	C6	126.01(10)	C18	C19	C20	119.97(11)
C6	C7	C2	126.38(10)	C19	C20	C15	119.97(11)
C9	C8	N3	120.79(10)				

Table 6 Torsion Angles for KuczmeraT200917_0m.

A	B	C	D	Angle/°	A	B	C	D	Angle/°
O1	C14	C15	C16	42.07(17)	C7	N1	C1	N2	2.37(13)
O1	C14	C15	C20	-133.75(13)	C7	N1	C13	C8	-12.01(15)
N1	C1	N2	C2	-2.42(14)	C7	N1	C13	C12	164.33(10)
C1	N1	C7	C2	-1.25(12)	C7	C2	C3	C4	-0.19(16)
C1	N1	C7	C6	174.89(11)	C8	N3	C6	C5	156.27(12)
C1	N1	C13	C8	-167.29(12)	C8	N3	C6	C7	-24.72(14)
C1	N1	C13	C12	9.05(19)	C8	N3	C14	O1	-160.05(12)
C1	N2	C2	C3	-173.69(13)	C8	N3	C14	C15	23.32(16)
C1	N2	C2	C7	1.52(13)	C8	C9	C10	C11	-3.25(17)
N2	C2	C3	C4	174.67(12)	C9	C8	C13	N1	171.17(10)
N2	C2	C7	N1	-0.13(13)	C9	C8	C13	C12	-5.20(16)
N2	C2	C7	C6	-176.25(11)	C9	C10	C11	C12	-1.87(18)
C2	C3	C4	C5	0.47(18)	C10	C11	C12	C13	3.34(17)
N3	C6	C7	N1	5.76(16)	C11	C12	C13	N1	-175.96(11)

Table 6 Torsion Angles for KuczmeraT200917_0m.

A	B	C	D	Angle/°	A	B	C	D	Angle/°
N3	C6	C7	C2	-178.82(10)	C11	C12	C13	C8	0.24(17)
N3	C8	C9	C10	-174.65(10)	C13	N1	C1	N2	159.89(12)
N3	C8	C13	N1	-7.51(15)	C13	N1	C7	C2	-162.81(10)
N3	C8	C13	C12	176.12(10)	C13	N1	C7	C6	13.33(16)
N3	C14	C15	C16	-141.30(11)	C13	C8	C9	C10	6.68(16)
N3	C14	C15	C20	42.89(16)	C14	N3	C6	C5	-30.64(18)
C3	C2	C7	N1	175.90(10)	C14	N3	C6	C7	148.37(11)
C3	C2	C7	C6	-0.22(18)	C14	N3	C8	C9	34.95(16)
C3	C4	C5	C6	-0.36(19)	C14	N3	C8	C13	-146.41(11)
C4	C5	C6	N3	178.95(11)	C14	C15	C16	C17	-177.68(10)
C4	C5	C6	C7	-0.05(16)	C14	C15	C20	C19	176.34(11)
C5	C6	C7	N1	-175.09(11)	C15	C16	C17	C18	1.48(17)
C5	C6	C7	C2	0.34(17)	C16	C15	C20	C19	0.59(17)
C6	N3	C8	C9	-152.09(11)	C16	C17	C18	C19	0.07(18)
C6	N3	C8	C13	26.54(15)	C17	C18	C19	C20	-1.30(18)
C6	N3	C14	O1	27.26(17)	C18	C19	C20	C15	0.96(17)
C6	N3	C14	C15	-149.38(11)	C20	C15	C16	C17	-1.81(17)

Table 7 Hydrogen Atom Coordinates (Å×10^4) and Isotropic Displacement Parameters (Å^2×10^3) for KuczmeraT200917_0m.

Atom	x	y	z	U(eq)
H1	6093.68	8947.58	6053.89	20
H3	9923.41	8405.6	4723.07	21
H4	9570.79	7128.8	3529.14	21
H5	7434.43	6279.91	2916.65	19
H9	2886.95	4824.2	3501.23	18

Table 7 Hydrogen Atom Coordinates (Å×10^4) and Isotropic Displacement Parameters (Å^2×10^3) for KuczmeraT200917_0m.

Atom	x	y	z	U(eq)
H10	1408.3	4730.06	4392.54	20
H11	1872.22	6179.67	5539.12	21
H12	3872.46	7644.55	5820.35	19
H16	2674.13	5345.87	1619.17	18
H17	392.51	5984.08	1200.29	21
H18	-584.2	8070.98	1772.44	24
H19	703.68	9531.16	2762.26	23
H20	2958.28	8846.2	3214.56	19

Crystal structure determination of [KuczmeraT200917_0m]

Crystal Data for $C_{20}H_{13}N_3O$ (M =311.33 g/mol): monoclinic, space group P2_1/c (no. 14), a = 10.0353(8) Å, b = 8.4931(8) Å, c = 17.5611(17) Å, β = 99.888(3)°, V = 1474.5(2) Å^3, Z = 4, T = 100.0 K, μ(MoKα) = 0.089 mm^{-1}, $Dcalc$ = 1.402 g/cm^3, 43235 reflections measured (4.708° ≤ 2Θ ≤ 60.996°), 4490 unique (R_{int} = 0.0431, R_{sigma} = 0.0250) which were used in all calculations. The final R_1 was 0.0457 (I > 2σ(I)) and wR_2 was 0.1215 (all data).

Refinement model description

Number of restraints - 0, number of constraints - unknown.

Details:

1. Fixed Uiso
At 1.2 times of:
All C(H) groups
2.a Aromatic/amide H refined with riding coordinates:
C1(H1), C3(H3), C4(H4), C5(H5), C9(H9), C10(H10), C11(H11), C12(H12), C16(H16), C17(H17), C18(H18), C19(H19), C20(H20)

References

1. Boelke, A.; Kuczmera, T. J.; Caspers, L. D.; Lork, E.; Nachtsheim, B. J. *Org. Lett.* **2020,** *22*, 7261–7266. doi:10.1021/acs.orglett.0c02593
2. Jiang, M.; Guo, J.; Liu, B.; Tan, Q.; Xu, B. *Org. Lett.* **2019,** *21*, 8328–8333. doi:10.1021/acs.orglett.9b03106
3. Li, E.; Xu, X.; Li, H.; Zhang, H.; Xu, X.; Yuan, X.; Li, Y. *Tetrahedron* **2009,** *65*, 8961–8968. doi:10.1016/j.tet.2009.08.075

4. Kehl, A.; Schupp, N.; Breising, V. M.; Schollmeyer, D.; Waldvogel, S. R. *Chem. Eur. J.* **2020,** *26*, 15847–15851. doi:10.1002/chem.202003430
5. Spratt, M. P.; Dorn, H. C. *Anal. Chem.* **1984,** *56*, 2038–2043. doi:10.1021/ac00276a014
6. Li, H.; Li, H.; Gu, J.; He, F.; Peng, H.; Tao, Y.; Tian, D.; Yang, Q.; Li, P.; Zheng, C.; Huang, W.; Chen, R. *Chem. Sci.* **2021,** *12*, 3580–3586. doi:10.1039/D0SC06025A
7. Lu, C.; Markina, N. A.; Larock, R. C. *J. Org. Chem.* **2012,** *77*, 11153–11160. doi:10.1021/jo3021727
8. Cai, S.; Shi, H.; Li, J.; Gu, L.; Ni, Y.; Cheng, Z.; Wang, S.; Xiong, W.-W.; Li, L.; An, Z.; Huang, W. *Adv. Mater.* **2017,** *29*, 1701244. doi:10.1002/adma.201701244
9. Kayama, S.; Tabata, H.; Takahashi, Y.; Tani, N.; Wakamatsu, S.; Oshitari, T.; Natsugari, H.; Takahashi, H. *Tetrahedron* **2015,** *71*, 7046–7053. doi:10.1016/j.tet.2015.06.097
10. Buchspies, J.; Rahman, M. M.; Szostak, R.; Szostak, M. *Org. Lett.* **2020,** *22*, 4703–4709. doi:10.1021/acs.orglett.0c01488
11. Liu, Y.; Zhan, G.; Fang, P.; Liu, Z.; Bian, Z.; Huang, C. *J. Mater. Chem. C* **2017,** *5*, 12547–12552. doi:10.1039/C7TC04634K
12. Liu, Z.; Larock, R. C. *Org. Lett.* **2004,** *6*, 3739–3741. doi:10.1021/ol048564l
13. Day, C. S.; Ton, S. J.; Kaussler, C.; Vrønning Hoffmann, D.; Skrydstrup, T. *Angew. Chem. Int. Ed. Engl.* **2023,** *62*, e202308238. doi:10.1002/anie.202308238
14. Damrath, M.; Caspers, L. D.; Duvinage, D.; Nachtsheim, B. J. *Org. Lett.* **2022,** *24*, 2562–2566. doi:10.1021/acs.orglett.2c00691
